# Employing AC and DC Electrolysis to Modulate Electroenzymatic Pathways for Efficient and Stereoselective H‑D Exchange

**DOI:** 10.1021/jacs.6c02284

**Published:** 2026-05-28

**Authors:** Wassim El Housseini, Rokas Gerulskis, Nibedita Behera, Huaijun Guan, Rohit G. Jadhav, Zachary A. Nguyen, Egor Baiarashov, Michael A. Pence, Vamshi Krishna Kamaja, Trevor Larkin, Long Luo, Shelley D. Minteer

**Affiliations:** † Kummer Institute Center for Resource Sustainability, 14717 Missouri University of Science and Technology, Rolla, Missouri 65409, United States; ‡ Department of Chemistry, 7060 University of Utah, Salt Lake City, Utah 84112, United States

## Abstract

Stereoselective hydrogen isotope exchange (HIE) at chiral centers is an increasingly important strategy for preparing labeled molecules, yet its practical implementation depends on reliable control of nicotinamide cofactor regeneration. Here we introduce a redox-programmable electroenzymatic platform for stereoselective HIE based on electrode-controlled manipulation of the nicotinamide cofactor state. A wired ferredoxin–NADP^+^ reductase (FNR) electrode enables reversible electrochemical interconversion of NADP^+^ and its deuterated reduced form (NADPD) directly from D_2_O. Coupling this cofactor cycling with enantioselective alcohol dehydrogenases (ADHs) establishes a reversible alcohol–ketone redox manifold that drives efficient and stereoselective H-D exchange at chiral alcohols. Stereochemical outcomes are programmed by the combined choice of paired electrolysis mode and enzyme configuration: direct-current electrolysis enables stereochemical editing with stereocomplementary ADHs, whereas alternating-current electrolysis supports rapid bidirectional cycling with a single ADH to achieve stereoretentive labeling. This strategy affords near-quantitative deuterium incorporation with high enantiopurity across a broad range of secondary alcohols.

## Graphical abstract

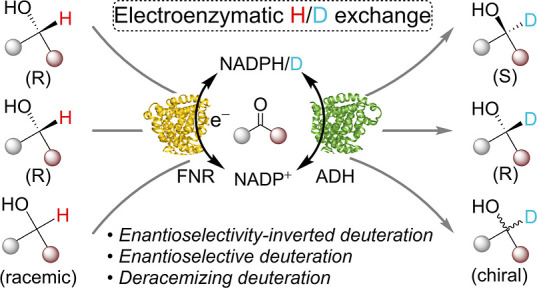

## Introduction

The incorporation of Deuterium (D) into organic molecules has become a valuable strategy across disciplines ranging from chemistry and biology to analytical sciences and environmental monitoring.^1–4^ Introducing D in place of protium can influence far more than molecular mass: it can reshape nuclear spin properties relevant to NMR spectroscopy, shift vibrational and optical behavior, alter neutron-scattering characteristics, and in some cases modify solubility.^1^ One of the most consequential effects is the kinetic isotope effect (KIE), wherein the rate of C-D bond cleavage is markedly slower than for C–H analogues.^2^ This phenomenon has been widely exploited to elucidate the mechanistic features of chemical reactions and enzyme-mediated transformations.^5,6^ In recent years, the KIE has also gained attention for its potential to enhance the performance of pharmaceutical drugs.^7,8^ Selective deuteration can increase resistance to metabolic pathways such as oxidation or racemization, thereby improving the stability of drug molecules, lowering effective dose requirements, and reducing the formation of undesired or toxic byproducts.^2,7,9^ These emerging therapeutic advantages have intensified interest in developing reliable and selective methods for introducing deuterium into structurally complex and biologically relevant compounds.^10^

The growing prominence of deuterated pharmaceuticals has created a strong demand for catalytic systems capable of delivering position-selective deuteration.^7^ Such transformations are commonly achieved through hydrogen isotope exchange (HIE) or halogen–deuterium exchange processes, in which a preformed molecule is exposed to D_2_O or D_2_ to introduce D at targeted sites.^7,9,11^ Advances in these methodologies have significantly improved the regiocontrol of isotopic installation. Despite these achievements, methods that insert D with defined enantioselectivity remain comparatively underdeveloped. Only a small number of catalytic systems have been reported for asymmetric HIE,^12–14^ and such postsynthetic approaches frequently compromise the enantiomeric integrity of the substrate.^2,7^ Furthermore, most established chemical HIE methods rely on harsh conditions, including high temperatures, strong acids or bases, or precious-metal catalysts, limiting their compatibility with complex and functionalized molecules.^15,16^ For example, several transition-metal-catalyzed HIE methods based on Pd, Pt, Ir, and Ru catalysts have been reported to require elevated temperatures or the use of strongly coordinating directing groups to achieve efficient and site-selective labeling.^16,17^ In many cases, these transformations are conducted at temperatures in the range of 80–200 °C and display limited functional-group tolerance toward pharmaceutically relevant motifs.^18^ Conventional acid- or base-promoted benzylic HIE approaches similarly rely on strong reagents and frequently lead to partial or complete racemization.^19^ Collectively, these limitations underscore the need for the development of mild, tunable, and stereoselective HIE methodologies.

Recent electrocatalytic deuteration strategies have shown that electrochemical interfaces can generate and control reactive D-transfer pathways from deuterated media. For example, Lei and co-workers reported a boron-cluster-mediated radical HIE system in which anodically generated ${\left[B_{10}H_{10}\right]}^{\cdot -}$ abstracts H/D atoms and the resulting ${\left[B_{10}H_{10}H/D\right]}^{-}$ intermediate is reduced cathodically to enable radical HIE exchange at C­(sp^3^)–H sites.^20^ Separately, Bu, Lei, and co-workers demonstrated reductive deuteration of arenes and heteroarenes using D_2_O and a nitrogen-doped Ru electrode, where electrochemically generated Ru-D species mediate dearomative reduction to highly deuterated saturated products.^21^ These studies highlight the growing power of electrocatalysis for isotope labeling, while also underscoring the complementary need for stereoselective controlled platforms capable of inserting D at defined chiral centers under mild conditions. Biocatalytic HIE offers an appealing alternative, leveraging the exquisite chemo- and stereoselectivity of enzymes.^22^ Nicotinamide-dependent dehydrogenases can drive reversible oxidation–reduction at C–H bonds with outstanding control of stereochemistry.^23,24^ However, deploying NAD­(P)­H-dependent enzymes for D labeling requires continuous regeneration of a reduced nicotinamide cofactor.^25–29^ Conventional approaches often rely on sacrificial substrates (e.g., glucose, isopropanol, or formate), but their deuterated analogues are costly and consumed stochastically, complicating reactor design and diminishing atom economy.^27,29^ Notably, continuous cycling of nicotinamide cofactors has been demonstrated in both enzymatic and electrochemical systems. For example, hydrogenase-driven platforms have enabled regeneration of deuterated nicotinamide cofactors using molecular hydrogen as the electron donor, thereby supporting stereoselective reductive deuteration reactions.^30^ In parallel, electrochemical regeneration of NAD­(P)H is well established and widely used to drive enzymatic transformations.^31^ Nevertheless, systems that combine electrochemical control with reversible cycling of deuterated cofactors directly from D_2_O remain comparatively rare.^25,26,28,29^ There is thus a strong incentive to develop electrochemical platforms capable of regenerating and dynamically cycling deuterated NADPD directly from D_2_O, an abundant, inexpensive, and benign deuterium source.

Herein, a new electroenzymatic strategy for HIE has been introduced that overcomes this long-standing challenge through indirect electrochemical regeneration of nicotinamide cofactor. Central to the platform is ferredoxin-NADP^+^ reductase (FNR),^32^ electrically wired to mediate continuous, bidirectional NADP^+^/NADPD interconversion.^33–36^ Under reductive bias, FNR couples electrons from the electrode with deuterons from D_2_O to produce NADPD. Under oxidative bias, FNR performs the reverse hydride-removal step, regenerating NADP^+^ (Figure 1). This reversible, electrochemically gated NADP^+^/NADPD cycle enables a catalytic sequence in which substrate oxidation under oxidative bias produces a ketone intermediate that is subsequently reduced under reductive bias to furnish a deuterated alcohol. By pairing FNR with a suitable NADPH-dependent alcohol dehydrogenase (ADH), HIE can be conducted under mild aqueous conditions while tolerating a broad range of functional groups. Because hydrogen transfer occurs through an enzymatic hydride mechanism, the stereochemical outcome is dictated solely by the choice of ADH.^37,38^ A single enantioselective ADH affords near-complete retention of configuration, whereas sequential oxidation and reduction catalyzed by stereocomplementary ADHs enables inversion. Racemic substrates can likewise undergo electroenzymatic deracemization to yield enantioenriched deuterated products (Figure 1).

**Figure 1:**
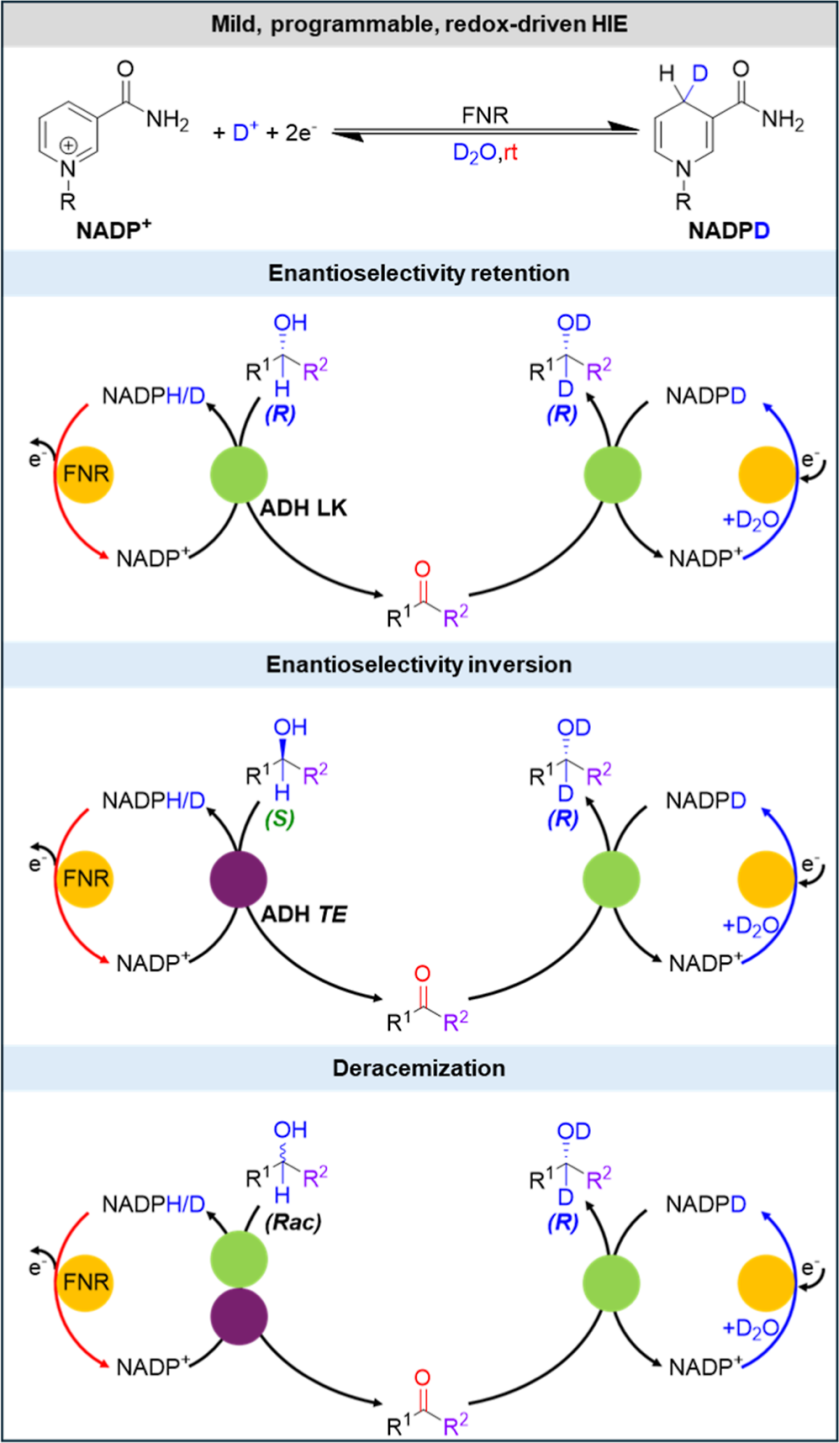
Concept for redox-programmable electroenzymatic hydrogen isotope exchange (HIE). Mild, redox-driven HIE is enabled through electroenzymatic NADP^+^/NADPD cycling mediated by ferredoxin-NADP^+^ reductase (FNR) in D_2_O. Coupling electrochemical cofactor control with enantioselective alcohol dehydrogenases (ADHs) allows precise control over stereochemical outcomes, including enantioselective retention, inversion, or deracemization, under ambient conditions.

Achieving these stereochemical transformations requires an electrochemical environment that controls the order of oxidation and reduction relative to the ADHs. This is accomplished through direct-current (DC) paired electrolysis, which spatially separates the two half-reactions. Under oxidative bias, FNR oxidizes NADPH (or NADPD) to NADP^+^ at the anode, driving ADH-mediated substrate oxidation; at the cathode, FNR reduces NADP^+^ to NADPD, enabling stereoselective reduction by a second ADH. This arrangement enforces the intended reaction sequence, continuously recycles the cofactor pool, and provides access to retention, inversion, or deracemization. Furthermore, the overall efficiency depends on both electrode kinetics and the mass-transfer rate at which the intermediate formed at the anode reaches the cathode.

While DC paired electrolysis affords stereodivergent control, an alternative regime emerges when redox events are enforced temporally rather than spatially. In the alternating-current (AC) mode, each electrode oscillates between oxidative and reductive bias, causing both half-reactions to occur sequentially at the same interface.^39–41^ Intermediates generated in one-half-cycle are consumed in the next, and complementary chemistry occurs simultaneously at the counter electrode, minimizing local depletion of substrate or cofactor. Because both steps proceed through the same ADH, AC electrolysis intrinsically preserves the enzyme’s stereochemical preference and is therefore suited exclusively to retentive HIE configuration. This approach supports fast and stereoretentive deuteration across a broad scope of aromatic secondary alcohols, including those bearing ortho substituents, extended conjugation, and heterocyclic or drug-like motifs. Engineered alcohol dehydrogenase variants further extend the platform’s applicability to sterically hindered and pharmaceutically relevant substrates.

## Results and Discussion

### Mechanistic Insights into Reversible Redox Conversion of Alcohol and Ketone

The ability of FNR to catalyze both the electroenzymatic oxidation of NADPH and the electroenzymatic reduction of NADP^+^ (Figure 2A) was evaluated using cyclic voltammetry (CV, 2 mV·s^–1^) and chronoamperometry (CA) at a pH of 9 (Figure S4). FNR was immobilized by drop-casting onto an ITO-modified electrode (FNR@CP-ITO_m_) (Figure S1). The FNR redox couple appeared at −0.38 V vs SHE (**curve b**, Figure 2B), whereas no corresponding redox feature was observed on a control ITO_m_ electrode (**curve a**, Figure 2B). The FNR-modified electrode was found to be highly stable, retaining its redox response over 150 continuous CV cycles (≈4 h) under nonturnover conditions (Figure S5). The electroenzymatic interconversion of NADP^+^ and NADPH was further investigated by CV. Upon addition of 3 mM NADP^+^, both cathodic and anodic current densities increased, indicating that FNR immobilized on the ITO-modified electrode efficiently catalyzes the bidirectional regeneration of the cofactor. Furthermore, bulk electrolysis experiments yielded apparent Michaelis–Menten constants (*K*_m_) of 1.7 ± 0.2 mM for NADP^+^ and 1.9 ± 0.1 mM for NADPH, confirming efficient catalytic turnover and reversible electroenzymatic cycling at the FNR-modified electrode (Figure S6). The apparent *K*_m_ values are higher than those reported for soluble FNRs, consistent with diffusional and interfacial effects associated with immobilization, while high turnover frequencies confirm preserved catalytic competence.^42^

**Figure 2:**
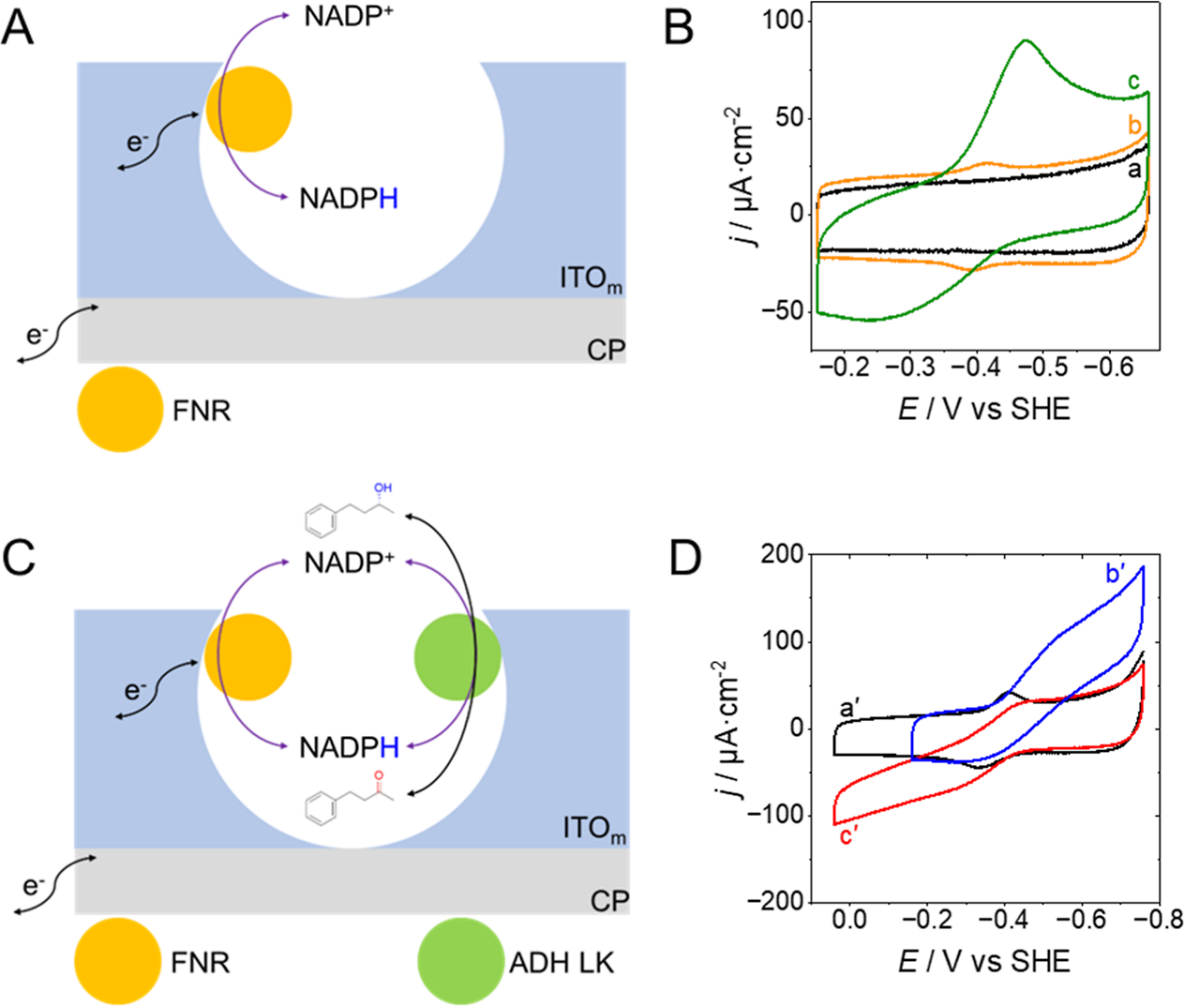
Mechanistic insights into the reversible redox conversion of alcohols and ketones mediated by electroenzymatic NADP^+^/NADPH cycling. (A) Schematic representation of the electroenzymatic oxidation and reduction of NADP^+^/NADPH catalyzed by FNE@CP-ITO_m_ (3 nmol FNR). (B) CV experiments showing: (a) CP-ITO_m_ and FNE@CP-ITO_m_ in (b) the absence and (c) the presence of 3 mM NADP^+^. (C) Illustration of the coupled FNR-ADH LK@CP-ITO_m_ system (3 nmol FNR-1 nmol ADH LK) enabling reversible interconversion between 4-phenyl-2-butanone (**1**) and (R)-4-phenyl-2-butanol (**1**′) through continuous NADP^+^/NADPH cycling. (D) CVs of FNR–ADH LK@ITO_m_/CP recorded in the presence of 50 μM NADP^+^ and 50 μM NADPH (a′), after addition of 10 mM **1** (b′), and after addition of 10 mM **1**′ (c′).

Building on the confirmed bidirectional cofactor regeneration, the FNR-modified electrode was subsequently coupled with Lactobacillus kefir alcohol dehydrogenase (ADH LK).^43,44^ Enzymatic activity assays established that ADH LK is selective for (*R*)-4-phenyl-2-butanol (**1**′) in the presence of NADP^+^ and for the corresponding ketone, 4-phenyl-2-butanone (**1**), in the presence of NADPH, and exhibits comparable turnover numbers for alcohol oxidation and ketone reduction (*k*_cat,ox_ = 29 s^–1^ and *k*_cat,red_ = 32 s^–1^). On this basis, the coupled system was designed to enable electroenzymatic mediation of the reversible redox interconversion between **1** and **1**′, as schematically illustrated in Figure 2C. Figure 2D shows the CV profiles of the bifunctional system (FNR-ADH LK@CP-ITO_m_). In the presence of 100 μM NADP^+^/NADPH (**curve a′**), a quasi-reversible redox process centered around −0.38 V vs SHE is observed, corresponding to the electrochemical interconversion of the NADP^+^/NADPH couple mediated by FNR. Upon addition of 10 mM **1** (**curve b′**), the reduction current increased gradually, indicating the reductive catalytic turnover of NADP^+^ to NADPH, which in turn drives the stereoselective reduction of **1** to **1**′ by ADH LK. Conversely, the introduction of 10 mM **1**′ (**curve c′**) led to a pronounced anodic response, consistent with the oxidation of NADPH to NADP^+^ coupled to the dehydrogenation of the alcohol to its corresponding ketone. These results demonstrate efficient electroenzymatic coupling between FNR and ADH LK, enabling reversible, directionally controlled redox conversion of alcohols and ketones via continuous NADP^+^/NADPH cycling at the electrode interface. Interestingly, the combined presence of both substrates, **1** and **1′**, together with the NADP^+^/NADPH couple, resulted in a marked increase in both anodic and cathodic current densities compared to FNR alone (Figure S7). This symmetric current enhancement confirms efficient bidirectional electron flow between the enzymatic cascade and the electrode, demonstrating reversible electroenzymatic control of alcohol–ketone interconversion. Bulk electrolysis experiments further corroborated these findings, yielding apparent turnover frequencies of 26.5 ± 2.1 s^–1^ for the reduction and 25.4 ± 1.3 s^–1^ for the oxidation process (Figure S8). These values agree with the *k*_cat_ parameters obtained for ADH LK enzymatic activity in homogeneous solution, confirming that the immobilized enzyme retains its intrinsic catalytic performance upon integration into the electrochemical system. The close correspondence between electrochemical and enzymatic turnover rates underscores that both half-reactions proceed with comparable kinetic efficiency, thereby validating the reversibility and dynamic balance of the coupled FNR-ADH LK cascade.

### Electroenzymatic Coupling of NADPD Regeneration with the Biocatalytic Reductive Deuteration of a Ketone

Electroenzymatic coupling of NADPD regeneration with the biocatalytic deuteration of the ketone was achieved by performing the reaction in Tris-DCl buffer (pD 9.0), where FNR electroenzymatically catalyzed the in situ regeneration of NADPD. The regenerated cofactor subsequently enabled ADH LK-catalyzed reduction of **1** to [^2^H] **1**′ (Figure 3A).

**Figure 3:**
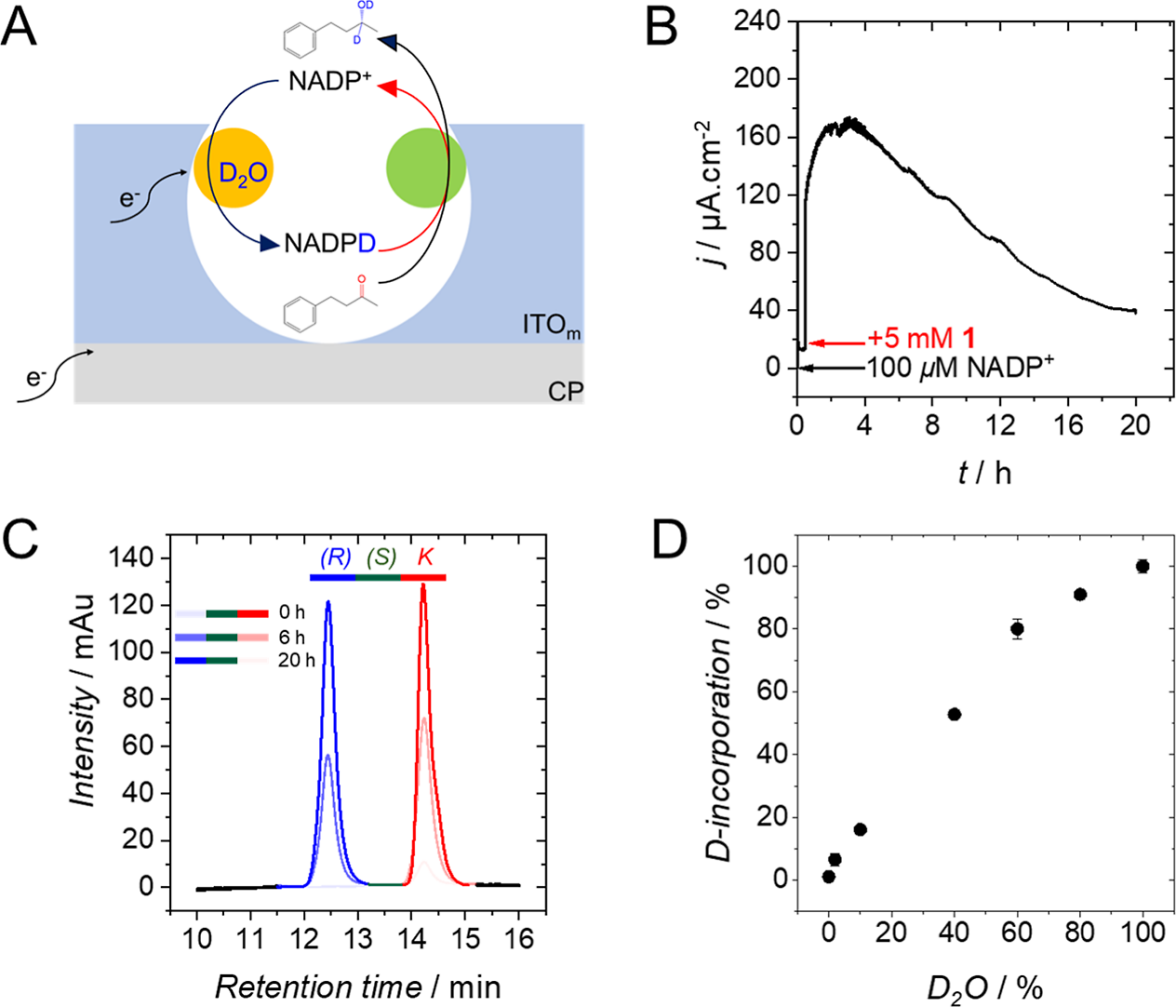
Electroenzymatic deuteration of 1 via coupled FNR-ADH LK catalysis. (A) Schematic representation of the electroenzymatic system showing in situ regeneration of NADPD from NADP^+^ in Tris-DCl buffer by FNR and its subsequent utilization by ADH LK to convert **1** to [^2^H] **1**′. (B) Chronoamperometric response recorded at −0.6 V vs SHE for FNR-ADH LK@ITO_m_-CP (3 nmol FNR-1 nmol ADH LK) showing current enhancement after the addition of 100 μM NADP^+^ followed by 5 mM **1**. (C) Time-dependent chiral LC chromatograms confirming the selective formation of **1**′ during bulk electrolysis. (D) Dependence of deuterium incorporation on the D_2_O content of the electrolyte, determined by MS analysis.

Figure 3B shows bulk electrolysis experiments carried out at −0.6 V vs SHE. Upon sequential addition of 100 μM NADP^+^ followed by 5 mM of **1** to the electrolyte, a pronounced increase in catalytic current was observed, corresponding to enhanced local cofactor recycling mediated by ADH LK during catalysis. Under all D_2_O proportions, the faradaic efficiency during the first 2 h of electrolysis remained close to 100% and slightly decreased to 89 ± 4% at the end of the experiment, while the enantioselectivity toward the (*R*)-alcohol—determined by chiral LC analysis—was 100% (Figure 3C). After 20 h of bulk electrolysis, nearly complete substrate conversion was achieved with a production rate of 0.45 ± 0.03 μmol·h^–1^ (Figure 3C). Interestingly, the dependence of deuterium incorporation on the D_2_O content of the electrolyte provides quantitative evidence that the isotopic label originates from the solvent via electroenzymatic NADPD regeneration. A positive correlation between the D_2_O fraction and the degree of product deuteration—determined by MS analysis—was observed, reaching >95% incorporation in 100% D_2_O (Figure 3D). Consistent with these findings, representative ^1^H NMR spectra recorded at varying D_2_O fractions show progressive disappearance of the methine proton signal at the alcohol stereogenic center and collapse of the adjacent methyl doublet to a singlet, confirming formation of the α-deuterated alcohol (Supporting Information, Section 13.1). Importantly, control experiments performed in the reaction medium demonstrate that the ketone intermediate, 4-phenyl-2-butanone, does not undergo detectable H–D exchange at the β-position in the absence of electrolysis (Supporting Information, Figure 4A). This trend demonstrates that the reduction of NADP^+^ at the FNR-modified electrode proceeds through efficient exchange of buffer protons for deuterons during hydride formation at the flavin site, yielding NADPD in correlation with the isotopic composition of the buffer. The subsequent ADH-catalyzed reduction of the ketone thus transfers the deuteride from NADPD to the substrate with high enantioselectivity, leading to stoichiometric incorporation of D into [^2^H] **1**′. The correlation plot exhibits a measurable curvature (Figure 3D), indicating that the extent of D incorporation in the product exceeds that expected from the bulk isotopic composition of the solution. However, this deviation remains well below that associated with a primary kinetic isotope effect, consistent with D not being involved in the rate-determining step of the catalytic cycle. Collectively, these results confirm that the coupled FNR-ADH LK system enables direct electrochemical control over the isotopic composition of the nicotinamide cofactor and, consequently, precise modulation of substrate deuteration. Encouraged by the efficient and stereoselective reduction to [^2^H] **1**′, the reverse oxidation of a racemic 4-phenyl-2-butanol (**3**′) mixture was subsequently examined. Under the same electroenzymatic conditions, only **1**′ was oxidized to **1**, yielding a product with 100% enantiomeric excess and confirming the strict (*R*)-selectivity of ADH LK in both oxidative and reductive directions (Supporting Information, Section 7).

### Paired Electrolysis Approach for Electroenzymatic Deuteration of the α-position of Alcohols

After establishing the bidirectional selectivity and efficiency of ADH LK, we next implemented a paired electrolysis configuration to couple the anodic oxidation and cathodic reduction steps within a single electrochemical system.

### Direct-Current Paired Electrolysis for HIE at the α-Position of Alcohols

To integrate both the oxidative and reductive half-reactions into a single system, a DC paired electrolysis configuration was first investigated (Figure 4A–D). In this setup, oxidation of **1**′ occurred at the anode, considered as the WE, while the regeneration of NADPD coupled to the reduction of **1** proceeded at the cathode in Tris-DCl buffer (Figure 4A).

**Figure 4:**
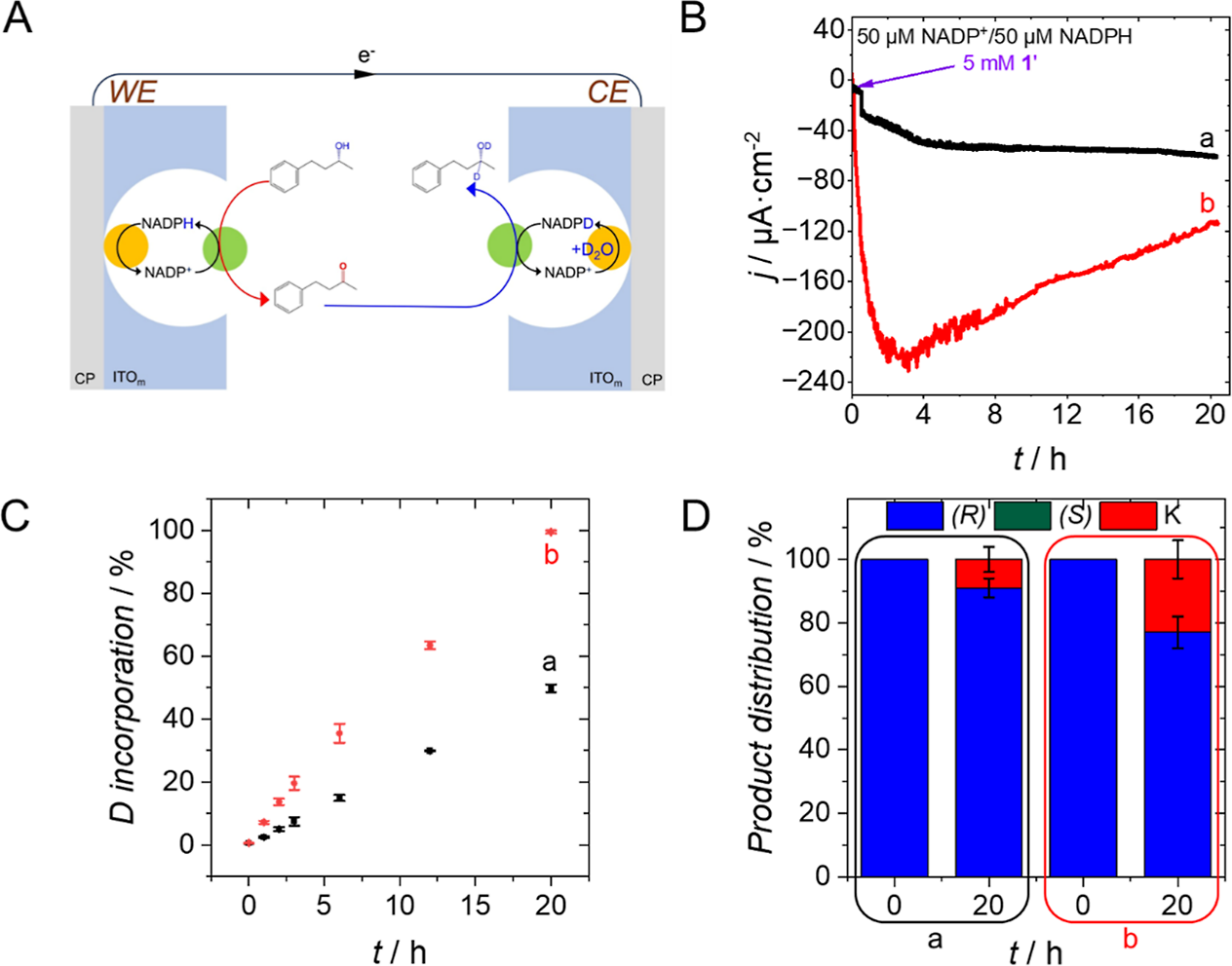
DC paired electrolysis strategies for the electroenzymatic deuteration of **1′**. (A) Schematic illustration of the electroenzymatic deuteration pathway proceeding through the oxidation of **1**′ to **1** at the anode, followed by electroenzymatic reduction of the ketone to [^2^H] **1**′ at the cathode via in situ NADPD regeneration. (B) Bulk electrolysis carried out at 0 V vs SHE in the presence of 50 μM NADP^+^, 50 μM NADPH, and 5 mM **1**′, comparing two electrode configurations: (a) equal working and counter electrode areas and loadings (3 nmol FNR-1 nmol ADH LK), and (b) a counter electrode four times larger than the working electrode with four times higher loading. (C) Time-dependent D incorporation determined by MS analysis. (D) Enantiomeric composition determined by chiral LC analysis for both configurations in (B) (Blue, **1′**; green, (*S*)-4-phenyl-2-butanol (**2**′); red, **1**).

Although these results demonstrate HIE with 100% ee, both the steady-state current and the rate of deuterated product formation were modest compared to single-electrode electrolysis experiments performed separately for oxidation or reduction (Figure 3B). This behavior reflects a kinetic imbalance between the two half-reactions under symmetric electrode configurations, in which oxidation of **1**′ proceeds more rapidly than the reductive conversion of the ketone intermediate. Because D incorporation occurs exclusively during the ketone reduction step, mass transfer of the ketone between the electrodes and the intrinsically slower reductive kinetics at the counter electrode impose a limitation on overall HIE efficiency.

To address this mismatch, an asymmetric configuration was implemented in which the counter electrode area was increased 4-fold relative to the working electrode. Under these conditions, the steady-state current increased substantially (≈220 μA·cm^–2^, **curve b**, Figure 4B), approaching values observed in single-electrode systems, and the deuteration yield improved to 97 ± 1% after 20 h with complete (*R*)-selectivity (Figure 4C). The enlarged cathodic surface compensates for slower ketone-reduction kinetics and facilitates efficient capture and conversion of the ketone intermediate, thereby restoring redox balance between oxidation and reduction and leading to enhanced deuteration efficiency and improved operational stability. Notably, a fraction of the deuterated alcohol is formed enzymatically through internal cofactor recycling, as oxidation of the alcohol generates NADPD that is subsequently consumed by ADH LK during ketone reduction to the deuterated alcohol (and vice versa), resulting in effective catalytic amplification whereby the apparent Faradaic efficiency of the system exceeds 100%. Moreover, to further assess the chemical selectivity of the paired system, product composition was analyzed for the collected samples during electrolysis. As shown in Figure 4D the product distribution confirms enantioselective retention with transient accumulation of ketone intermediates during electrolysis, reflecting the dynamic balance between alcohol oxidation and ketone reduction within the coupled redox cycle.

### Alternating-Current (AC) Paired Electrolysis for Efficient HIE at Chiral Alcohol Centers

While the optimized DC electrolysis configuration with asymmetric electrodes enables efficient cofactor recycling and selective deuteration, its performance is intrinsically limited by mass transfer of intermediate **1** between spatially separated electrodes, which constrains current density and reaction rate. To circumvent this limitation, we implemented AC electrolysis (Figure 5A), in which periodic polarity inversion enforces oxidation and reduction sequentially at the same electrode surface, eliminating the need for interelectrode ketone transport and enabling higher current densities and faster HIE under symmetric electrode configurations. The advantages of the AC approach may stem from a dual effect: first, the generation and subsequent reduction of the ketone intermediate occur at the same electrode during potential inversion, and second, while one-half-reaction proceeds at a given electrode, the reverse transformation occurs simultaneously at the counter electrode, thereby minimizing substrate diffusion constraints across the cell.

**Figure 5:**
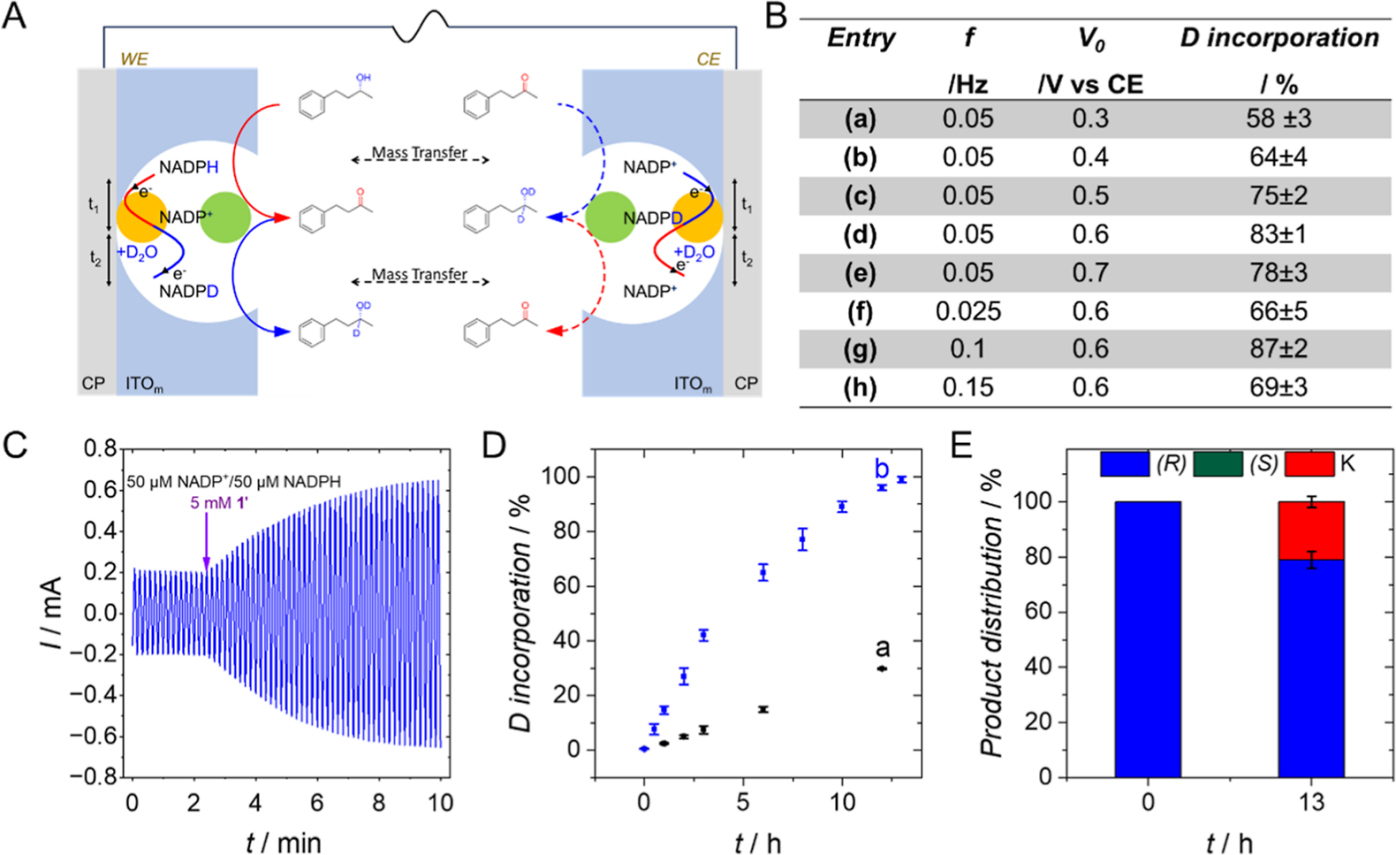
Optimization of AC electrolysis conditions and extension to preparative scale electroenzymatic HIE. (A) Schematic of AC paired electrolysis, where periodic potential oscillations alternately bias each electrode, enabling FNR-mediated NADP^+^/NADPD interconversion and sequential ADH-catalyzed alcohol oxidation and ketone reduction. (B) Optimization of AC amplitude and frequency in a 200 μL cell (10 mM (R)-4P2B, Tris-DCl, pD 9, 50 μM NADP^+^/50 μM NADPH; 0.9 nmol FNR and 0.3 nmol ADH LK per electrode), showing the resulting D incorporation after 1 h. (C–E) Preparative-scale electrolysis performed at 3 mL under 0.6 V vs CE and 0.1 Hz: (C) Representative AC chronoamperometric response; (D) time-dependent D incorporation measured by MS for AC electrolysis (blue, curve b) and DC electrolysis (black, curve a), conducted with identical catalyst loadings on both electrodes; and (E) product distribution after 13 h of AC electrolysis determined by chiral LC, confirming retention of (R)-selectivity with limited transient ketone formation. (Blue, **1**′; green, **2**′; red, **1**).

To optimize the electroenzymatic system, a 200 μL reactor containing 10 mM **1**′, 50 μM NADP^+^, and 50 μM NADPH in Tris-DCl was employed. Two ITO-modified electrodes were placed 1 mm apart, with one acting as the working and the other as the combined counter/reference electrode. This compact cell geometry confined the diffusion path and ensured rapid coupling between FNR-mediated cofactor regeneration and ADH LK-catalyzed substrate interconversion.

Optimization of AC electrolysis parameters focused first on the voltage amplitude (Figure 5B **entries a**–**e**). An amplitude window of 0.3–0.7 V was selected to span the onset of both oxidative and reductive waves (Figure 2D), enabling complete NADP^+^/NADPD cycling while avoiding parasitic processes. At 0.05 Hz, increasing the amplitude from 0.3 to 0.6 V led to a marked increase in HIE yield (≈58 → 83% after 1 h), consistent with enhanced FNR-mediated electron transfer and faster cofactor regeneration sustaining ADH LK turnover. Further increasing the amplitude to 0.7 V resulted in a moderate decline in efficiency, likely due to partial enzyme deactivation and competing side reactions. Accordingly, 0.6 V was identified as the optimal amplitude. On the other hand, the frequency also strongly influenced performance (**entries f**–**h**). At a fixed amplitude of 0.6 V, increasing the frequency from 0.025 to 0.1 Hz improved D incorporation (66 → 87%), whereas a further increase to 0.15 Hz reduced efficiency. This bell-shaped dependence reflects temporal matching between the applied oscillation and the coupled turnovers of FNR and ADH LK. At low frequencies, limited oscillation counts restrict overall turnover, whereas at high frequencies, polarity inversion outpaces cofactor cycling and substrate binding-release. An optimum near 0.1 Hz therefore maximizes net isotopic exchange. Under optimized AC conditions (0.6 V, 0.1 Hz, 1.5 h), the system achieved 96 ± 3% D-incorporation, exceeding that obtained under DC electrolysis (81%) at the same applied potential, even when a 4-fold higher catalyst loading was used at the counter electrode. This enhancement arises from alternating activation of both electrodes under AC operation, which maintains balanced NADP^+^/NADPD cycling and sustains continuous substrate turnover, thereby maximizing isotopic incorporation efficiency. To elucidate the impact of electrode configuration on redox symmetry and catalytic coupling, three AC electrolysis geometries were compared under identical conditions, revealing that a working-counter paired configuration—where oxidation and reduction alternate between electrodes each half-cycle—maximizes current response and HIE efficiency through dynamic interelectrode coupling (Supporting Information, Section 8).

Scaling the optimized AC strategy to a preparative volume (3 mL, Figure 5C–E) highlights the broader significance of alternating-potential electroenzymatic control. Stable, well-defined current oscillations are observed (Figure 5C), and upon addition of NADP^+^, NADPH, and 5 mM **1′**, the current amplitude rises sharply, reaching a maximum within 800 s. At the initial stage of AC electrolysis, electroneutrality is maintained through alternating cofactor oxidation and reduction at each electrode, with the complementary reaction occurring simultaneously at the counter electrode, prior to the establishment of the steady-state alcohol–ketone interconversion cycle. This rapid response reflects tight synchronization between electron flow and the enzymatic cascade, in which FNR sustains bidirectional cofactor cycling while ADH LK catalyzes oxidation and reduction within each potential half-cycle. In contrast, this current regime is not accessible under DC electrolysis with symmetric electrodes and requires several hours and strong geometric asymmetry to approach under DC conditions (Figure 4B). The kinetic profile of isotope incorporation further underscores the superiority of AC electrolysis (Figure 5D). Deuterium incorporation increases nearly linearly with time, reaching quantitative labeling after 12 h as validated by LC–MS and ^1^H NMR (Supporting Information, Section 13.5), corresponding to an approximately 4-fold enhancement relative to DC operation and an overall turnover frequency nearly five times higher. Furthermore, chiral LC analysis confirms exclusive formation of [^2^H] **1′** in addition to **1** (Figure 5E). Notably, the high HIE efficiency achieved under optimized AC conditions prompted an evaluation of NADP^+^/NADPH loadings, revealing that the use of only 10 μM NADP^+^ (instead of both cofactors) affords quasi-complete HIE with a the turnover number for NADP^+^ (TTN_NADP_) of 1723 ± 15 (Supporting Information, Sections 9 and 10).

### Substrate Scope of AC Paired Electrolysis

Following the establishment of efficient and enantioselective HIE under AC electrolysis, the methodology was extended to the investigation of substrate scope. The ADH LK-catalyzed H-D exchange displays a broad substrate scope across aromatic secondary alcohols (**A**_**1**_–**A**_**13**_; Figure 6) and reveals clear structure–reactivity relationships. Within the homologous series of unsubstituted benzylic alcohols—including **1**′ (**A**_**1**_), (*R*)-phenylpropan-2-ol (**A**_**3**_), and (*R*)-phenyl-2-ethanol (**A**_**4**_)—variation in alkyl chain length at the benzylic center has no measurable impact on HIE efficiency. All substrates undergo rapid, >95% D incorporation (≤2 h) with complete retention of configuration, and the coupled electroenzymatic cycle consistently favors the reduced alcohol, which accounts for >65% of the product distribution at completion.

**Figure 6:**
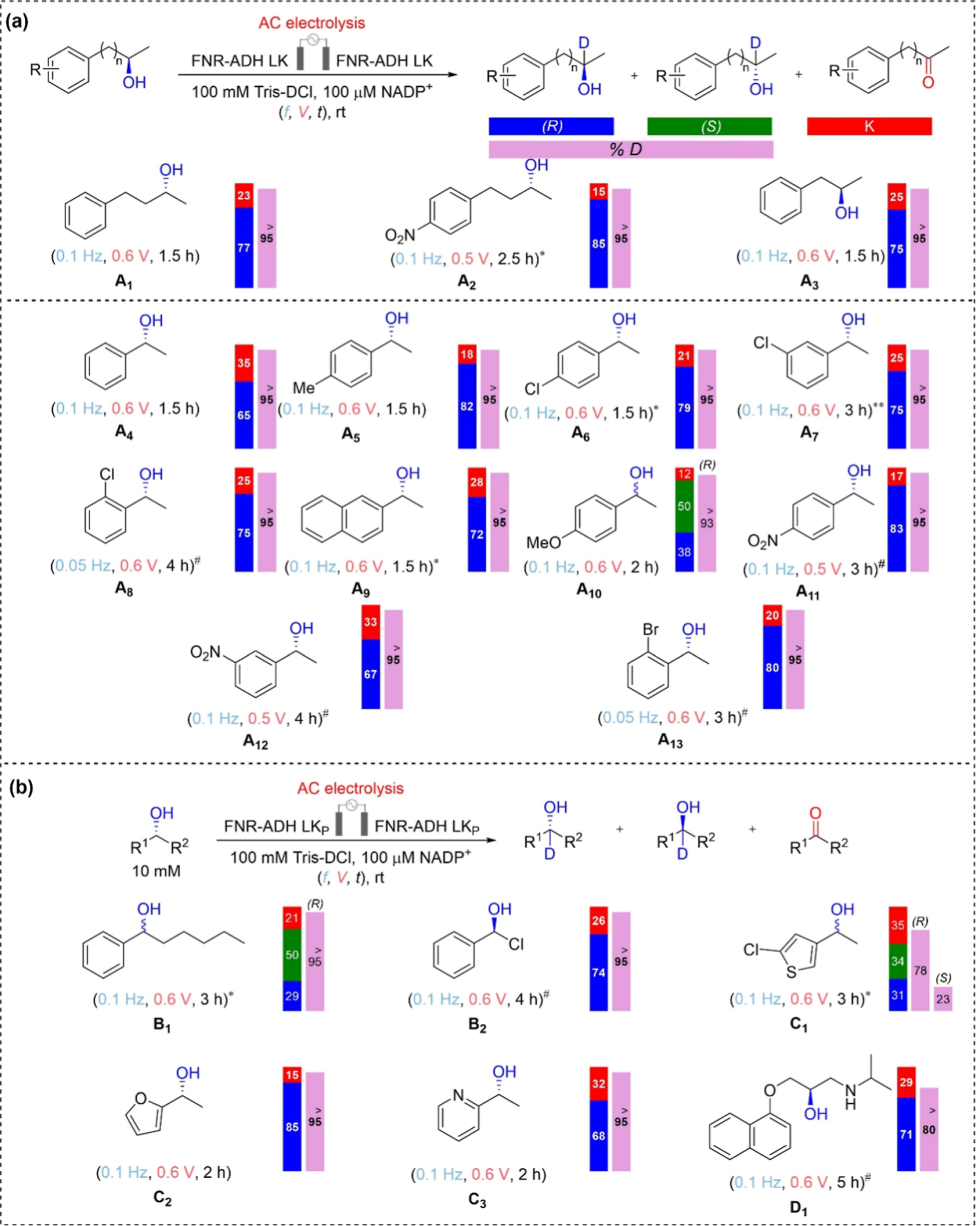
Substrate scope of AC-driven electroenzymatic H-D exchange catalyzed by FNR-ADH assemblies. (a) Electroenzymatic H-D exchange of aromatic secondary alcohols (**A**_**1**_–**A**_**13**_) catalyzed by FNR-ADH LK immobilized on ITO electrodes under AC electrolysis. (b) Extension of the platform to sterically demanding, heteroatom-containing, and drug-like secondary alcohols (**B**_**1**_–**B**_**3**_, **C**_**1**_–**C**_**3**_, **D**_**1**_) using ADH LK Prince (ADH LK_P_) under AC electrolysis. For all substrates, product distributions are reported as percentages of (R)-alcohol (blue bars) and (S)-alcohol (green bars). The ketone (red bars) was calculated from mass balance as the difference between the initial alcohol concentration and the total alcohol species remaining after reaction, assuming negligible evaporation and no formation of additional carbon-containing products. Overall D incorporation (% D) is shown in pink. The applied AC frequency (*f*), potential amplitude (*V*), and electrolysis time (*t*) are indicated for each substrate. Asterisks denote ADH loading relative to the standard amount: * 2-fold, ** 3-fold, and # 4-fold. General conditions: Reactions were performed in 100 mM Tris–DCl buffer (pD 9) containing 10 mM alcohol substrate (2.0 μmol in a total reaction volume of 200 μL) and 100 μM NADP^+^ (substrate-to-cofactor ratio 100:1, 1 mol % relative to substrate), with 5% DMSO (v/v) at room temperature. For substrates **A**_**1**_ and **C**_**1**_, the alcohol concentration was 20 mM (4.0 μmol), corresponding to a substrate-to-cofactor ratio of 200:1 (0.5 mol % NADP^+^). Reactions were conducted using ITO electrodes modified with 0.9 nmol FNR and 0.3 nmol ADH (standard amount). The total electrical input for the standard AC electrolysis conditions was determined by integration of the current–time response (Supporting Information, Section 13.6.3).

Substitution on the aromatic ring further delineates the influence of electronic and steric effects. Electron-donating substituents, such as methyl (**A**_**5**_) and methoxy (**A**_**10**_), exhibit HIE kinetics indistinguishable from the parent substrate **A**_**4**_, consistent with facilitated oxidation–reduction cycling. Across the substrate scope, reduced reactivity can be compensated through targeted system optimization, either by increasing the ADH LK loading or by decreasing the AC frequency (Supporting Information, Section 11). Increased steric bulk and π-extension in the naphthyl derivative **A**_**9**_ result in diminished turnover, requiring a 2-fold increase in ADH LK loading to achieve comparable D incorporation. Halogenated substrates (**A**_**6**_–**A**_**8**_, **A**_**13**_) reveal a progressive decrease in HIE efficiency with increasing electron withdrawal and steric congestion. The *para*-chloro derivative **A_6_** reaches >95% incorporation within 2 h using a 2-fold ADH LK loading, whereas the *meta*-chloro analogue **A**_**7**_ requires higher enzyme loading and extended electrolysis. For ortho-substituted substrates (**A**_**8**_, **A**_**13**_), steric hindrance near the benzylic center substantially impedes both oxidation and reduction steps, necessitating increased ADH LK loading, reduced AC frequency (0.05 Hz), and prolonged electrolysis to achieve quantitative labeling. Excessive ADH LK loading beyond this regime suppresses FNR-mediated cofactor regeneration, establishing waveform modulation as the preferred strategy for accommodating low reactivity substrates.

Nitro-substituted alcohols (**A_2_**, **A_12_**, **A_13_**) were operated at reduced AC amplitude (0.5 V) to suppress competing amination. Under these conditions, strong electron withdrawal consistently slows HIE, with increasing proximity of the nitro group to the benzylic center exacerbating the effect. *Para*-nitro substrates require elevated ADH LK loading to reach efficient incorporation, while closer substitution further reduces turnover and extends reaction times.

Overall, the substrate scope defines a coherent structure–reactivity landscape in which electron-rich systems sustain rapid HIE, whereas steric encumbrance and electron withdrawal progressively attenuate catalytic turnover. Efficient labeling of more demanding substrates is enabled by balancing enzyme loading with temporal control of redox cycling, underscoring AC electrolysis as a tunable platform for stereoselective HIE across structurally diverse aromatic alcohols.

While ADH LK efficiently catalyzes H-D exchange for simple and moderately substituted aromatic secondary alcohols, substrates with increased steric demand or extended aromatic frameworks exhibit diminished turnover, consistent with steric constraints imposed by the native active site. To expand the accessible substrate space under AC electroenzymatic conditions, we employed an engineered Lactobacillus kefir alcohol dehydrogenase variant, ADH LK Prince (ADH LK_P_), which was developed to accommodate sterically demanding “bulky–bulky” carbonyl substrates through active-site remodeling.^45^ As a benchmark, **1**′ was examined under identical AC electrolysis conditions to enable direct comparison with ADH LK. For this substrate, ADH LK_P_ exhibits lower intrinsic turnover frequencies for oxidation and reduction (18.4 ± 1.1 and 16.9 ± 2.9 s^–1^, respectively) relative to ADH LK, resulting in extended electrolysis times (3 h vs 1.5 h) to achieve complete enantioselective HIE.

In contrast, substrate-dependent advantages emerge for sterically encumbered alcohols. Extension of the benzylic substituent (**B**_**1**_) or introduction of proximal steric and electronic constraints (**B**_**2**_) shifts the balance in favor of ADH LK_P_, where increased enzyme loading restores efficient redox cycling and enables near-quantitative D incorporation. For heteroatom-containing substrates, oxygen- and nitrogen-substituted alcohols (**C**_**2**_, **C**_**3**_) undergo complete, stereoretentive HIE within 2 h, whereas sulfur-containing substrates (**C**_**1**_) exhibit reduced turnover, incomplete labeling, and partial erosion of enantioselectivity (er_(R/S)_ ≈ 78:23), even with higher catalysts loading. Importantly, the expanded active-site architecture enables labeling of structurally complex, drug-like substrates, as exemplified by (*R*)-propranolol (**D**_**1**_), which achieves 83% D incorporation with retention of configuration under AC electrolysis. In all AC electrolysis experiments across the substrate scope, the amount of ketone was found to range between approximately 15% and 35%. The observed variation in ketone levels likely reflects substrate-dependent differences in enzyme-substrate affinity and catalytic efficiency within the reversible alcohol/ketone cycle. Although the reactions were conducted under identical buffered conditions, it is well established that pH and temperature can influence alcohol dehydrogenase activity and reaction kinetics;^46^ therefore, the accumulation of ketone observed for certain substrates may also be influenced by the specific pH and temperature conditions employed, which can affect the balance between oxidation and reduction catalyzed by ADH. To further corroborate the D incorporation determined by LC–MS, selected substrates from the scope were analyzed by ^1^H NMR, confirming site-specific D incorporation (93% D) (Supporting Information, Section 13.6). Notably, a higher transient accumulation of ketone intermediates was observed in larger–volume reactions (3 mL) used for NMR analysis compared to microscale reactions (200 μL) used for routine mass spectrometric monitoring, consistent with volume-dependent mass transport effects under AC electrolysis.^47,48^ The NMR experiments were performed to validate α-specific D incorporation, whereas MS served as the primary analytical method in this electrochemical system; further optimization of electrode surface properties and operating conditions, including substrate-dependent reactivity with ADH, is expected to enhance system performance.

### Application of DC Electrolysis with Enantioselectivity Inversion and Deracemization

While AC electrolysis provides a highly effective platform for enantio-retentive H-D exchange, its stereochemical outcome is intrinsically fixed because oxidation and reduction proceed sequentially through the same enzyme at a given interface. To extend electrochemical control beyond retention and enable directional stereochemical editing, we therefore turned to DC electrolysis, in which oxidative and reductive half-reactions are spatially separated between the anode and cathode.

ADH LK preferentially oxidizes and reduces the (*R*)-alcohol, whereas TeSW110A (ADH *Te*) displays complementary selectivity for the (S)-alcohol in both directions.^49–51^ *Te*SW110A was selected as the *S*-selective partner for DC electrolysis due to its substantially higher turnover frequencies for oxidation and reduction (27 ± 3 and 45.3 ± 2.2 s^–1^, respectively) compared to the *Te*SW110 V variant. The S-selectivity of ADH *TE* was independently validated under AC electrolysis, which afforded quantitative HIE of (*S*)-4-phenyl-2-butanol (**2**′) with complete stereoretention (Supporting Information, Section 13.8). DC electrolysis enables stereochemical control by assigning oxidation and reduction to distinct enzymatic microenvironments (Figure 7). In **configuration A**, the anode was functionalized with FNR-ADH LK and the cathode with FNR-ADH *TE*, directing oxidation of **1**′ followed by reductive deuteration under opposite stereocontrol to afford predominantly [^2^H] **2**′ after 3 h at 0.6 V vs CE (66 ± 7% (*S*), er_(*S*/*R*)_ ≈ 6:1, 100% D). Reversing the enzyme identities (**configuration B**) inverted the stereochemical outcome, yielding predominantly [^2^H] **1**′ from **2**′ (63 ± 9% (*R*), er_(*R*/*S*)_ ≈ 9:1). In both cases, minor retention of the starting enantiomer is attributed to partial anode desorption rather than reductive crossover.

**Figure 7:**
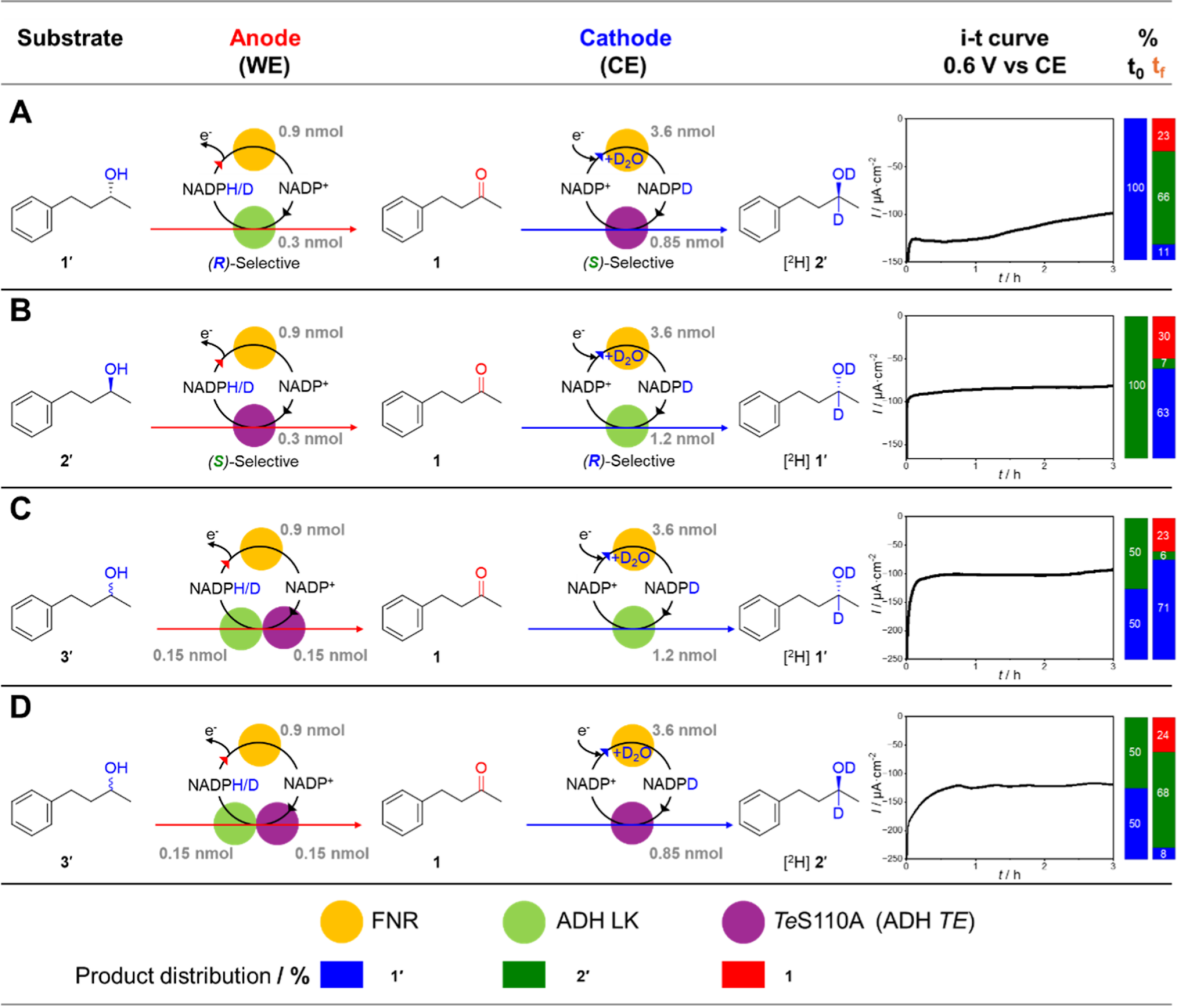
Stereochemical control via DC paired electrolysis by spatial separation of oxidative and reductive electroenzymatic steps. Schematic representations of four DC electrolysis configurations (A–D) illustrating spatial separation of oxidative and reductive electroenzymatic steps through selective placement of FNR and stereocomplementary ADHs at the anode (WE) and cathode (CE). Bulk electrolysis was performed at 0.6 V vs CE for 3 h. Representative current–time (*i*–*t*) traces are shown for each configuration. (A) **1**′ with FNR-ADH LK at the anode and FNR-ADH *TE* at the cathode, affording enantio-inverted [^2^H] **2**′. (B) **2**′ with FNR-ADH *TE* at the anode and FNR-ADH LK at the cathode, affording enantio-inverted [^2^H] **1**′. (C) 4-phenyl-2-butanol (**3**′) with a dual-ADH (LK + *TE*) anode and ADH LK at the cathode, enabling deracemization toward [^2^H] **1**’. (D) **3**′ with a dual-ADH (LK + *TE*) anode and ADH *TE* at the cathode, enabling deracemization toward [^2^H] **2**′. FNR loadings were 0.9 nmol at the anode and 3.6 nmol at the cathode; ADH loadings are indicated in the schematics. Product distributions are reported as percentages of **1**′ (blue), **2**′ (green), and 1 (red). Reaction conditions: 100 mM Tris-DCl buffer (pD 9) in D_2_O (200 μL) containing NADP^+^ (50 μM) and NADPH (50 μM), room temperature.

Building on this inversion capability, **configuration C** translates electrode control into electroenzymatic deracemization. Using racemic **3**′, both stereocomplementary ADHs were coimmobilized at the anode to selectively oxidize each enantiomer to a common ketone intermediate, while the cathode contained only ADH LK. After 3 h at 0.6 V vs CE, full D incorporation was obtained and the product distribution favored [^2^H] **1**′ (71%, er_(R/S)_ ≈ 12:1). Substitution of the cathodic enzyme with ADH *TE* (**configuration D**) redirected the deracemization outcome, yielding predominantly [^2^H] **2**′ (68 ± 4%, er_(R/S)_ ≈ 8.5:1). Furthermore, representative DC electrolysis products were examined by MS and ^1^H NMR spectroscopy, confirming high levels of site-specific D incorporation at the stereogenic center (>95% D) (Supporting Information, Section 13.7). Collectively, these configurations demonstrate that DC electrolysis not only enables precise enantio-inversion of a chiral alcohol but also affords complete stereochemical funneling of a racemic mixture, with quantitative isotopic incorporation and stereochemical outcomes governed solely by the reductive ADH embedded at the cathode.

## Conclusions

In summary, we have developed a versatile bioelectrochemical platform that integrates bidirectional, electrode-driven cofactor regeneration with enzyme catalysis to achieve highly efficient, stereoselective H-D exchange at chiral alcohols. The system couples a wired FNR enzyme for continuous NADP^+^/NADPD interconversion with stereospecific alcohol dehydrogenases to form a reversible electroenzymatic cycle. Under mild aqueous conditions, this approach delivers near-quantitative D incorporation (typically >95%) and complete enantiopurity (>99% ee) in labeled products. Crucially, by selecting the mode of electrolysis, we can “program” the stereochemical outcome: AC electrolysis realizes rapid, stereoretentive HIE using a single ADH, whereas DC paired electrolysis permits stereochemical editing (retention vs inversion) or deracemization via different ADH placements. This dual-mode control is unique: we demonstrate 100% retention, efficient inversion, and full deuterative deracemization of racemic mixtures, all within the same cofactor-enzyme framework. Our results thus overcome longstanding limitations in asymmetric isotope labeling by eliminating sacrificial deuterated substrates and harnessing clean electrical energy to drive the reaction cycle.

Looking forward, this work opens several exciting avenues. First, the general concept of redox-programmable biocatalysis can be extended to other isotopic labels (e.g., tritium) and to a wider range of enzyme classes (ketoreductases, imine reductases, etc.), enabling new modes of isotope labeling. The high TTNs achieved here (e.g., ∼1700 for NADP^+^ with only 10 μM) suggest practical scalability and sustainability. Second, the oscillatory AC approach hints at novel reactor designs: for example, integrating periodic electrochemical bias with fuel cell architectures could couple enantioselective HIE to energy production (Supporting Information, Section 12). Finally, in the context of pharmaceutical synthesis, our method provides a general platform for late-stage deuteration of complex drug-like molecules under biocompatible conditions. We anticipate that further enzyme engineering (to broaden substrate scope) and electrochemical optimization (to improve rates) will make this redox-electroenzymatic strategy a powerful tool in both synthetic chemistry and biomanufacturing.

## Supplementary Material



## Notes

It contains experimental procedures; materials and methods; plasmid construction and protein expression and purification; enzymatic kinetic assays; electrochemical methods and electrode fabrication; preparation of deuterated buffers; LC–MS, a,chiral LC methods, and ^1^H NMR; additional electrochemical data; electrochemical stability and kinetic analyses of FNR- and FNR–ADH-modified electrodes; mechanistic schemes; AC and DC electrolysis comparisons; fuel-driven electroenzymatic H–D exchange experiments; preparative-scale studies; substrate-scope investigations; and supplementary figures, tables, and spectra supporting the results discussed in the main text (PDF)
