# Supplementary material for: Employing AC and DC Electrolysis to Modulate Electroenzymatic Pathways for Efficient and Stereoselective H‑D Exchange
Source: J Am Chem Soc. 2026 May 28;148(22):22641–52. doi: 10.1021/jacs.6c02284 (PMC13266969; doi:10.1021/jacs.6c02284)
Supplement: Supplementary file 1 [file ja6c02284_si_001.pdf]

Supporting Information

Employing AC and DC Electrolysis to Modulate Electroenzymatic Pathways for Efficient and Stereoselective H–D Exchange

Wassim El Housseini, §[a] Rokas Gerulskis, §[b] Nibedita Behera, [b] Huaijun Guan, [a] Rohit G. Jadhav, [a] Zachary A. Nguyen, [b] Egor Baiarashov, [a] Michael Pence, [a] Vamshi Krishna Kamaja,[a] Trevor Larkin, [a] Long Luo, * [b] Shelley D. Minteer*[a]

[a]Kummer Institute Center for Resource Sustainability, Missouri University of Science and Technology, Rolla, Missouri 65409, USA

[b]Department of Chemistry, University of Utah, Salt Lake City, UT, 84112 USA

*long.luo@utah.edu

*shelley.minteer@mst.edu

§ : these authors contributed equally to this work

Contents

1 Experimental section 3

2 Effect of pH on electroenzymatic $NADP^+$/NADPH interconversion 14

3 Electrochemical stability of FNR@ITO_m electrodes 16

4 Bidirectional electroenzymatic $NADP^+$/NADPH turnover by FNR@ITO 18

5 Cyclic voltametric evidence for FNR–ADH LK-mediated alcohol oxidation and ketone reduction 19

6 Steady-state electroenzymatic kinetics of alcohol–ketone interconversion 21

7 Electrochemical control of enantioselective alcohol oxidation by FNR–ADH LK@ITO_m 22

8 Influence of AC electrolysis geometry on paired electroenzymatic HIE efficiency 25

9 Influence of electrolysis regime on cofactor cycling efficiency in HIE 27

10 Efficient electroenzymatic HIE under AC operation at low cofactor concentrations 28

11 Generality of AC-driven electroenzymatic HIE 31

12 Fuel-Driven, waveform-controlled electroenzymatic HIE 35

13 Appendix 39

14 References 113

1 Experimental section

1.1 Chemicals

All chemicals were of analytical grade or higher and were used as received unless otherwise stated. Trizma® base (($^2H_5$)-Tris), (3-aminopropyl)triethoxysilane (APTES), $NADP^+$ (β-nicotinamide adenine dinucleotide phosphate, oxidized form), NADPH, and other biochemical reagents were purchased from Sigma-Aldrich. Deuterium oxide (^2H_2O, ≥99.9 atom % D) and deuterium chloride (^2HCl, 3.0 M in ^2H_2O) were also obtained from Sigma-Aldrich.

Organic substrates, including benzylic alcohols and ketones, were purchased from commercial suppliers (Sigma-Aldrich, TCI Chemicals, or Alfa Aesar) with stated purities ≥98 % and were used without further purification.

Indium tin oxide (ITO) solution (≥99.5% trace metals basis, 18 nm particle size) was obtained from Sigma-Aldrich, and carbon paper substrates were purchased from Fuel Cell Store. All aqueous solutions were prepared using D_2O or ultrapure water (18.2 MΩ·cm), as specified for each experiment. High-purity gases (N_2, Ar; ≥99.9%) were supplied by commercial gas vendors. Unless otherwise noted, all materials were used as received, and no additional purification steps were performed.

1.2 Plasmid Preparation

The gene coding for the mature form of FNR from *Chlamydomonas reinhardtii* (UniProt A8J6Y8, amino acids 27 to 346),[1] ADH LK from *Lactobacillus Kefiri* (UniProt Q6WVP7) and ADH LK Prince (mutant E157F-F169L-Y202C),[2] and ADH *TE* from *Thermoanaerobater ethanolicus* (UniProt P77990, mutant W110V)[3] were synthesized and ligated into plasmid pET28a (Nco1, Xho1) by Genscript. The ADH LK and ADH *TE* sequences were modified to produce an N-terminal HHHHHHGSSG sequence. An attempt was made to purify ADH LK with a C-terminal GSSGHHHHH tag, because structure analysis with Terminator suggested a similar modification risk for either terminus,[4] but the N-terminal version demonstrated superior yield and purity.

1.3 Expression of proteins

All plasmids were transformed into E. coli strain BL21(DE3)PlysE. A starter culture in LB media (10 mL, 20 g/L Luria-Bertani dry mix, 100 μg/mL kanamycin, incubated at 30°C, 120 rpm, overnight) was used to inoculate 1 L of LB media (as above, buffered to pH 7 with 100 mM sodium phosphate), and the culture was grown at 37°C to an OD600 of 0.5. FNR was induced with 0.1 mM IPTG (Isopropyl β-D-1-thiogalactopyranoside) and grown for 4h at 37°C. ADH *TE* was induced with 0.1 mM IPTG and grown for 20h at 37°C. ADH LK was induced with 1.3 mM IPTG and grown for 20h at 30°C. Cells were pelleted (7k x g, 10 minutes), frozen in liquid N2, and stored at -80°C until purification.

1.4 Purification of FNR

Cell pellet was suspended in lysis buffer (Tris-HCl 40 mM, NaCl 20 mM, Complete EDTA-free protease inhibitor 1 tablet/50 mL, pH 8) and lysed by sonication (30 sec on, 30 sec off, 10 minutes on-time, 80% power) using a probe sonicator (Fisherbrand Model 505, Qsonica cl334, 1/2" probe), in a stainless steel beaker with continuous stirring in an ice bath. This lysate was then passed twice through a microfluidizer (Microfluidics M-110P, 20 kPSI). Supernatant was harvested by centrifugation (9kxg, 50 minutes, 4°C). The supernatant was loaded on a Q-sepharose column (200 mL bed-volume) pre-equilibrated with loading buffer (as lysis without protease inhibitor) and eluted with a gradient of elution buffer (as loading buffer, with 500 mM NaCl) in the course of 400 mL. Fractions containing FNR were determined by SDS-PAGE and the protein was concentrated using a 30K MWCO Amicon centrifugal filter (Millipore). Concentrated protein was divided into 30 uL fractions, flash frozen in liquid N_2, and stored at -80°C.

1.5 Purification of ADH LK variants and ADH *TE*

Cell pellet was suspended in lysis buffer (Tris-HCl 40 mM, imidazole 10 mM, KCl 500 mM, Complete EDTA-free protease inhibitor 1 tablet/50 mL, pH 8. ADH LK lysis buffer additionally contained $MgCl_2$ 1 mM), then sonicated, microfluidized, and centrifuged as FNR. The supernatant was loaded on a Ni-column (HisPrep FF 16/10, Cytiva) pre-equilibrated with loading buffer (as lysis without protease inhibitor) and eluted with a gradient of elution buffer (as loading buffer, with 500 mM Imidazole) in the course of 450 mL. Fractions containing ADH LK were determined by SDS-PAGE and the protein was concentrated using a 10K MWCO Amicon centrifugal filter (Millipore) for ADH LK, or 30K MWCO for ADH *TE*. Concentrated protein was loaded onto a desalting column (HiPrep Sephadex G-25, Cytiva) pre-equilibrated with storage buffer (as lysis without imidazole or protease inhibitor) to remove imidazole, then concentrated again.

Concentrated protein was divided into 30 uL fractions, flash frozen in liquid N_2, and stored at -80°C.

All protein samples were buffer-exchanged into 40 mM Tris–DCl prior to analysis, following standard practice for^{1}H NMR experiments.

1.6 Solution-based kinetic assays for alcohol dehydrogenases

Solution assays to quantify ADH-catalyzed alcohol oxidation and ketone reduction were performed using UV–vis spectroscopy in 96-well plates. Alcohol oxidation assays employed either (*S*)-4-phenyl-2-butanol (**2′**) or (*R*)-4-phenyl-2-butanol (**1′**)as substrates at pH 9.0, whereas ketone reduction assays used 4-phenyl-2-butanone as the substrate. Substrate concentrations were varied from 0.09 to 10 mM. Enzymatic activity was monitored in triplicate by following the production (alcohol oxidation) or consumption (ketone reduction) of NADPH at 340 nm over time. Initial reaction rates were determined from the linear region of the corresponding increase or decrease in absorbance.

Kinetic parameters were obtained by nonlinear regression using the Michaelis–Menten model, where v is the reaction rate, K□ is the Michaelis–Menten constant, and [S] represents the substrate concentration (**2′**, **1′**, or 4-phenyl-2-butanone (**1**)).

All assays were conducted at 25 ± 2 °C in a total reaction volume of 200 µL. Alcohol oxidation and ketone reduction reactions were performed in 100 mM TAPS buffer (pH 9.0) containing 5 mM $MgCl_2$, using 200 µM $NADP^+$ for alcohol oxidation and 200 µM NADPH for ketone reduction.

1.7 Electrochemical measurement

Electrochemical measurements were performed using a CH Instruments 760F potentiostat in a conventional three-electrode configuration. The working electrode was as specified for each experiment, a platinum mesh served as the counter electrode, and a saturated calomel electrode (SCE) was used as the reference electrode. All potentials reported in the manuscript versus the standard hydrogen electrode (SHE) were converted from SCE using the relationship:

$$E(SHE) = E(SCE) + 0.241 \text{ V at } 298 \text{ K}$$

CV experiments were conducted using a low-volume undivided electrochemical cell. Single-electrolysis experiments were performed in a 5 mL H-cell, with the working and counter compartments separated by Nafion 117 membrane.

Alternating-current (AC) and direct-current (DC) paired electrolysis experiments were carried out in a 1.5 mL Eppendorf tube–based electrochemical setup, configured to accommodate solution volumes as low as 200 μL.

1.8 Preparation of ($^2H_5$)-Tris–^{2}HCl (Tris-DCl) buffer in D_2O

The ($^2H_5$)-Tris–^{2}HCl (Tris-DCl) buffer was prepared by dissolving Trizma® base in D_2O, followed by evaporation to dryness. This dissolution–evaporation procedure was repeated twice to promote exhaustive isotopic exchange of labile protons. The residue was subsequently redissolved in D_2O, and the pD (p^{2}H) was adjusted to 9.0 using small aliquots of 3.0 M ^{2}HCl (DCl). Because standard glass electrodes are calibrated in H_2O and underestimate acidity in D_2O, reported pD values correspond to pH meter readings corrected according to pD = pH + 0.4. Prior to use, the buffer was deoxygenated by sparging with dry Ar or N_2.

1.9 FNR / FNR-ADH@ITO$_m$ electrode preparation

Carbon paper electrodes were cut and prepared as previously reported.[5] ITO–modified carbon paper electrodes were then fabricated by drop-casting 4 µL of an ITO nanoparticles solution onto the carbon paper substrates, followed by drying at 80 °C for 20 min to ensure solvent evaporation and adhesion of the ITO layer. The ITO-coated electrodes were subsequently hydroxylated by immersion in 1.0 M NaOH for 30 min at room temperature to generate surface hydroxyl groups. After treatment, the electrodes were rinsed thoroughly with ultrapure water until neutral pH was reached and dried under a stream of N_2.

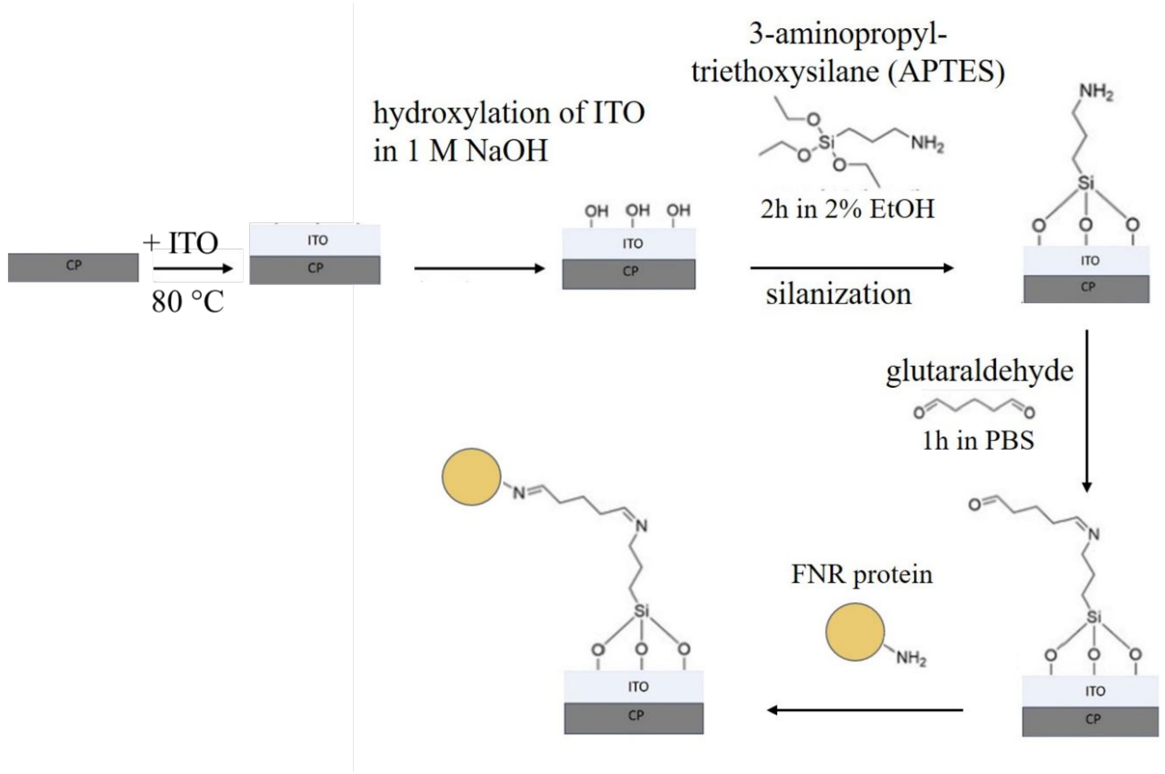

Figure S1. Preparation of enzyme-functionalized ITO-modified carbon paper electrodes via ITO deposition, surface hydroxylation, APTES silinization, glutaraldehyde activation, and covalent immobilization of FNR.

Silanization was performed by incubating the hydroxylated ITO electrodes in a 2% (v/v) solution APTES in ethanol for 2 h at room temperature. The electrodes were then rinsed extensively with

ethanol to remove excess silane and allowed to cure under ambient conditions to promote siloxane network formation. The amine-functionalized surfaces were activated by immersion in an aqueous glutaraldehyde solution prepared in phosphate-buffered saline (PBS) for 1 h at room temperature. Following activation, the electrodes were rinsed with PBS to remove unreacted glutaraldehyde.

FNR was immobilized by incubating the activated electrodes in an FNR-containing buffer solution, enabling covalent attachment via Schiff base formation between surface aldehyde groups and lysine residues on the protein. After immobilization, the electrodes were gently rinsed with buffer to remove non-specifically adsorbed enzyme and stored in buffer at 4 °C until use. Electrodes co-functionalized with FNR and ADHs were prepared following the same procedure, using mixed enzyme solutions as specified for each experiment.

1.10 LC-MS analysis

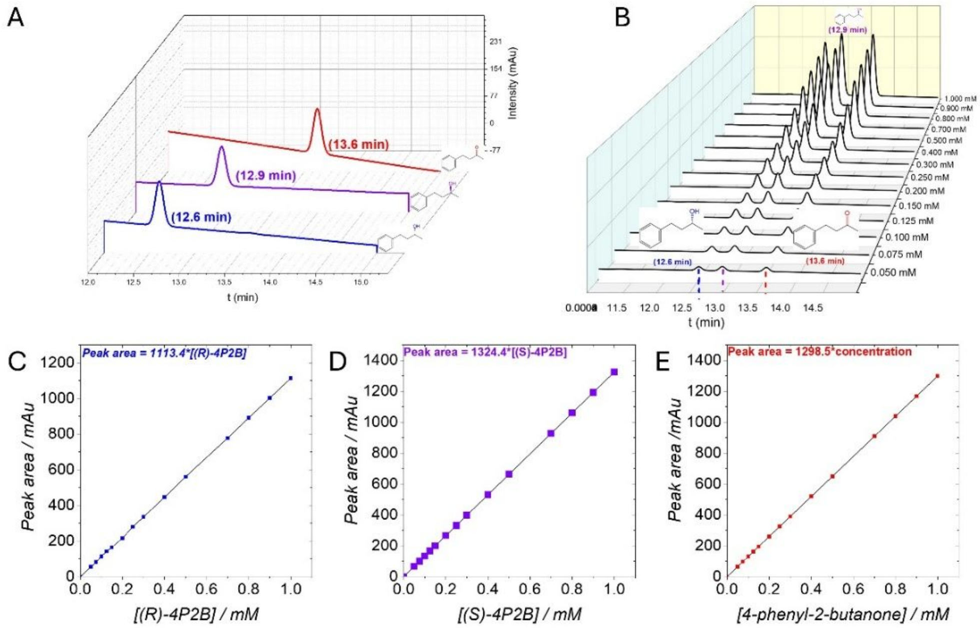

Figure S2. LC–MS quantitative analysis and calibration of alcohol and ketone standards. (A) Overlay of extracted chromatograms showing the separation of **1′** (blue, t_e = 12.6 min), **2′** (purple, t_e = 12.9 min), and **1** (red, t_e =

13.6 min) under the optimized LC–MS conditions. (B) Stacked chromatograms obtained for increasing concentrations of authentic standards (0.05–1.0 mM), illustrating concentration-dependent signal intensity and retention time reproducibility. (C–E) External calibration curves constructed from integrated DAD peak areas at 210 nm for (C) **1′**, (D) **2′**, and (E) **1**.

LC–MS analyses were performed to quantify 4-phenyl-2-butanol and **1**. Prior to analysis, samples were prepared by diluting 100 µL of the stock solution with 900 µL of the initial mobile phase consisting of 75% H_2O and 25% acetonitrile (v/v). All mobile phases contain 0.1% formic acid. Prepared samples were mixed thoroughly prior to injection.

Chromatographic separations were carried out on a reversed-phase LC column (CHIRALPAK® AD-H 4.6x250mm, 5 micron) maintained at a column temperature of 40 °C. The injection volume was 4 µL, and the flow rate was set to 0.40 $mL \cdot min^{-1}$.

The LC method employed a two-step isocratic–step gradient program. From 0 to 12 min, the mobile phase composition was held at 75% H_2O and 25% acetonitrile (v/v). From 12 to 16 min, the composition was switched to 75% acetonitrile and 25% H_2O (v/v) to elute more strongly retained components and regenerate the column prior to the next injection.

Detection was performed using a diode array detector (DAD) at 210 nm, with mass spectrometric detection used for compound confirmation. Under these conditions, the retention times were approximately 12.6 min for **1′**, 12.9 min for **2′**, and 13.6 min for **1**.

- **LC–MS analysis of samples before and after reaction for all substrates.**

An aliquot of the reaction mixture (100 µL) was diluted tenfold with mobile phase (900 µL) to a final volume of 1.0 mL prior to LC–MS analysis.

The extent of HIE was quantified by MS analysis of the reaction mixtures. Deuterium incorporation was determined from the relative abundances (%) of the unlabeled (M) and monodeuterated (M+1) isotopologues of the molecular ion or a diagnostic fragment that retains the labeled carbon center. Isotopologue abundances were extracted from the same chromatographic peak and the percentage of HIE was calculated according to:

$$\% \, HIE \equiv \% \, D = \frac{\text{Abundance}_{\,(\text{M+1})}}{\text{Abundance}_{\,(\text{M+1})} + \text{Abundance}_{\,(\text{M})}} \times 100 \qquad (Equation\, S1)$$

1.11 ^{1}H NMR analysis

Hydrogen isotope exchange (HIE) reactions were performed in 100 mM Tris-d_{11}–DCl buffer (pD 9) containing 5 mM $MgCl_2$ and 5% DMSO-d_6**.** Aliquots (600 µL to 1.0 mL) were withdrawn directly from the reaction mixture and transferred to NMR tubes for analysis without further workup. For ketone reduction experiments conducted in media containing varying percentages of D_2O, reaction mixtures were extracted three to four timeswith methyl tert-butyl ether (MTBE)**.** Combined organic phaseswere collected, solvent was removed under a gentle stream of argon, and the residue was redissolved in 100 mM Tris-d11–DCl buffer (pD 9) supplemented with 5% DMSO-d_6 prior to analysis. ^{1}H NMR spectra were recorded on a 400 MHz Bruker spectrometer at room temperature. Deuterium incorporation was determined from the decrease in the ^{1}H NMR signal corresponding to the proton at the stereogenic center relative to the starting material.

1.12 Fuel cell automated gas switching device: Hardware and control architecture

A device for automatically controlling gas flow was constructed using an Arduino Uno and two normally-closed 12 V DC solenoid valves. The device was controlled by a Python program, sending commands to the Arduino by serial communication. The Arduino then controlled an IRF520 MOSFET to switch 12 V power to the solenoid valves. A schematic of the device is shown in **Figure S3** and the bill of materials can be found in **Table S1**.

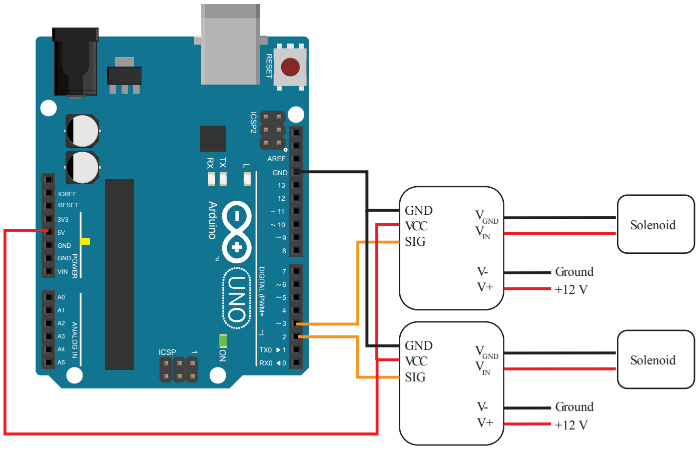

Figure S3. Schematic of the connections between the Arduino, the valve driver board, and the solenoid valves.

Table S1. Bill of materials for the gas switching device.

Item	Quantity	Price / $	Link
Arduino	1	13.99	https://www.amazon.com/ELEGOO-Controller-ATmega328P-Compatible-Arduino/dp/B0B6VV7MS7

Arduino Terminal	1	18.99	https://www.amazon.com/Electronics-Salon-Arduino-Terminal-Breakout-Module/dp/B07HF2DD7T/
Valve	2	8.49	https://www.amazon.com/Beduan-Normally-Closed-Electric-Solenoid/dp/B07N2DZ5FP
Valve driver	1	6.99	https://www.amazon.com/HiLetgo-IRF520-MOSFET-Arduino-Raspberry/dp/B01I1J14MO
Power Supply	1	6.99	https://www.amazon.com/Supply-Universal-Adapter-AC120V-Transformer/dp/B0C22Z8DPL

2 Effect of pH on electroenzymatic $NADP^+$/NADPH interconversion

CV and CA were employed to evaluate the pH dependence of electroenzymatic $NADP^+$ reduction at FNR-modified electrodes in 100 mM Tris–HCl buffer (**Figure S4**). In the absence of $NADP^+$, the voltammetric response is dominated by non-turnover FNR redox features, which show only modest variations across the pH range investigated (pH 7–9; **Figure S4A–C**). Upon addition of 3 mM $NADP^+$, a clear catalytic response is observed at all pH values, confirming effective electronic coupling between the electrode and FNR for cofactor reduction and oxidation in the reverse scan. Notably, the magnitude of the catalytic current increases systematically with pH, with the largest enhancement observed at pH 9, indicating more favorable reaction kinetics under alkaline conditions.

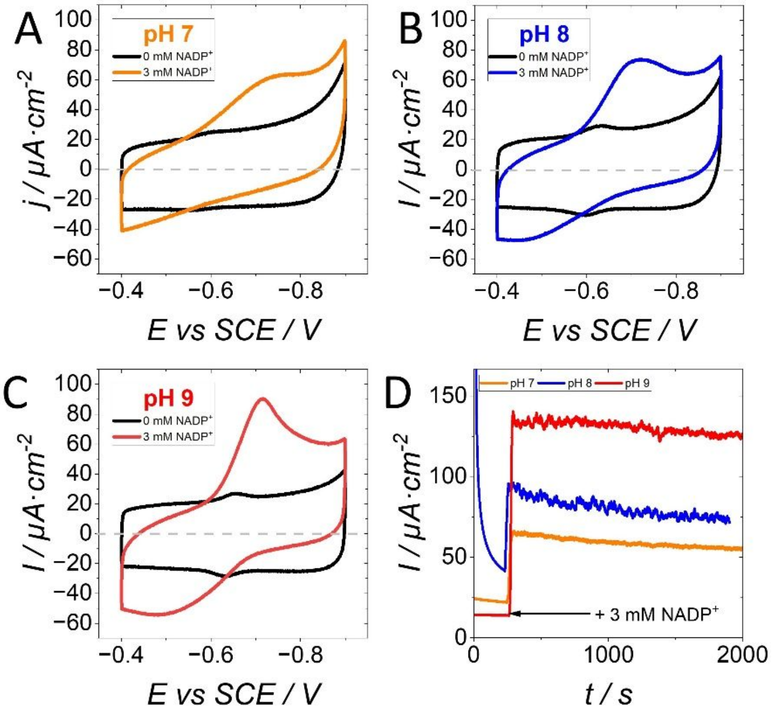

Figure S4. pH-dependent electroenzymatic NADP⁺ reduction at FNR@CP-ITO$_m$. (A–C) Cyclic voltammograms recorded at a scan rate of 2 mV·s⁻¹ at pH 7 (A), pH 8 (B), and pH 9 (C) in 100 mM Tris–HCl buffer, in the absence (black traces) and presence (colored traces) of 3 mM NADP⁺. (D) Chronoamperometric responses recorded at a constant potential of –0.6 V vs SHE in 100 mM Tris–HCl buffer following the addition of 3 mM NADP⁺ at pH 7 (orange), pH 8 (blue), and pH 9 (red).

Chronoamperometric measurements recorded at –0.6 V vs SHE further emphasizes the influence of pH on catalytic performance (**Figure S4D**). Following the addition of NADP⁺, the steady-state current density increases in the order pH 7 < pH 8 < pH 9, with pH 9 exhibiting both the highest catalytic current and the most stable response over prolonged electrolysis. Based on the combined voltammetric and chronoamperometric data presented in **Figure S4**, pH 9 was selected as the most suitable operating condition for subsequent electroenzymatic studies.

3 Electrochemical stability of FNR@ITO$_m$ electrodes

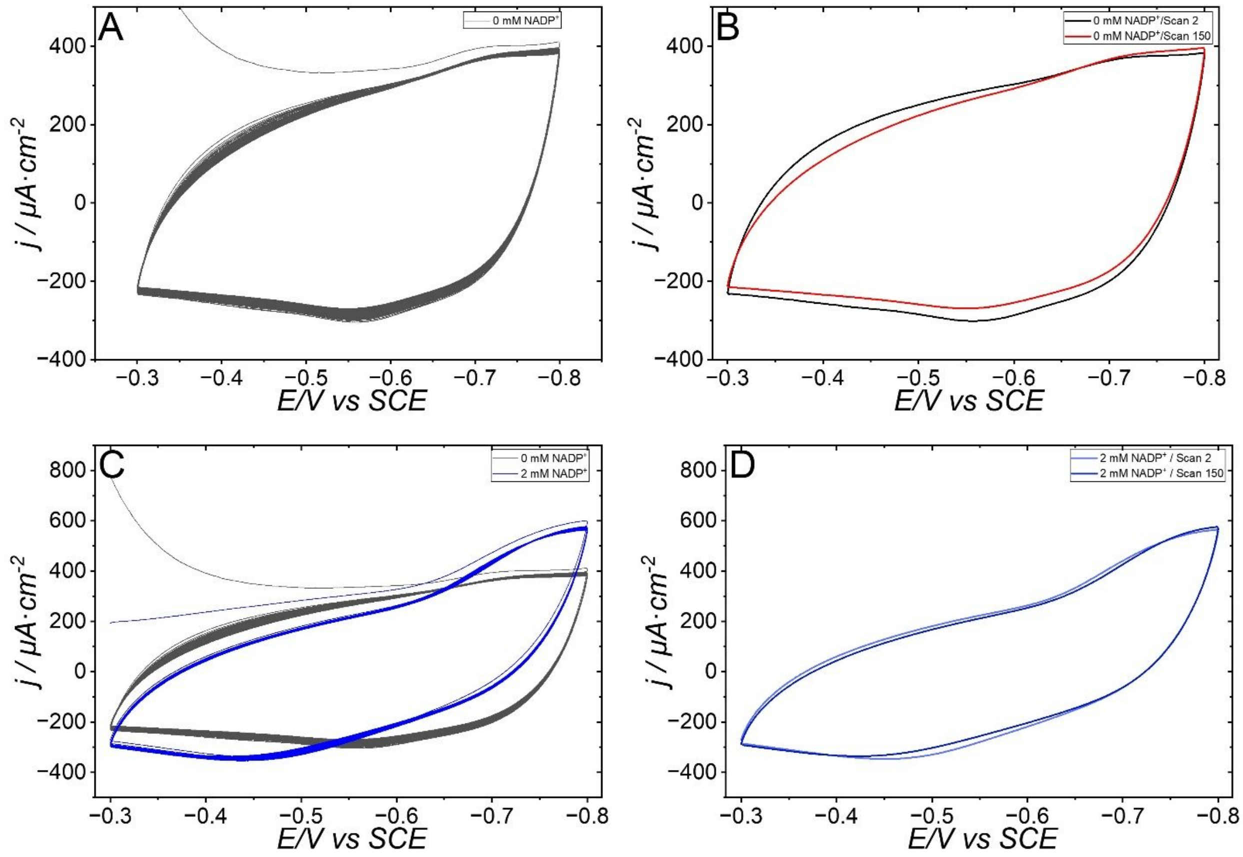

Figure S5. Electrochemical stability of FNR@ITO bioelectrodes at pH 9. (A) Consecutive CVs of FNR@ITO recorded in 100 mM Tris-HCl buffer (pH 9) under non-turnover conditions at a scan rate of 10 mV·s^{-1}. (B) Overlay of the 2nd and 150th CVs shown in panel A, demonstrating ≥95% current retention after ~4.2 h of continuous cycling. (C) CVs recorded in the presence of 2 mM NADP$^+$, showing the catalytic current associated with electroenzymatic NADP$^+$/NADPH interconversion. (D) Overlay of the 2nd and 150th CVs in the presence of NADP$^+$, indicating sustained catalytic activity and electrode integrity over extended operation.

The electrochemical stability of FNR immobilized on ITO-modified electrodes (FNR@ITO) was evaluated at pH 9 by cyclic voltammetry under non-turnover conditions. Consecutive CVs were recorded in 100 mM Tris·HCl buffer (pH 9) at a scan rate of 10 mV·s^{-1} in the absence of NADP$^+$

(**Figure S5A**). Over the applied potential window (−0.30 to −0.80 V vs SCE), each CV cycle required approximately 100 s, such that 150 consecutive cycles correspond to ~4.2 h of continuous operation. The voltammograms remained highly reproducible throughout this period, and comparison of the 2nd and 150th scans (**Figure S5B**) shows that the anodic and cathodic current densities were retained to ≥95% of their initial values, indicating minimal loss of electroactive FNR and negligible electrode degradation during prolonged cycling.

The operational stability of FNR@ITO under catalytic conditions was further assessed in the presence of 2 mM $NADP^+$. Introduction of the cofactor resulted in a pronounced increase in current density associated with electroenzymatic $NADP^+$/NADPH interconversion, while preserving the overall voltammetric shape (**Figure S5C**). Importantly, overlay of the 2nd and 150th scans recorded over the same ~4.2 h time frame (**Figure S5D**) revealed sustained catalytic currents with ≥95% current retention, confirming that neither enzyme deactivation nor cofactor-induced fouling occurred under turnover conditions. These results demonstrate the excellent structural and functional stability of FNR@ITO bioelectrodes at pH 9 during extended electrochemical operation.

4 Bidirectional electroenzymatic $NADP^+$/NADPH turnover by FNR@ITO

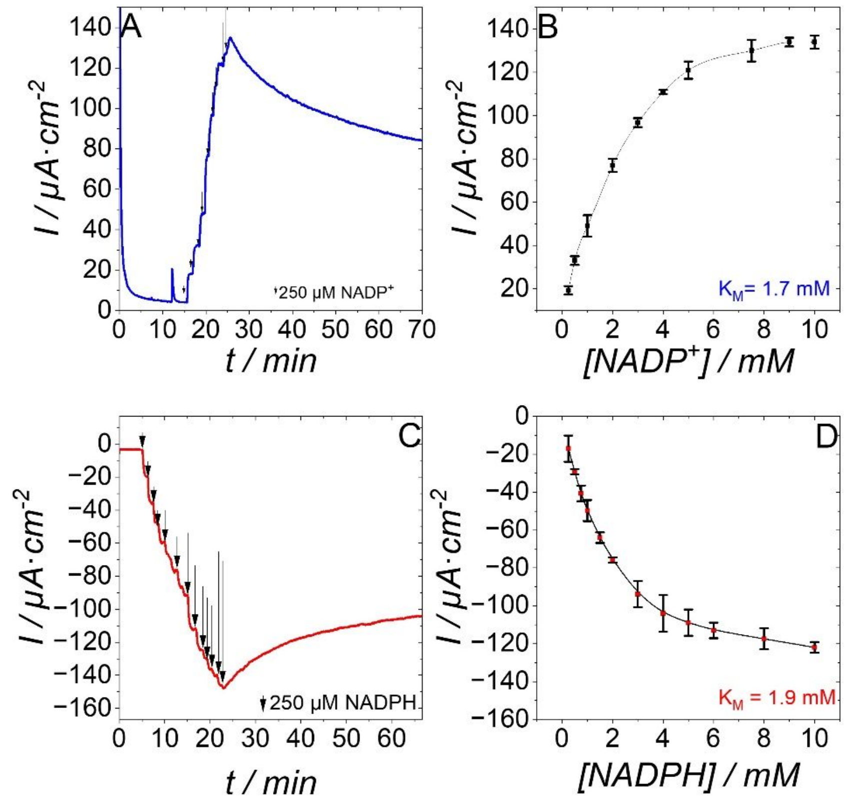

Figure S6. Electroenzymatic oxidation of NADPH and reduction of $NADP^+$ catalyzed by FNR@ITO under bulk electrolysis conditions (100 mM Tris-HCl, pH 9). (A) Chronoamperometric response recorded at −0.6 V vs SHE following successive additions of $NADP^+$, showing the development of a steady-state reductive catalytic current. (B) Dependence of the steady-state reduction current density on $NADP^+$ concentration, fitted to a Michaelis–Menten model to give an apparent K□ = 1.7 mM. (C) Chronoamperometric response recorded at 0 V vs SHE following successive additions of NADPH, showing the development of a steady-state oxidative catalytic current. (D) Dependence of the steady-state oxidation current density on NADPH concentration, fitted to a Michaelis–Menten model to yield an apparent K□ = 1.9 mM.

5 Cyclic voltametric evidence for FNR–ADH LK-mediated alcohol oxidation and ketone reduction

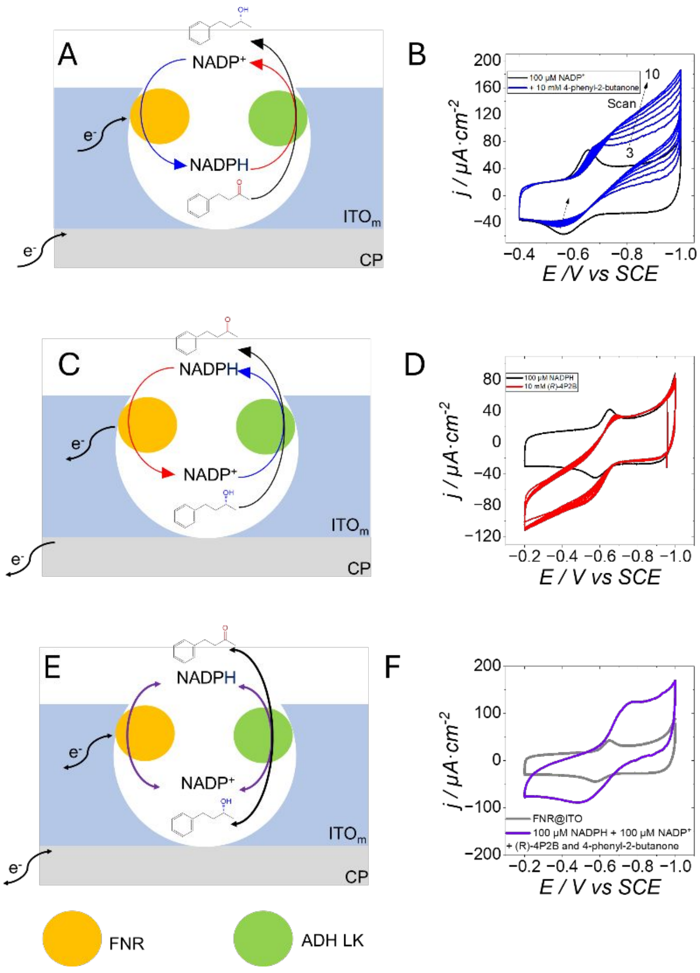

Figure S7. Electroenzymatic interconversion of NADP⁺/NADPH coupled to alcohol oxidation and ketone reduction catalyzed by an FNR–ADH LK assembly immobilized on ITO$_m$. (A, C, E) Schematic representations of the coupled electroenzymatic pathways illustrating FNR-mediated NADP⁺ reduction and NADPH oxidation selectively coupled to ADH LK–catalyzed ketone reduction or alcohol oxidation. (B, D, F) Corresponding cyclic

voltammograms recorded at pH 9 with a scan rate of 2 $mV \cdot s^{-1}$ in the presence of $NADP^+$, NADPH, and **1** or 4-phenyl-2-butanol, showing catalytic current responses associated with the coupled redox processes.

6 Steady-state electroenzymatic kinetics of alcohol–ketone interconversion

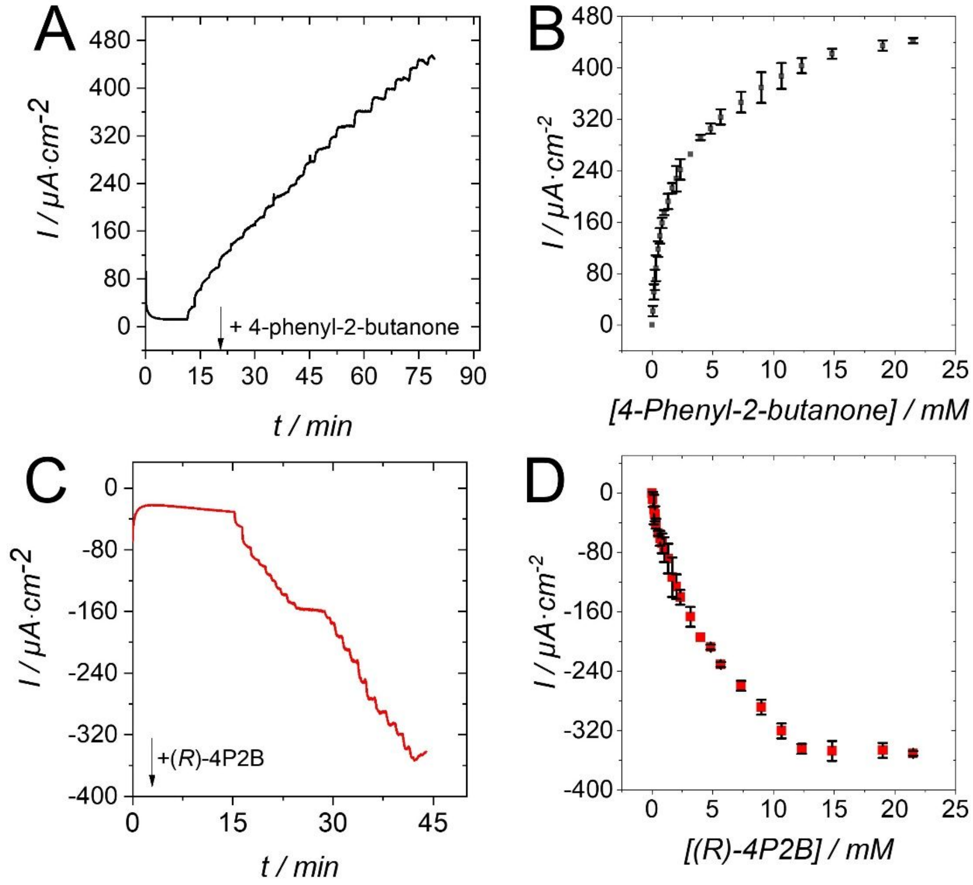

Figure S8. Steady-state kinetic analysis of electroenzymatic ketone reduction and alcohol oxidation catalyzed by FNR–ADH LK@CP–ITOm at pH 9. (A) Chronoamperometric response recorded during bulk electrolysis at −0.6 V vs SHE in 100 mM Tris-HCl buffer (pH 9) with 100 μM $NADP^+$ present from t = 0. Successive additions of **1** induce an increase in reductive current corresponding to ketone reduction coupled to electroenzymatic NADPH regeneration. (B) Dependence of the steady-state reductive current density on **1** concentration. (C) Chronoamperometric response recorded during bulk electrolysis at 0 V vs SHE in the presence of 100 μM NADPH from t = 0. Successive additions of **1′** result in an increase in oxidative current associated with electroenzymatic NADPH oxidation coupled to alcohol oxidation. (D) Dependence of the steady-state oxidative current density on **1′** concentration.

\

7 Electrochemical control of enantioselective alcohol oxidation by FNR–ADH LK@ITO$_m$

Electroenzymatic oxidation of 4-phenyl-2-butanol (**3′**) was evaluated using an FNR–ADH LK@ITO$_m$ electrode carried out at 0 V vs SHE with 5 mM substrate (**Figure S9**). Upon addition of NADPH and **3′**, a pronounced anodic catalytic current developed, reaching an initial maximum before gradually stabilizing over the course of 24 h (**Figure S9A**). This sustained current response is consistent with efficient ADH LK–catalyzed oxidation of the secondary alcohol to the corresponding ketone, coupled to continuous electroenzymatic regeneration of $NADP^+$ by FNR. The gradual decrease in current amplitude over extended electrolysis reflects progressive substrate consumption, while maintaining stable operation under mild, non-forcing electrochemical conditions.

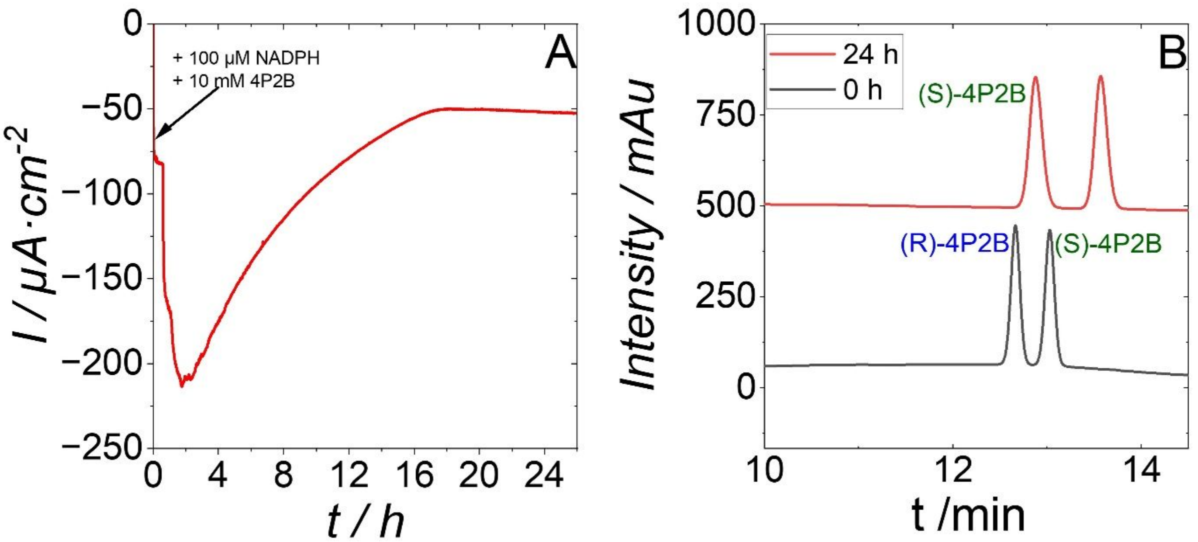

Figure S9. Electroenzymatic oxidation of 3′ catalyzed by an FNR–ADH LK assembly at 0 V vs SHE. (A) Chronoamperometric response recorded upon sequential addition of 100 µM NADPH and 10 mM **3′**, showing the

development of a sustained catalytic current associated with alcohol oxidation coupled to electrochemical NADPH oxidation. (B) Chiral LC chromatograms acquired at 0 h (black) and 24 h (red), demonstrating selective consumption of the **1′**enantiomer, while the **2′** peak remains unchanged, confirming enantioselective oxidation by ADH LK under bulk electrolysis conditions.

Chiral LC analysis confirms that oxidation proceeds with high stereochemical fidelity (**Figure S9B**). Starting from enantiopure **3′**, the chromatogram recorded after 24 h shows selective depletion of the (R)-alcohol peak with no detectable oxidation of the (S)-enantiomer, demonstrating complete enantioselectivity imposed by ADH-LK. This selective oxidation underscores the ability of the FNR–ADH LK assembly to discriminate between enantiomers under electrochemical control, enabling stereospecific alcohol oxidation without racemization or competing non-enzymatic pathways.

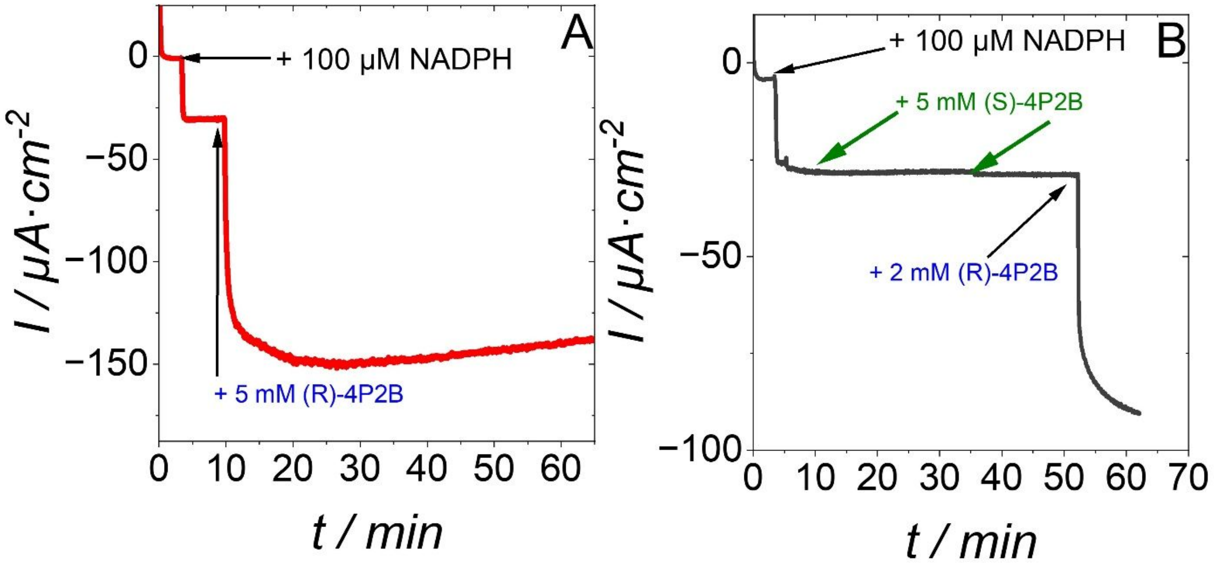

Figure S10. Electroenzymatic oxidation of 1′ catalyzed by an FNR–ADH LK assembly at 0 V vs SHE. (A) Chronoamperometric response following the sequential addition of 100 μM NADPH and 5 mM **1′**, showing the development of a sustained catalytic current associated with selective oxidation of the (R) enantiomer. (B)

Chronoamperometric response recorded under identical conditions upon addition of 100 μM NADPH and 5 mM **2′**, showing no significant increase in catalytic current. Subsequent addition of 2 mM **1′** restores catalytic activity, confirming that the observed current originates exclusively from oxidation of **1′**.

To further substantiate the enantioselectivity observed by chiral LC, bulk electrolysis experiments were performed under identical electrochemical and enzymatic conditions using enantiopure alcohol substrates (**Figure S10**). Upon addition of 100 μM NADPH, only a small background current was observed. Subsequent addition of 5 mM **1′** induced a pronounced increase in catalytic current, reaching approximately −150 $\mu A \cdot cm^{-2}$ and stabilizing over time (**Figure S10A**). This response is consistent with efficient ADH LK–catalyzed oxidation of the (R)-alcohol coupled to FNR-mediated electrochemical oxidation of NADPH.

In contrast, addition of 5 mM **2′** under otherwise identical conditions resulted in no significant change in current beyond the NADPH background (**Figure S10B**), indicating that the (S)-enantiomer is not appreciably oxidized by ADH LK. Subsequent introduction of **1′** into the same electrolyte immediately restored catalytic activity, yielding a current response comparable to that observed in **panel A**. These bulk electrolysis experiments directly demonstrate that the observed catalytic current originates exclusively from oxidation of the (*R*)-enantiomer, confirming that enantioselectivity is preserved under electrochemical control and arises from intrinsic substrate discrimination by ADH LK rather than from downstream separation or analytical bias.

8 Influence of AC electrolysis geometry on paired electroenzymatic HIE efficiency

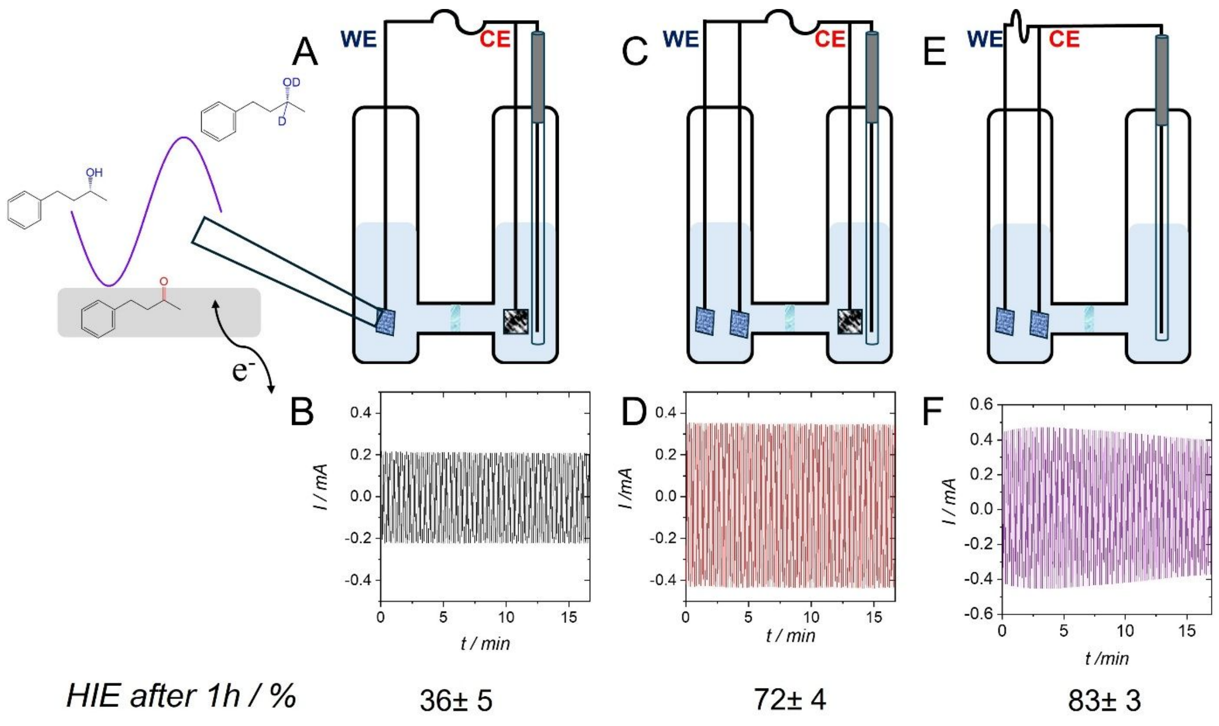

Figure S11. Effect of AC electrolysis geometry on electroenzymatic HIE efficiency. (A, B) Single–working-electrode configuration subjected to an alternating potential, where oxidative and reductive half-cycles occur sequentially at the same interface. (C, D) Dual working electrodes operated in parallel under the same AC waveform, enabling independent but simultaneous redox cycling at each electrode. (E, F) Working–counter paired configuration in which the electrodes alternate between oxidative and reductive roles each half-cycle, establishing spatially complementary redox processes. The corresponding chronoamperometric traces (B, D, F) show progressively increasing current amplitudes that correlate with enhanced HIE yields after 1 h (36 ± 5%, 72 ± 4%, and 83 ± 3%, respectively). Experiments were conducted in 200 µL electrolyte at pD 9 (100 mM Tris–DCl) containing 10 mM **1′**, 50 µM $NADP^+$, and 50 µM NADPH, using an AC frequency of 0.1 Hz, a potential amplitude of 0.3 V, and an init potential of −0.3 V vs SHE (reference electrode: SCE).

To elucidate how electrode configuration influences redox symmetry and catalytic coupling, three AC electrolysis geometries were evaluated for 1 h under identical conditions (**Figure S11**). In the

first configuration (**A, B**), a single working electrode was subjected to an alternating potential sweeping between oxidation and reduction. The modest current amplitude (±0.2 mA) and limited HIE yield (36 ± 5%) indicate that both $NADP^+$ reduction and NADPD oxidation occur sequentially on the same surface. While this demonstrates self-contained bidirectional FNR activity, the reaction zone remains confined to one interface. When two working electrodes were operated in parallel under the same waveform (**C, D**), each performed identical redox cycling independently. The current amplitude increased (±0.37 mA) and the HIE yield reached 72 ± 4 %, reflecting the additive effect of two isolated yet simultaneous enzymatic sites. However, because both electrodes experience the same polarity at any given instant, inter-electrode exchange of intermediates is minimal.

The highest performance was achieved in the working–counter configuration (**E, F**), where the electrodes alternated roles each half-cycle. Here, reduction at one surface coincides with oxidation at the other, allowing products and cofactors to diffuse between them. This spatial complementarity generated the strongest current oscillations (±0.6 mA) and the highest HIE yield (83 ± 3 %), characteristic of a true paired enzymatic electrolysis in which dynamic cross-communication between electrodes sustains continuous cofactor regeneration and efficient substrate conversion. In this configuration, AC electrolysis effectively integrates two mechanistic regimes: it retains the local bidirectional character of the single-electrode system, where intermediates are alternately generated and consumed at the same interface, while simultaneously incorporating the cooperative behavior of a DC-type cell, where the complementary half-reactions occur on opposing electrodes. The periodic reversal of polarity therefore unifies both phenomena—local redox cycling and inter-electrode mass transfer—into a single oscillatory

process, maximizing electron utilization and coupling efficiency between FNR and ADH LK mediated transformations.

9 Influence of electrolysis regime on cofactor cycling efficiency in HIE

Given the markedly higher electrochemical engagement observed under AC electrolysis compared to DC operation (**Figure S12**), examining the influence of nicotinamide cofactor concentration became essential, particularly in the presence of 5 mM **1′**.

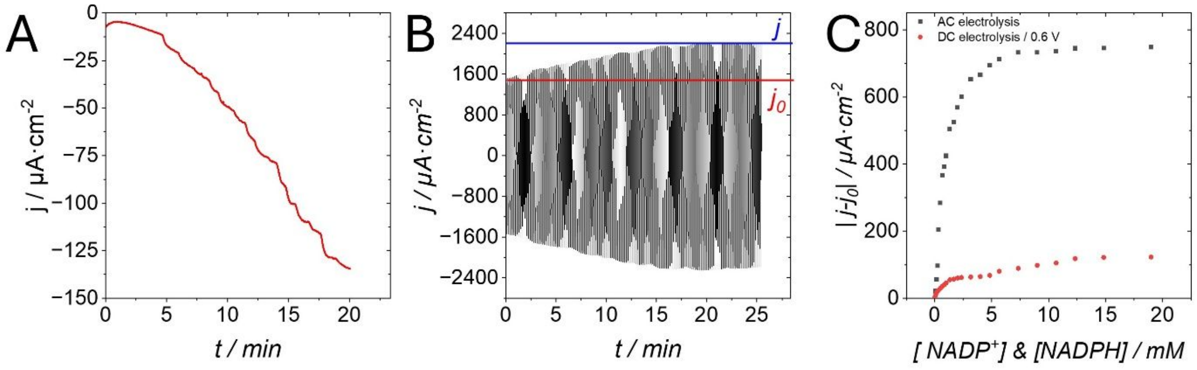

Figure S12. Comparison of DC and AC electrolysis as a function of nicotinamide cofactor concentration under identical catalytic conditions. (A) Chronoamperometric response under DC electrolysis (0.6 V vs CE) recorded in a 3 mL electrochemical cell containing 5 mM **1′**, with identical FNR/ADH LK loadings on both electrodes, upon successive equimolar additions of $NADP^+$ and NADPH. (B) Chronoamperometric trace under AC electrolysis measured under the same conditions and successive equimolar additions of $NADP^+$ and NADPH. (C) Dependence of the absolute electrolysis current ($|j-j_0|$) on total nicotinamide cofactor concentration, obtained by successive equimolar additions of $NADP^+$ and NADPH, highlighting the sharp current response and early saturation characteristic of AC electrolysis compared to DC operation.

Analysis of the electrolysis response as a function of total $NADP^+$/NADPH concentration focuses on the evolution of the current with successive cofactor additions, rather than its absolute

magnitude. Under AC electrolysis, the current increases sharply upon addition of small amounts of both cofactors and rapidly approaches saturation (**Figures S12B and S12C**), reflecting efficient engagement of the nicotinamide pool. In contrast, DC electrolysis exhibits only a gradual, attenuated current response that plateaus at substantially lower values (**Figures S12A and S12C**), indicative of limited cofactor utilization. These trends demonstrate that AC operation sustains high catalytic activity at markedly reduced nicotinamide concentrations relative to DC electrolysis.

10 Efficient electroenzymatic HIE under AC operation at low cofactor concentrations

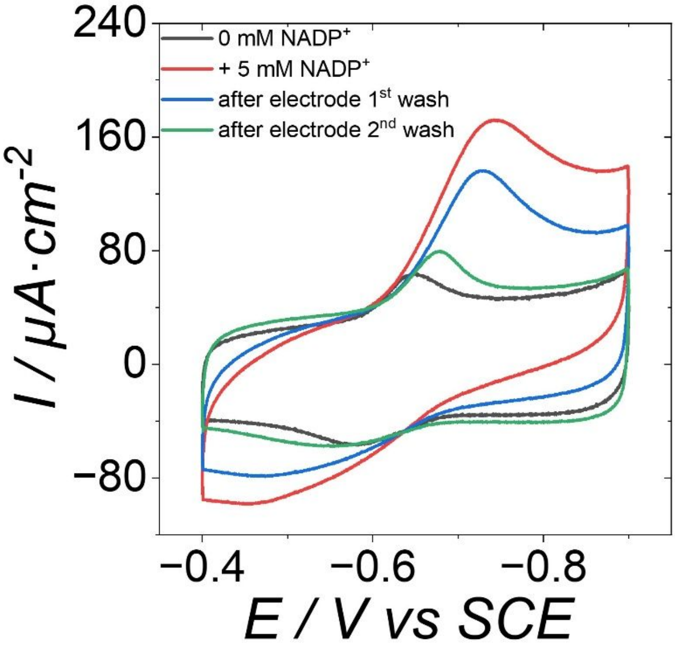

Figure S13. Evidence for NADP$^+$ confinement within porous FNR@ITO$_m$ electrodes. CV recorded for FNR FNR@ITOm in the absence of added cofactor (black trace), after addition of 5 mM NADP$^+$ (red trace), and following successive electrode washing steps (blue and green traces) in the absence of soluble cofactor in the electrolyte. Measurements were performed at 5 mV·s^{-1} in 100 mM Tris–HCl buffer (pH 9).

Before evaluating system performance under AC electrolysis, we first examined the electrochemical response of FNR@ITO_m toward $NADP^+$. CVs display the characteristic reversible redox features of FNR, and addition of 5 mM $NADP^+$ induces a pronounced increase in catalytic current. Notably, a substantial fraction of this response persists after successive electrode washing steps, even in the absence of soluble cofactor in the electrolyte (**Figure S13**), indicating confinement of $NADP^+$ within the porous ITO architecture. Such confinement implies a locally elevated effective $NADP^+$ concentration within the nanopores, enabling cofactor cycling to proceed within the electrode environment, and consequently allowing the use of substantially lower bulk cofactor concentrations while maintaining HIE performance comparable to that obtained at higher nicotinamide loadings.

Table S1. Effect of initial $NADP^+$/NADPH concentration on deuteration efficiency under AC electrolysis. TTN_{NADP} values were calculated as: TTN = (2 × [**1′**] + [**1**]) / ($[NADP^+]_0$+ $[NADPH]_0$).All experiments performed with 10 mM **1′** in Tris–DCl buffer (pD 9), 0.6 V amplitude, 0.1 Hz frequency, and identical 1 nmol FNR/0.3 nmol ADH LK–modified ITO electrodes separated by 1 mm.

Entry	$[NADP^+]$ /µM	[NADPH] / µM	t /h	TTN_{NADP}
**(a)**	5	5	2.5	1750 ± 21
**(b)**	10	-	2.5	1723± 15

The strong increase in AC electrolysis current upon the addition of even small concentrations of both cofactors (**Figure S13**) has brought the motivation towards the study HIE within 1 order of magnitude less of cofactors. As shown in **Table S1**, decreasing the total cofactor concentration by

one order of magnitude does not compromise isotopic exchange: nearly 100% deuteration is still obtained, accompanied by a TTN_{NADP} of 1750 after 2.5 h (**entry a**). Because $NADP^+$ is considerably less expensive than NADPH, the ability of the system to function efficiently when only the oxidized form is supplied is particularly advantageous. In this case, prior to the onset of AC electrolysis, $NADP^+$ undergoes a limited enzymatic reduction by the alcohol substrate, producing nanomolar amounts of NADPD. This small initial fraction of reduced cofactor is sufficient to initiate $NADP^+$/NADPD cycling once the alternating potential is applied. After 2.5 h of AC electrolysis, the system (**entry b**) exhibits essentially the same outcome as when both cofactors (**entry a**) are supplied—near-quantitative HIE and a high TTN—demonstrating that AC operation sustains efficient cofactor activation, interconversion, and turnover even when only $NADP^+$ is present initially.

11 Generality of AC-driven electroenzymatic HIE

With the efficiency of ADH LK mediated HIE established under AC driven cofactor regeneration, we next examined the generality of the platform across a broader range of alcohol substrates. Because ADH LK activity is highly sensitive to substrate structure,[6,7] extending the study beyond the model compound enables delineation of the enzyme's operational window under electrochemical conditions. Moreover, evaluation of additional aromatic secondary alcohols is critical for assessing the electrosynthetic relevance of this approach, particularly for the preparation of deuterated pharmaceutical intermediates. Accordingly, a substrate-scope investigation was conducted to probe the influence of substrate identity on electroenzymatic HIE.

As a case study, (*R*)-1-naphthylethanol was selected because ADH LK exhibits lower catalytic activity toward this substrate relative to the reference compound, **1′**. Enzymatic activity was quantified by mixing $NADP^+$, ADH LK, and the alcohol and monitoring the increase in absorbance at 340 nm, which reports NADPH formation during alcohol oxidation. In control experiments, $NADP^+$ and alcohol alone did not produce any change in absorbance (**curve x Figure S14A**). Over the first 10 min, (*R*)-1-naphthylethanol generated a significantly smaller A340 increase (**curve a Figure S14A** ; slope = 0.005 a.u. min^{-1}) than the reference compound (**curve y Figure S14A** ; slope = 0.018 a.u. min^{-1}). Increasing the amount of ADH LK improved the activity toward (*R*)-1-naphthylethanol, yielding a slope of 0.015 a.u. min^{-1} (**curve b Figure S14A**). These results provide a quantitative basis for designing electroenzymatic conditions suitable for slower substrates.

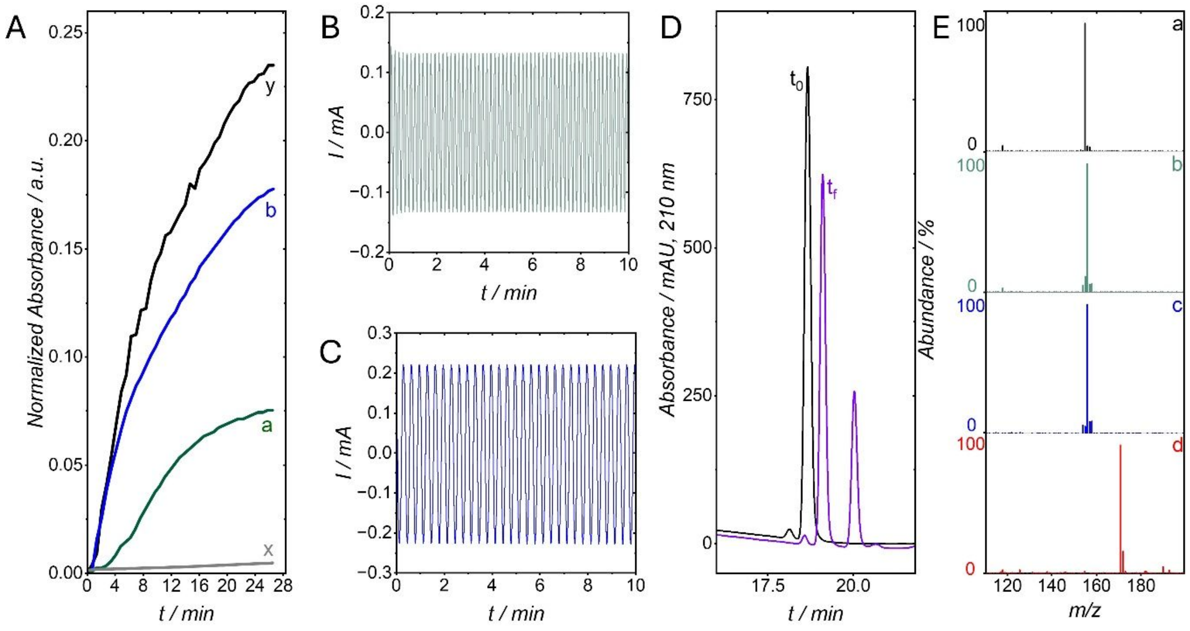

Figure S14. Electrochemical and analytical characterization of AC-driven deuteration of (*R*)-1-naphthylethanol at different frequencies and enzyme loadings. (A) UV–vis enzymatic activity assay (96-well plate) performed in the presence of 0.5 mM $NADP^+$, ADH LK, and the corresponding substrate. Curve x corresponds to a control experiment containing **1′** and 0.5 mM $NADP^+$ in the absence of ADH LK. Curve y corresponds to the reference reaction with **1′**, 0.5 mM $NADP^+$, and 0.3 nmol ADH LK. Trace a shows the activity toward (*R*)-1-naphthylethanol using the same ADH LK amount (0.3 nmol) and 0.5 mM $NADP^+$, while trace b shows the response obtained when the ADH LK amount is doubled. (B) Chronoamperometric response under AC electrolysis at 0.05 Hz using single ADH LK loading (0.3 nmol), displaying the characteristic oscillatory current pattern. (C) Chronoamperometric response under AC electrolysis at 0.1 Hz using two-fold ADH LK loading (0.6 nmol). (D) Analytical LC chromatograms recorded before electrolysis (t_0, black trace) and after AC electrolysis (t, purple trace). (E) Mass spectra of key species. Panel a: starting (*R*)-1-naphthylethanol. Panel b: (*R*)-1-naphthylethanol at t_f obtained under 0.05 Hz AC electrolysis with single ADH LK loading. Panel c: (*R*)-1-naphthylethanol at t_f under 0.1 Hz AC electrolysis with doubled ADH LK loading. Panel d: mass spectrum of the peak obtained at 20 min in LC, corresponding to the ketone.

To accommodate the intrinsically slower turnover of (*R*)-1-naphthylethanol within the AC electroenzymatic framework, two orthogonal kinetic levers were evaluated: increasing the surface loading of ADH LK to enhance catalytic site density or decreasing the AC frequency to prolong each redox half-cycle and thereby extend the enzymatic reaction window prior to polarity inversion. While reduced frequencies increase catalytic residence time, they necessarily impose longer electrolysis durations to achieve efficient HIE.

Both strategies were examined under otherwise identical conditions. At 0.05 Hz with a onefold ADH LK loading, symmetric current oscillations of ±0.15 mA were observed (**Figure S14B**), indicating balanced and sustained NADPD/NADP$^+$ cycling over the extended 10 s half-cycle. In contrast, operation at 0.1 Hz with the two folds ADH LK loading generated larger oscillations (±0.23 mA; **Figure S14C**), consistent with a higher instantaneous electron flux and more frequent redox turnover. Despite these distinct kinetic regimes, chiral LC analysis revealed comparable product distributions after electrolysis (68 ± 7% alcohol and 30 ± 5% ketone; **Figure S14D**). Mass spectrometry confirmed near-quantitative isotopic incorporation in both cases (89 ± 6% at 0.05 Hz **panel b** and 92 ± 5% at 0.1 Hz **panel c**; **Figure S14E**), with deuterium content quantified from the increase of the +1 Da relative to the undeuterated m/z 155 signal. The marginally lower incorporation observed at 0.05 Hz is attributed to partial loss of enzymatic activity over the extended electrolysis period.

Collectively, these results demonstrate that AC electrolysis provides a tunable kinetic framework capable of compensating for reduced enzymatic turnover either through modulation of enzyme loading or temporal control of redox cycling. For consistency across the substrate scope—and given the small reaction volumes and enzyme castings used—adjusting the ADH LK amount was

generally the preferred strategy, although excessive ADH LK deposition can partially inhibit FNR activity. These findings establish enzyme density–waveform matching as a critical design principle for maximizing efficiency in AC-driven electroenzymatic HIE.

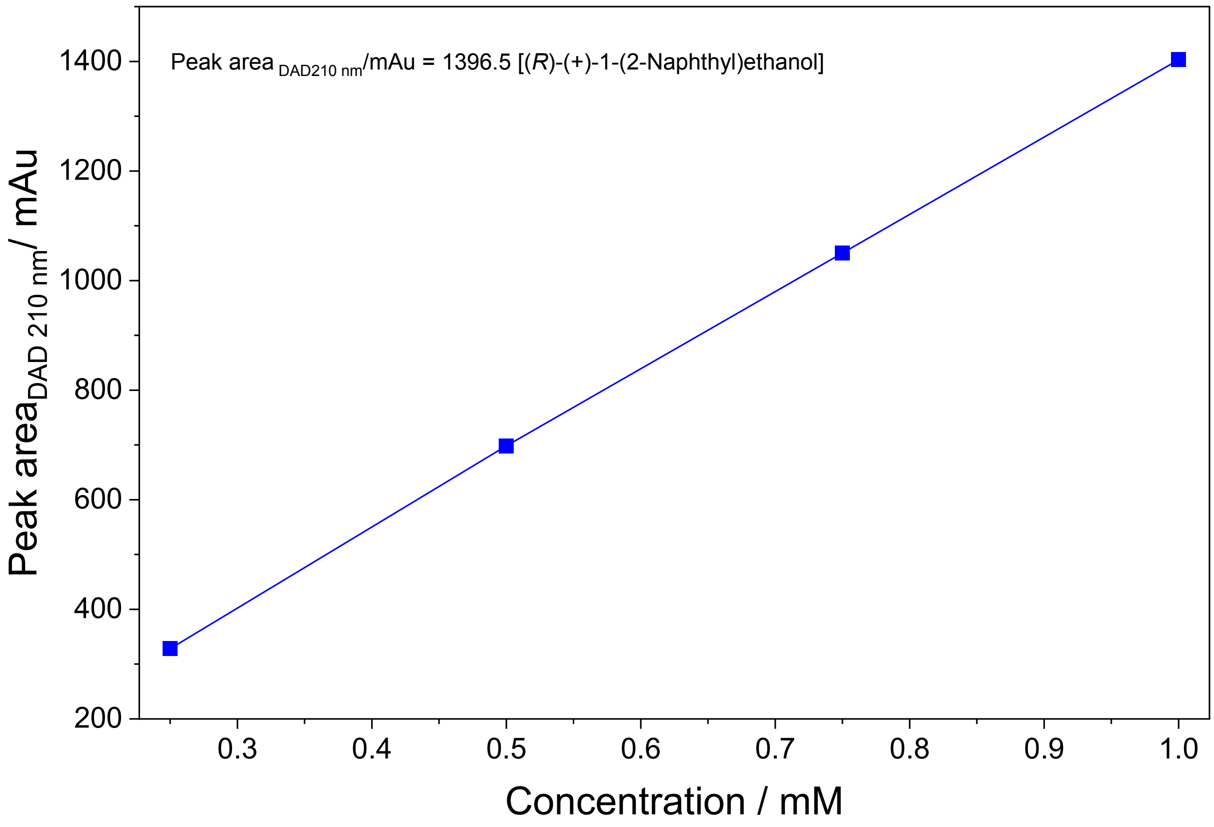

Figure S15. LC calibration curve for quantitative determination of the (R)-(+)-1-(2-naphthyl)ethanol standard showing linear dependence of peak area on analyte concentration.

12 Fuel-Driven, waveform-controlled electroenzymatic HIE

At pH 9, FNR mediates the $NADP^+$/NADPH (NADPD) couple with a formal potential $E^{\circ\prime} \approx -0.38$ V vs SHE. This potential lies thermodynamically between H_2 oxidation ($H_2/2H^+$, $E^{\circ\prime} \approx -0.53$ V vs SHE at pH 9) and O_2 reduction (O_2/H_2O, $E^{\circ\prime} \approx +0.70$ V vs SHE at pH 9). Exploiting this alignment, HIE can be operated in a fuel-cell-like mode without external polarization (**Figure S16A**). Under H_2, H_2 oxidation at the Pt electrode (1 cm^2 gas diffusion electrode 0.5 mg Pt/cm^2) supplies electrons at a sufficiently reducing potential to drive electroenzymatic reduction of $NADP^+$ to NADPD via FNR, which in turn fuels ADH-catalyzed ketone reduction to the deuterated alcohol. Switching the atmosphere to O_2 reverses the redox bias: O_2 reduction withdraws electrons, enabling oxidation of NADPD back to $NADP^+$, coupled to alcohol oxidation to the ketone. Periodic H_2/O_2 switching, controlled by an Arduino-defined waveform, thus enforces alternating reductive and oxidative half-cycles, allowing HIE to proceed autonomously through fuel-cell-driven cofactor cycling, with reaction direction dictated solely by relative redox potentials and periodic H_2/O_2 switching.

The open-circuit potential (E_{OCP}) was monitored during HIE experiments performed in 100 mM Tris–DCl buffer containing 100 μM $NADP^+$ and 5 mM **1**′. E_{OCP} is reported vs the Pt counter electrode, which simultaneously serves as the gas-exposed electrode. Under these conditions, the E_{OCP} response reflects the instantaneous redox bias imposed by the gas environment (**Figure S16B**). Positive E_{OCP} values correspond to reductive conditions, arising from H_2 oxidation at the Pt electrode, which supplies electrons to the enzymatic cascade and drives electroenzymatic reduction of the ketone to the alcohol through FNR-mediated $NADP^+ \rightarrow$ NADPD cycling. In contrast, negative E_{OCP} values indicate oxidative conditions, in which reversal of the redox bias

promotes oxidation of the alcohol back to the ketone, coupled to electroenzymatic regeneration of $NADP^+$ from NADPD. The alternating sign and magnitude of E_{OCP} thus directly report the oscillated switching between reductive and oxidative HIE half-cycles under fuel-driven operation.

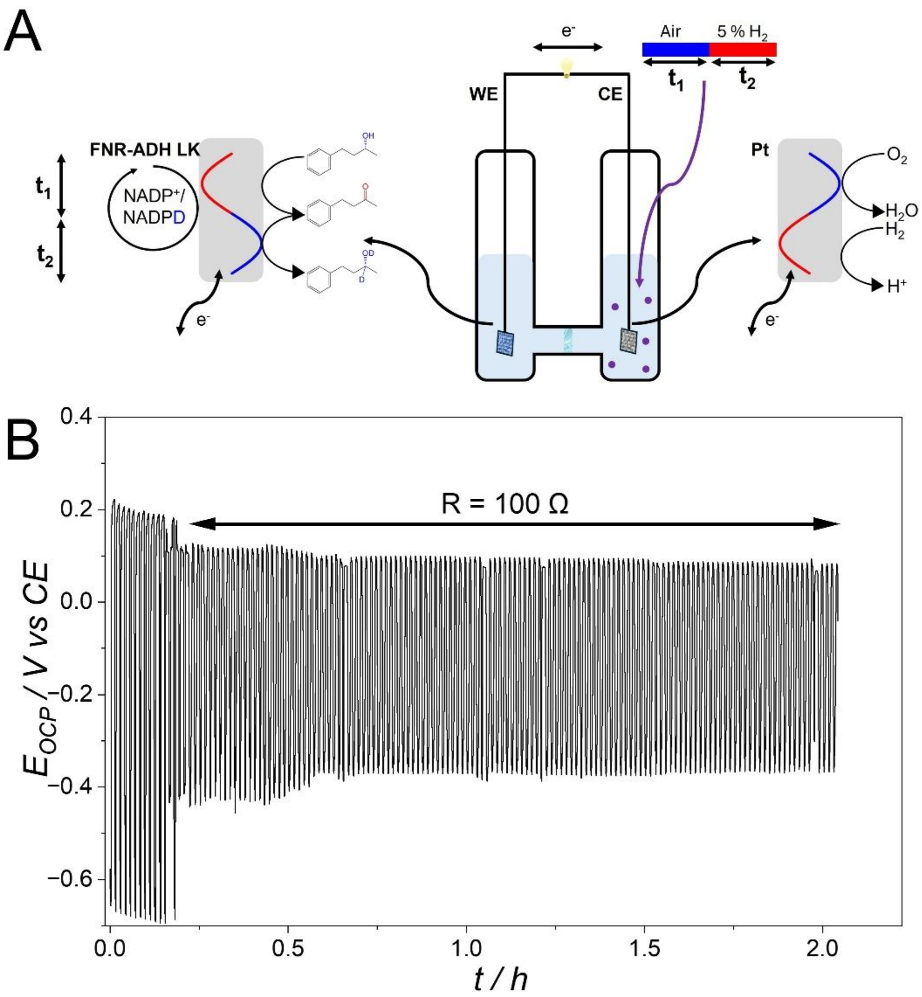

Figure S16. Fuel-driven electroenzymatic HIE. (A) Schematic representation of a fuel-cell-like electrochemical configuration in which alternating H_2 and O_2 supply reversibly switches electron flow to drive FNR-mediated cycling of the $NADP^+$/NADPD couple and thereby enables sequential ketone reduction and alcohol oxidation during HIE. (B) E_{OCP} trace was recorded during HIE experiments performed with 100 μM $NADP^+$ and 5 mM **1′** in 100 mM Tris–DCl buffer, using 1 mL of Tris-DCl solution in each compartment of an H-cell. The working electrode (1 cm^2) was modified with ITO and subsequently coated with immobilized FNR and ADH LK, while the counter electrode consisted of a 1

cm² gas-diffusion electrode loaded with Pt (0.5 mg·cm^{-2}). Potentials are reported vs the Pt counter electrode. Positive E_{OCP} values correspond to reductive conditions associated with H_2 oxidation at the Pt electrode and ketone reduction, whereas negative E_{OCP} values indicate oxidative conditions coupled to alcohol oxidation and NADPD → $NADP^+$ regeneration.

At the beginning of the experiment, the cell was operated under open-circuit conditions while the counter-electrode atmosphere was alternated between air and 5% H_2 using 10 s half-cycles. During this initial period (t < 0.2 h), E_{OCP} oscillated between approximately +0.15 to +0.20 V vs CE under H_2 and −0.45 to −0.60 V vs CE under air. These potential plateaus were reproducibly reached within each half-cycle, indicating that the chosen switching period was sufficient to establish a well-defined redox environment in the counter-electrode compartment despite the use of diluted fuel (5% H_2). After 0.2 h, an external 100 Ω resistive load was introduced, resulting in a clear attenuation of the oscillation amplitude. Under load, the potential stabilized to oscillate between ca. +0.05 to +0.10 V vs CE during H_2 exposure and −0.25 to −0.35 V vs CE during air exposure. This decrease in absolute potential magnitude is consistent with the onset of current flow through the external circuit, leading to iR losses and electrode overpotentials. Importantly, the polarity and temporal regularity of the oscillations were preserved over the subsequent ~2 h of operation, confirming sustained electron flow and reversible switching between reductive (ketone reduction via $NADP^+$ → NADPD) and oxidative (alcohol oxidation coupled to NADPD → $NADP^+$) HIE half-cycles under fuel-driven conditions.

Under these operating conditions, sustained oscillatory redox cycling translated directly into chemical turnover. After 2 h of operation, the reaction reached 61% of HIE, as determined by LC–MS analysis of the alcohol product. This level of isotopic incorporation confirms that the alternating reductive and oxidative bias imposed by gas switching is not merely electrochemically

stable but chemically productive, enabling continuous ketone reduction and alcohol oxidation cycles coupled to energy production. Based on the measured potential oscillations under a 100 Ω external load, the energy dissipated across the resistor during 2 h of operation is estimated to be on the order of 1 J, indicating that a small but continuous electrical current accompanies productive fuel driven HIE.

Despite the significance of these results in demonstrating fuel-driven, waveform-controlled electroenzymatic HIE, the present configuration also highlights practical considerations for future implementation. Gas switching was regulated using an external Arduino controller, underscoring the feasibility of programmable operation; however, the requirement for an external power source to operate the control electronics indicates that, in its current form, the system is not energetically autonomous. While electrical energy is generated during operation, additional energy input is still required to sustain control and actuation. Achieving net autonomous operation would therefore necessitate larger-scale reactors capable of delivering higher electrical output to support both chemical transformation and system control. Moreover, the need to sufficiently saturate each compartment with H_2 and O_2 imposes relatively long half-cycles and low operating frequencies, pointing toward diffusion-based gas delivery strategies as a promising avenue for future development.

13 Appendix

13.1 Electroenzymatic ketone reduction and deuterium incorporation (Figure 3 in the manuscript)

13.1.1 LC–MS analysis

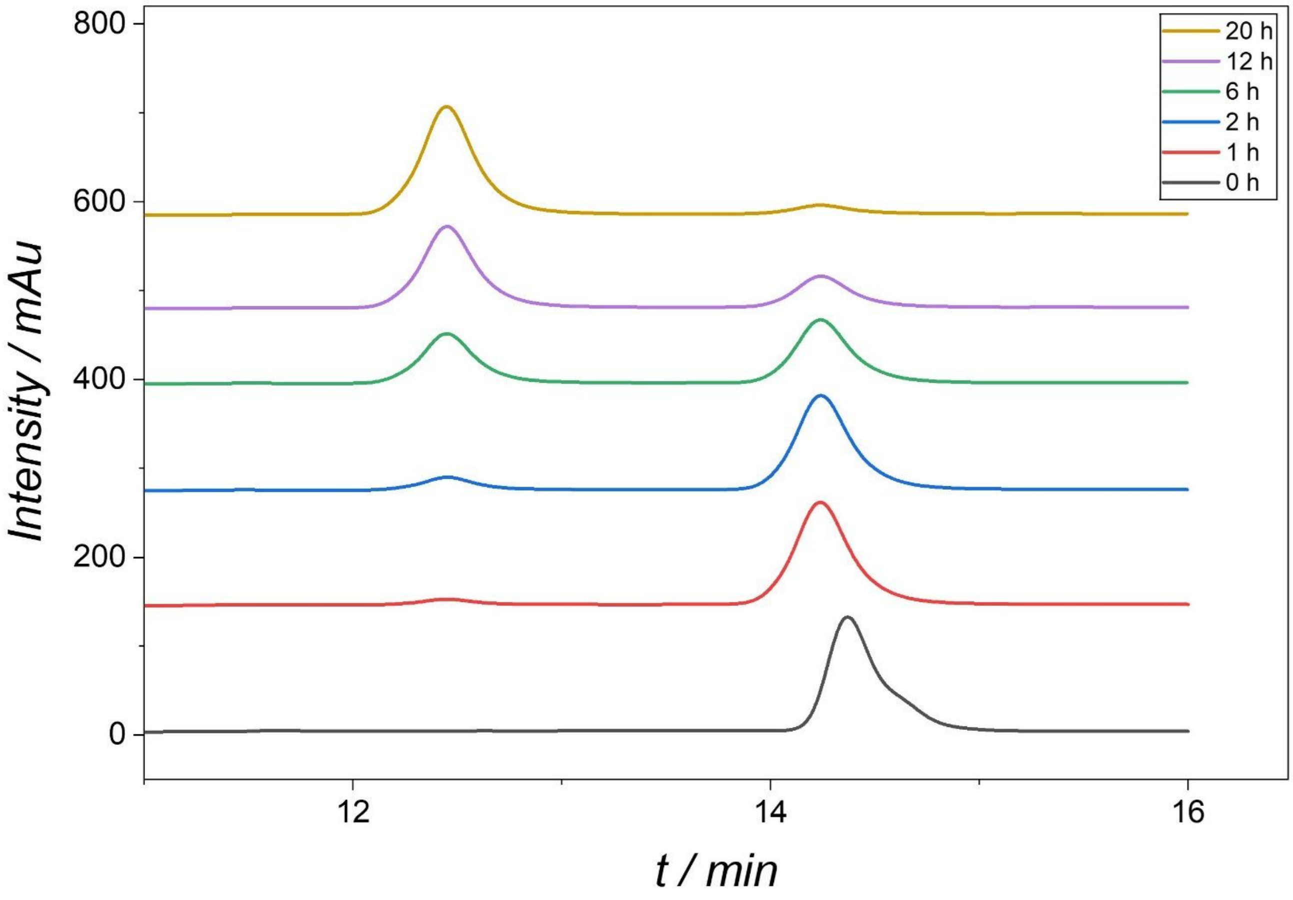

Figure A1. Time-resolved LC chromatograms (DAD, 210 nm) illustrating the electroenzymatic reduction of NADP⁺ catalyzed by FNR coupled with the reduction of 1 to 1′ catalyzed by ADH LK. Traces recorded at 0, 1, 2, 6, 12, and 20 h show the progressive consumption of the ketone and concomitant formation of the alcohol product under reductive electrolysis conditions. The increase in the **1′** peak intensity with time reflects sustained NADPD regeneration from NADP⁺ and its utilization by ADH LK to drive stereoselective ketone reduction.

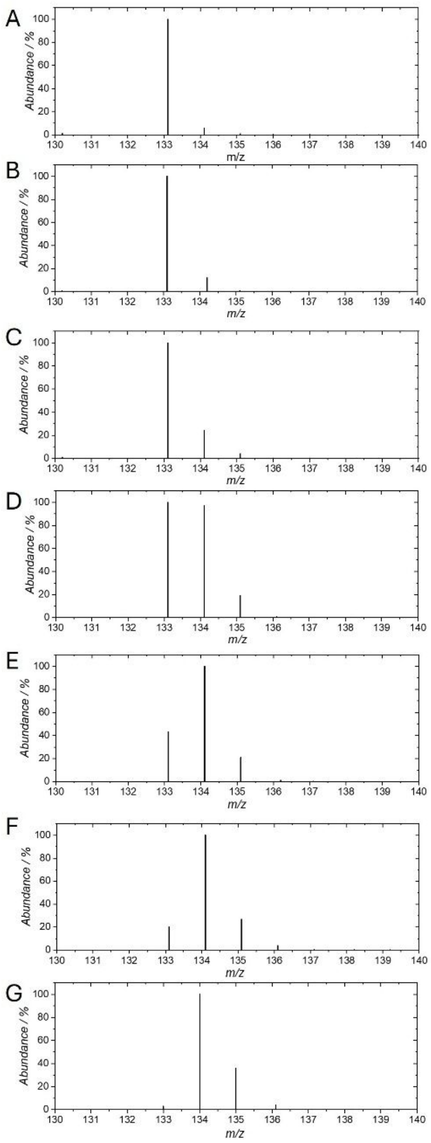

Figure A2. Mass spectra of the (*R*)-alcohol peak eluting at 12.6 min obtained during the electroenzymatic ADH LK–catalyzed reduction of 1 to [²H] 1′. Panels correspond to increasing D_2O fractions in the electrolyte: A, 0% D_2O; B, 2% D_2O; C, 10% D_2O; D, 40% D_2O; E, 60% D_2O; F, 80% D_2O; G, 100% D_2O. The progressive shift of the molecular ion envelope to higher m/z (M+1) values reflects increasing deuterium incorporation arising from FNR-mediated regeneration of deuterated nicotinamide cofactors coupled to ADH LK–catalyzed ketone reduction.

13.1.2 ^{1}H NMR analysis

13.1.2.1 13.1 Electroenzymatic ketone reduction(1)

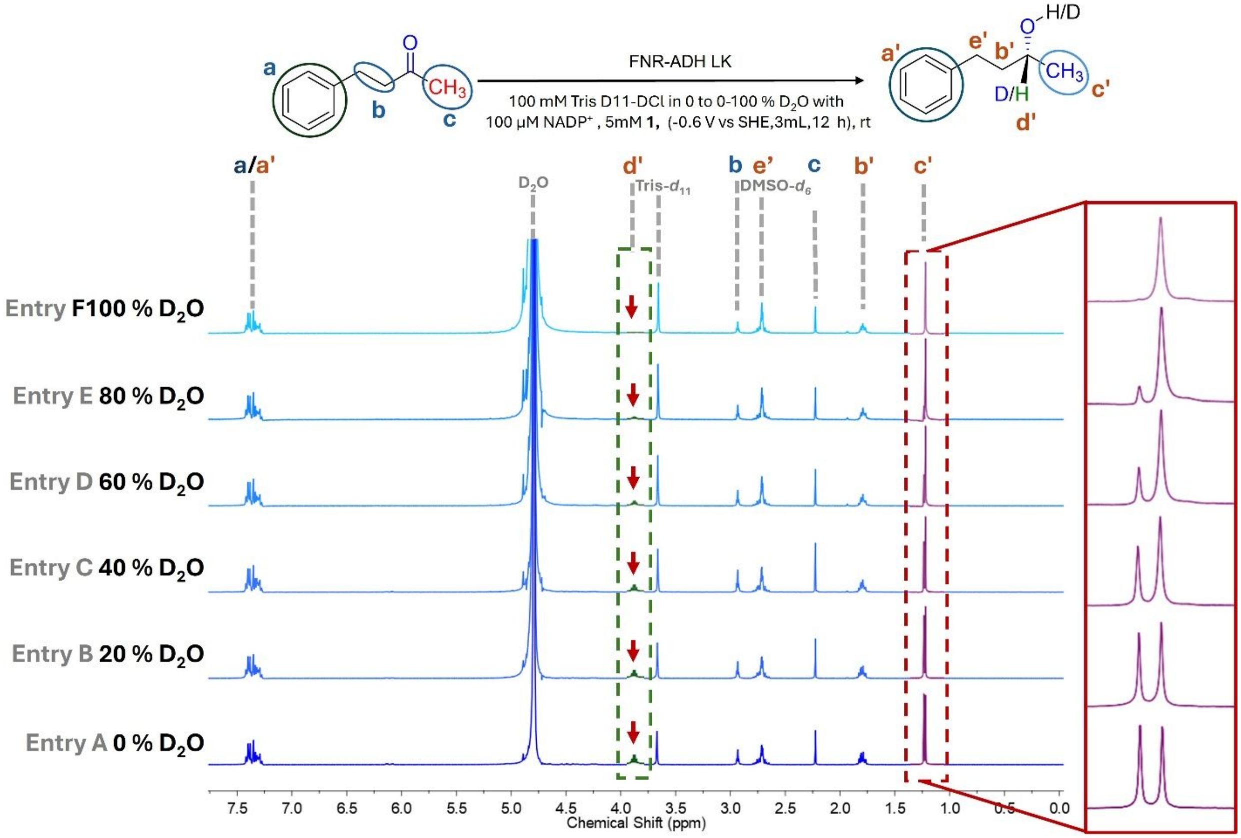

Figure A 3. ^{1}H NMR analysis of D_2O-dependent electroenzymatic ketone reduction to deuterated alcohol. ^{1}H NMR spectra recorded after electroenzymatic reduction of **1** using FNR–ADH LK in media containing 0–100% D_2O. Peak assignments are shown for the ketone starting material and alcohol product: **a/a′** = aromatic protons; **b** = benzylic CH_2 protons of the ketone; **c** = methyl protons of the ketone; **e′** = benzylic CH_2 protons of the alcohol product; **b′** = CH_2 protons adjacent to the alcohol stereocenter; **c′** = methyl protons of the alcohol product; and **d′** = methine proton at the alcohol stereocenter. Increasing D_2O content leads to progressive loss of the **d′** signal, indicating replacement of the stereocenter C–H by C–D. The **c′** methyl resonance also collapses from a doublet to a singlet as coupling to the adjacent methine proton is removed upon deuterium incorporation. At 100% D_2O, disappearance of **d′** together with the singlet **c′** resonance indicates complete formation of the α-deuterated alcohol. Conditions: FNR–ADH LK, 100

mM Tris-d_{11}–DCl buffer (pD 9), 0–100% D_2O, 100 μM $NADP^+$, 5 mM **1**,–0.6 V vs SHE, 3 mL, 12 h, room temperature.

Electroenzymatic reduction of the ketone substrate was performed in reaction media containing varying fractions of D_2O (0–100%) under otherwise identical conditions. After electrolysis, reaction mixtures were analyzed by ^1H NMR spectroscopy. The resonance corresponding to the methine proton at the alcohol stereogenic center (d′) decreased progressively with increasing D_2O content and was not detected in the spectrum recorded in 100% D_2O. In parallel, the methyl resonance of the product (c′) evolved from a doublet to a singlet, consistent with loss of scalar coupling to the adjacent methine proton upon replacement by deuterium. These spectral changes confirm that deuterium incorporation occurs during electroenzymatic ketone reduction and that the extent of incorporation increases with the fraction of D_2O in the reaction medium.

13.1.2.2 Evaluation of background hydrogen–deuterium exchange of the ketone.

To assess whether undesired hydrogen–deuterium exchange occurs at positions other than the stereogenic center during the reaction, the stability of the ketone intermediate **1**was examined under the reaction medium. The ketone was incubated in 100 mM Tris-d_{11}–DCl buffer (pD 9) containing 5% DMSO and nicotinamide cofactors (100 μM $NADP^+$ and NADPH), and aliquots were analyzed by ^1H NMR spectroscopy over a period of 10 h (**Figure A4**). The signals corresponding to the β-methylene and terminal methyl groups remained unchanged in both chemical shift and multiplicity throughout the experiment, indicating no detectable hydrogen–deuterium exchange at the β-position of the ketone under these conditions. These results confirm that background isotopic exchange of the ketone intermediate in the reaction medium is negligible and that deuterium incorporation observed in the electroenzymatic reactions arises from enzymatic reduction mediated by NADPD.

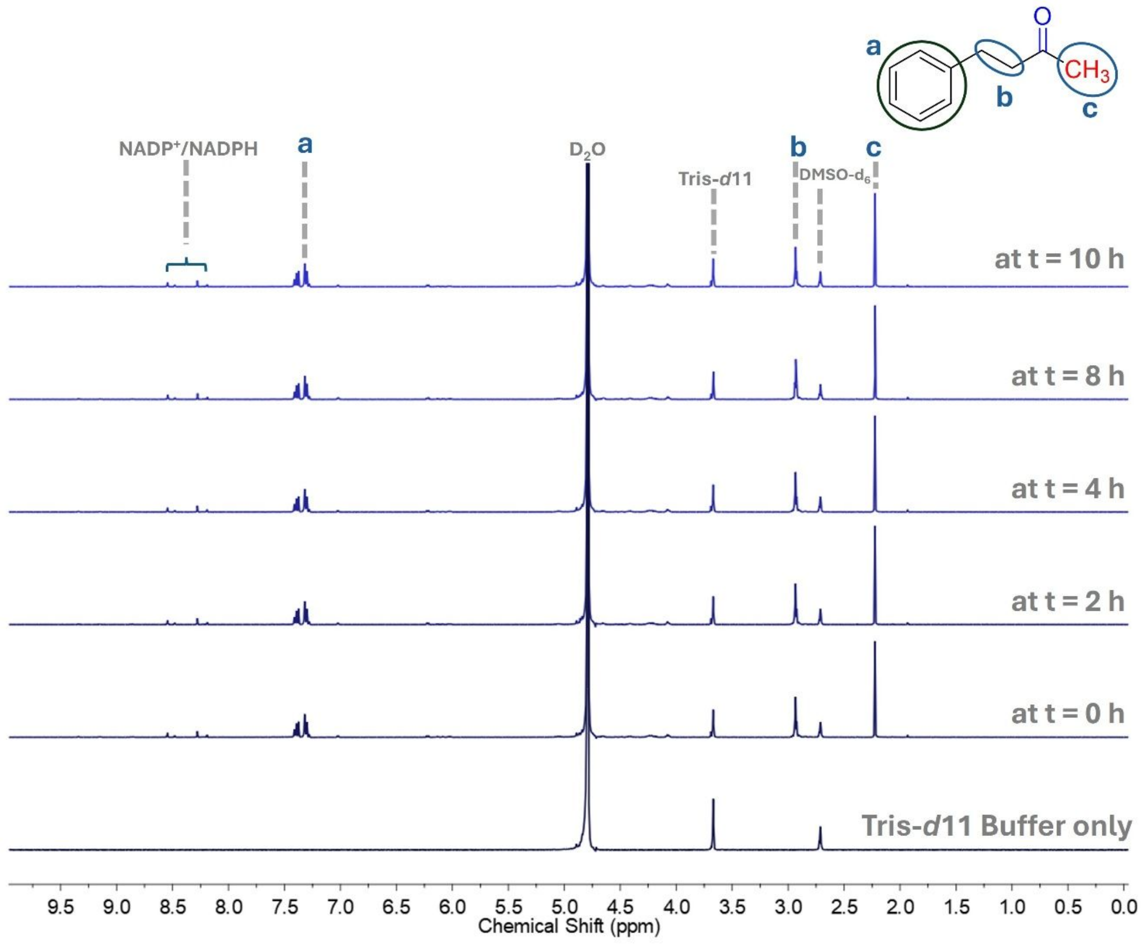

Figure A 4. Time-resolved ^{1}H NMR spectra evaluating potential background hydrogen–deuterium exchange of 4-phenyl-2-butanone in the reaction medium. ^{1}H NMR spectra were recorded at 0, 2, 4, 8, and 10 h for **1** incubated in 100 mM Tris-d_{11}–DCl buffer (pD 9) containing 5% DMSO and nicotinamide cofactors (100 µM $NADP^+$ and NADPH) under reaction conditions in the absence of applied electrolysis. Signals corresponding to the aromatic protons (a), β-methylene protons adjacent to the carbonyl group (b), and terminal methyl group (c) remain unchanged in chemical shift and multiplicity over time, indicating no detectable hydrogen–deuterium exchange at the β-position of the ketone under these conditions. The spectrum of Tris-d_{11} buffer alone is shown for reference.

13.2 Hydrogen–deuterium exchange under DC paired electrolysis (Same electrodes size and catalysts loading)

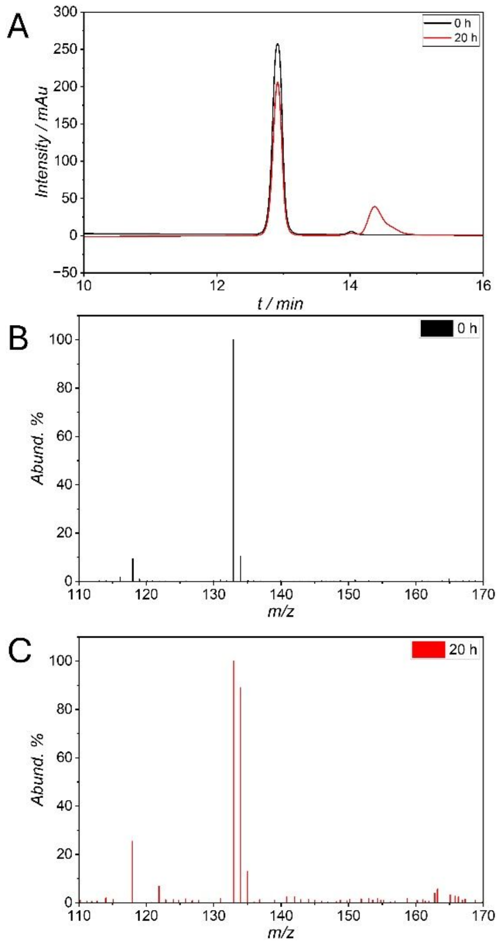

Figure A5. LC–MS analysis of hydrogen–deuterium exchange under DC paired electrolysis using identical electrodes and catalyst loadings. (A) LC traces recorded before electrolysis (0 h, black) and after 20 h of DC paired electrolysis (red), showing the formation of the deuterated alcohol. (B) Mass spectrum extracted at the retention time of the substrate prior to electrolysis, displaying the unlabeled isotopologue. (C) Mass spectrum extracted at the same retention time after 20 h of DC paired electrolysis, showing the appearance of the +1 Da isotopologue consistent with H–D exchange. Electrolysis was conducted in Tris–DCl buffer (pD 9) at 0 V vs SHE, using electrodes of identical geometric area and identical loadings of FNR and ADH at the anode and cathode.

13.3 Hydrogen–deuterium exchange under DC paired electrolysis (CE catalysts loading and size four times larger than WE)

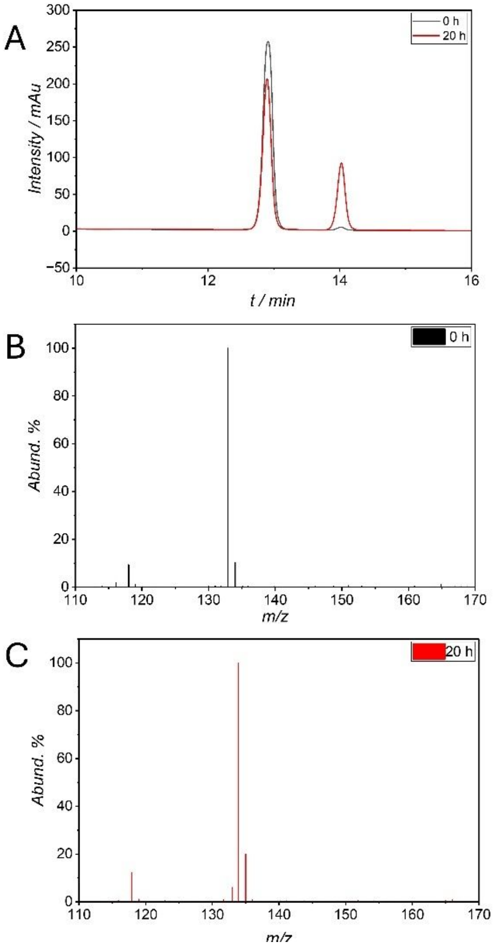

Figure A6. LC–MS analysis of hydrogen–deuterium exchange under DC paired electrolysis using CE catalysts loading and size four times larger than WE. (A) LC traces recorded before electrolysis (0 h, black) and after 20 h of DC paired electrolysis (red), showing the formation of the deuterated alcohol. (B) Mass spectrum extracted at the retention time of the substrate prior to electrolysis, displaying the unlabeled isotopologue. (C) Mass spectrum extracted at the same retention time after 20 h of DC paired electrolysis, showing the appearance of the +1 Da isotopologue consistent with H–D exchange. Electrolysis was conducted in Tris–DCl buffer (pD 9) at 0 V vs SHE.

13.4 AC operating conditions for electroenzymatic HIE (Table in Figure 5 in the manuscript)

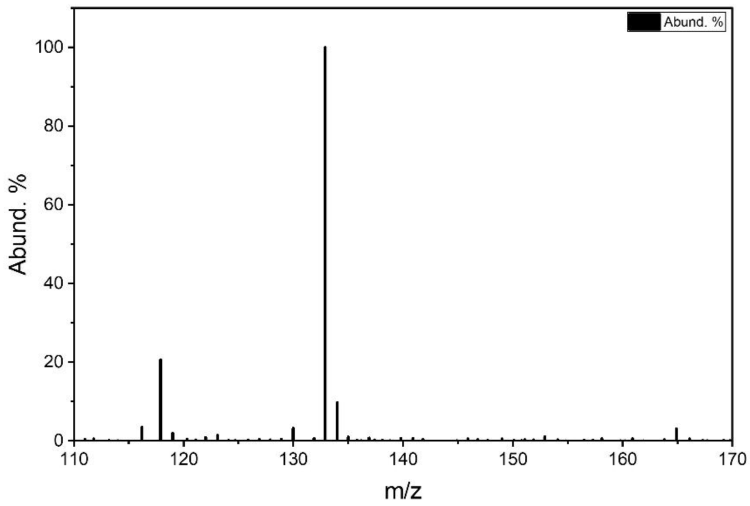

Figure A7. Mass spectrum recorded of 1′ at t_0 prior to AC electrolysis during parameter optimization. The spectrum corresponds to the untreated substrate before application of an alternating potential and serves as the reference for subsequent hydrogen–deuterium exchange analysis. Relative abundances (%) of detected ions are shown, confirming the absence of deuterium incorporation at the initial time point.

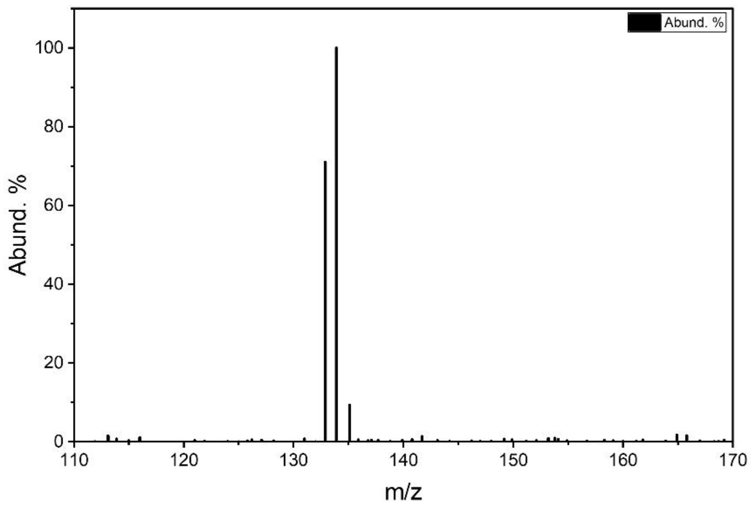

Figure A8. Mass spectrum of 1′ recorded after 1 h of AC electrolysis under optimized conditions (0.4 V amplitude, 50 mHz) in Tris–DCl buffer (100 mM, pD 9; 200 μL).

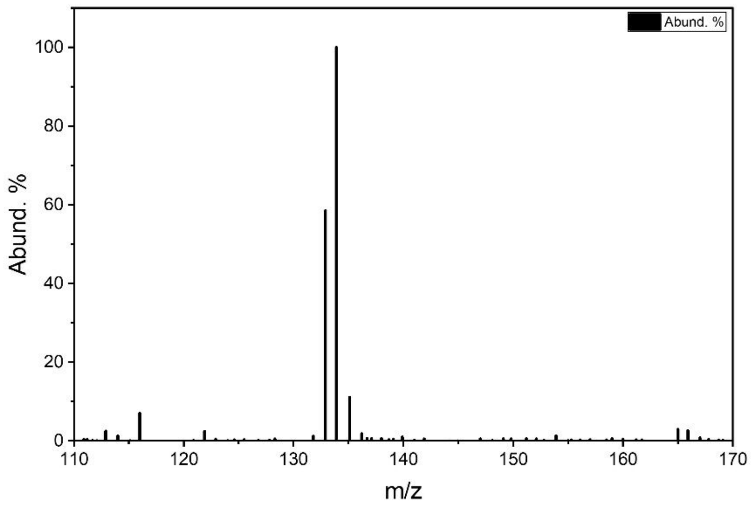

Figure A9. Mass spectrum of 1′ recorded after 1 h of AC electrolysis under optimized conditions (0.5 V amplitude, 50 mHz) in Tris–DCl buffer (100 mM, pD 9; 200 μL).

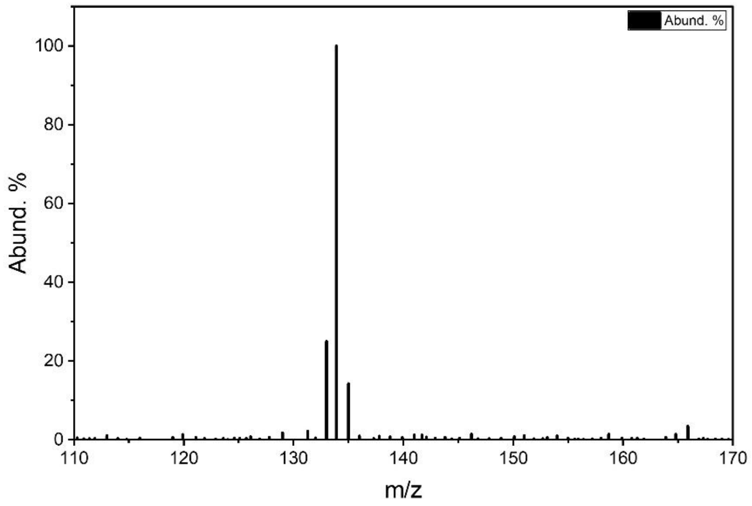

Figure A10. Mass spectrum of 1′ recorded after 1 h of AC electrolysis under optimized conditions (0.6 V amplitude, 50 mHz) in Tris–DCl buffer (100 mM, pD 9; 200 μL).

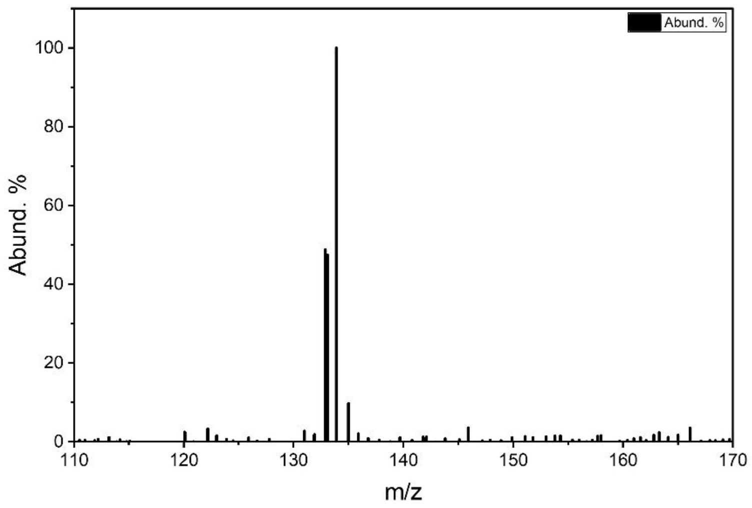

Figure A11. Mass spectrum of 1′ recorded after 1 h of AC electrolysis under optimized conditions (0.7 V amplitude, 50 mHz) in Tris–DCl buffer (100 mM, pD 9; 200 μL).

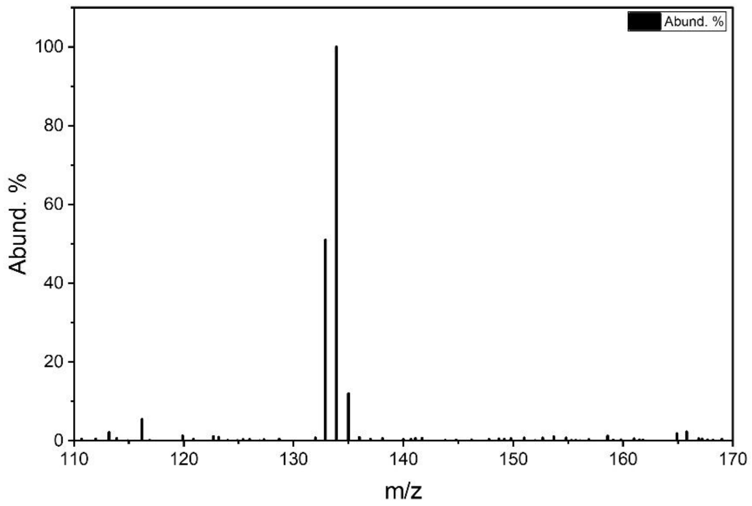

Figure A12. Mass spectrum of 1′ recorded after 1 h of AC electrolysis under optimized conditions (0.6 V amplitude, 25 mHz) in Tris–DCl buffer (100 mM, pD 9; 200 μL).

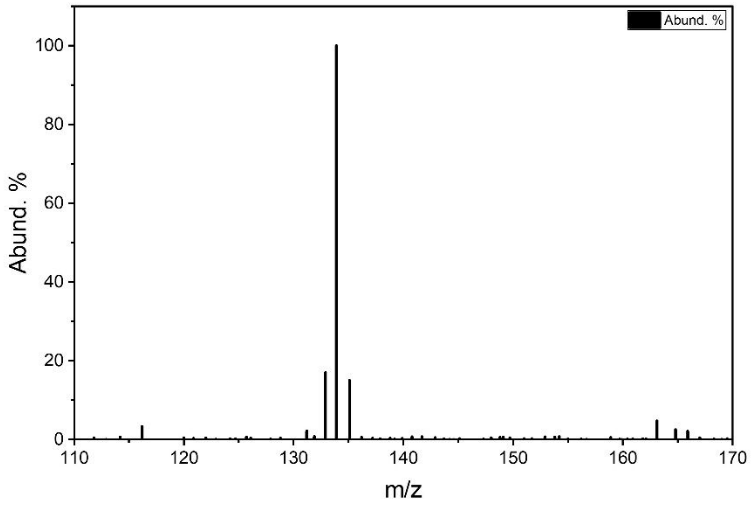

Figure A13. Mass spectrum of 1′ recorded after 1 h of AC electrolysis under optimized conditions (0.6 V amplitude, 100 mHz) in Tris–DCl buffer (100 mM, pD 9; 200 μL).

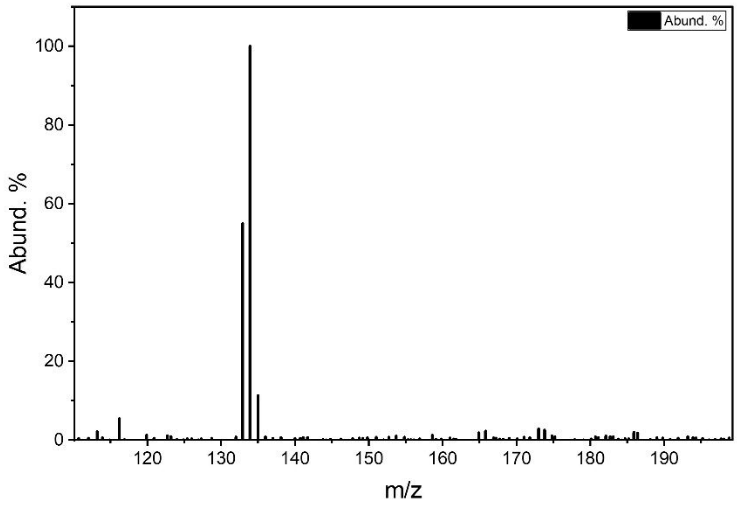

Figure A14. Mass spectrum of 1′ recorded after 1 h of AC electrolysis under optimized conditions (0.6 V amplitude, 150 mHz) in Tris–DCl buffer (100 mM, pD 9; 200 μL).

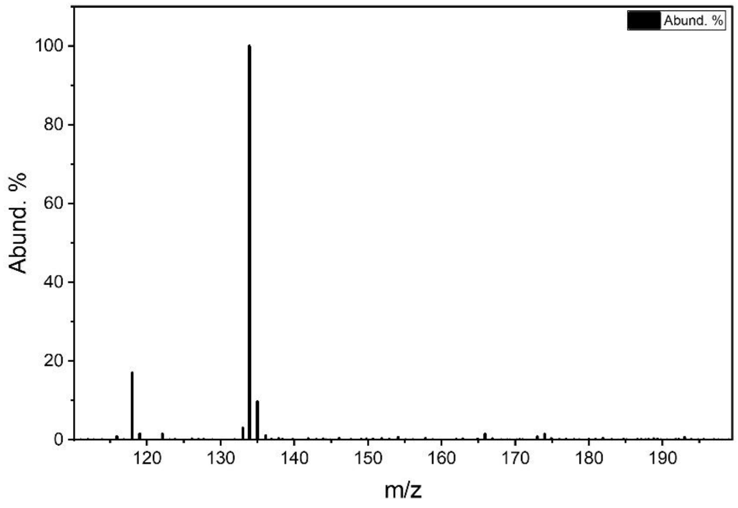

Figure A15. Mass spectrum of 1′ recorded after 1.5 h of AC electrolysis under optimized conditions (0.6 V amplitude, 150 mHz) in Tris–DCl buffer (100 mM, pD 9; 200 µL).

13.5 Preparative scale electroenzymatic HIE

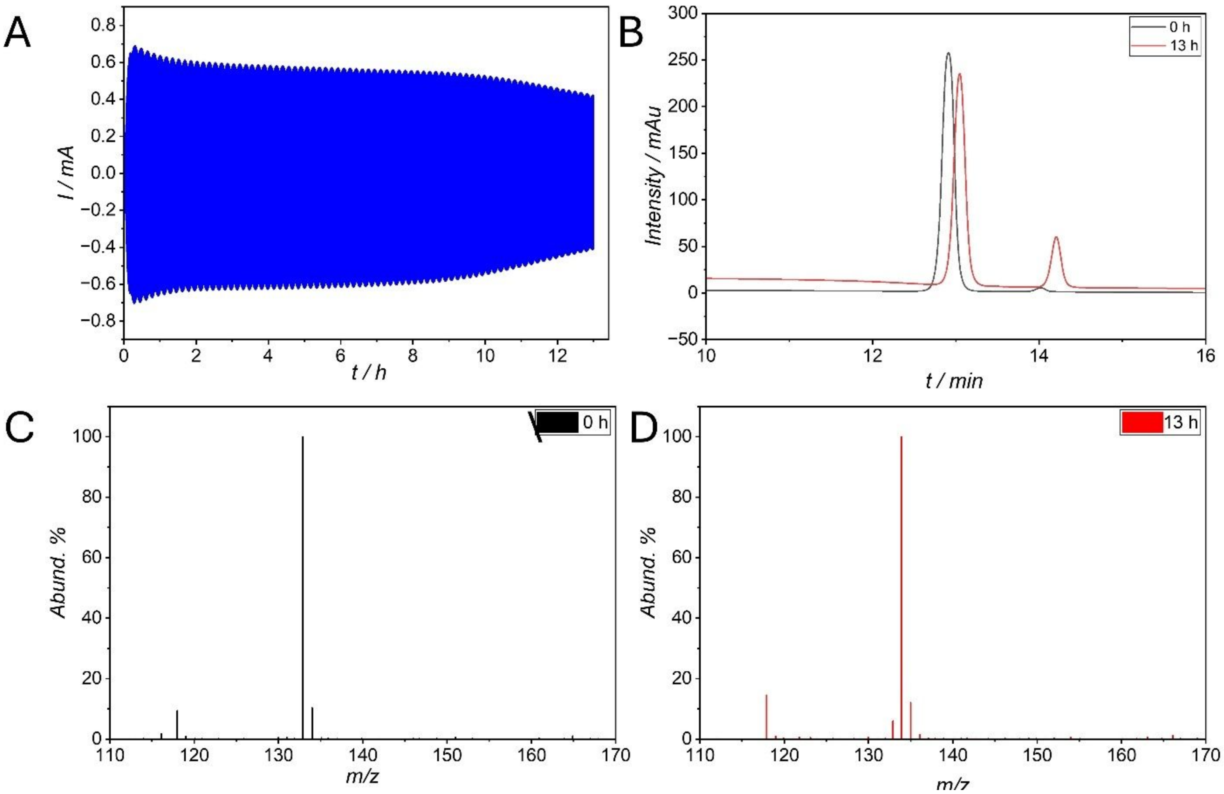

Figure A16. Preparative-scale AC electrolysis for electroenzymatic H–D exchang with FNR and ADH LK. (A) Chronoamperometric trace recorded during preparative AC electrolysis (100 mHz, 0.6 V vs CE) over 13 h. (B) LC traces acquired before electrolysis (0 h, black) and after electrolysis (red). (C) Mass spectrum extracted at the retention time of the substrate prior to electrolysis (0 h), displaying the unlabeled isotopologue. (D) Mass spectrum extracted at the same retention time after 13 h of AC electrolysis, showing the appearance of the +1 Da isotopologue consistent with hydrogen–deuterium exchange. All experiments were conducted at preparative scale (3 mL) in Tris–DCl buffer (100 mM, pD 9) under AC electrolysis conditions.

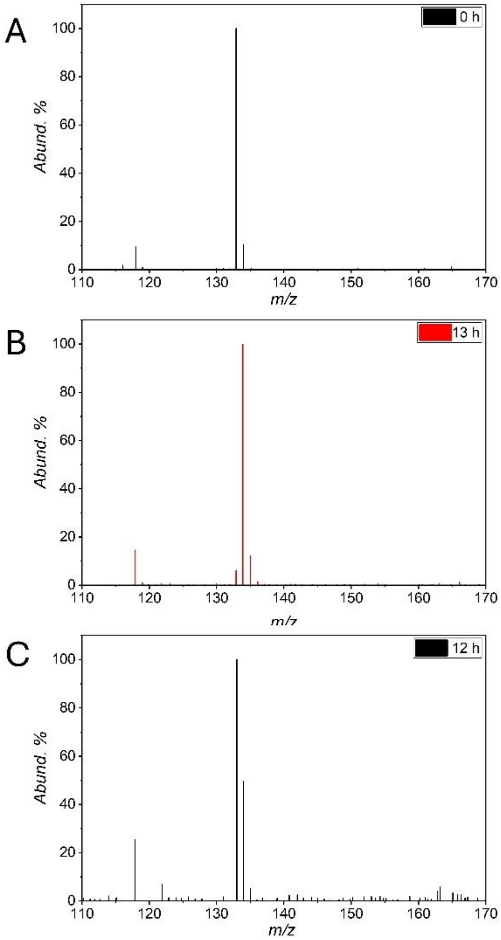

Figure A17. Comparison of hydrogen–deuterium exchange under AC and DC electrolysis at matched reaction times. (A) Mass spectrum of the substrate prior to electrolysis (0 h), showing the unlabeled isotopologue. (B) Mass spectrum recorded after 13 h of AC electrolysis, displaying a dominant +1 Da isotopologue indicative of efficient H–D exchange. (C) Mass spectrum recorded after 12 h of DC electrolysis under otherwise identical conditions, showing markedly lower deuterium incorporation. All experiments were conducted under the same conditions (Tris–DCl buffer, 100 mM, pD 9).

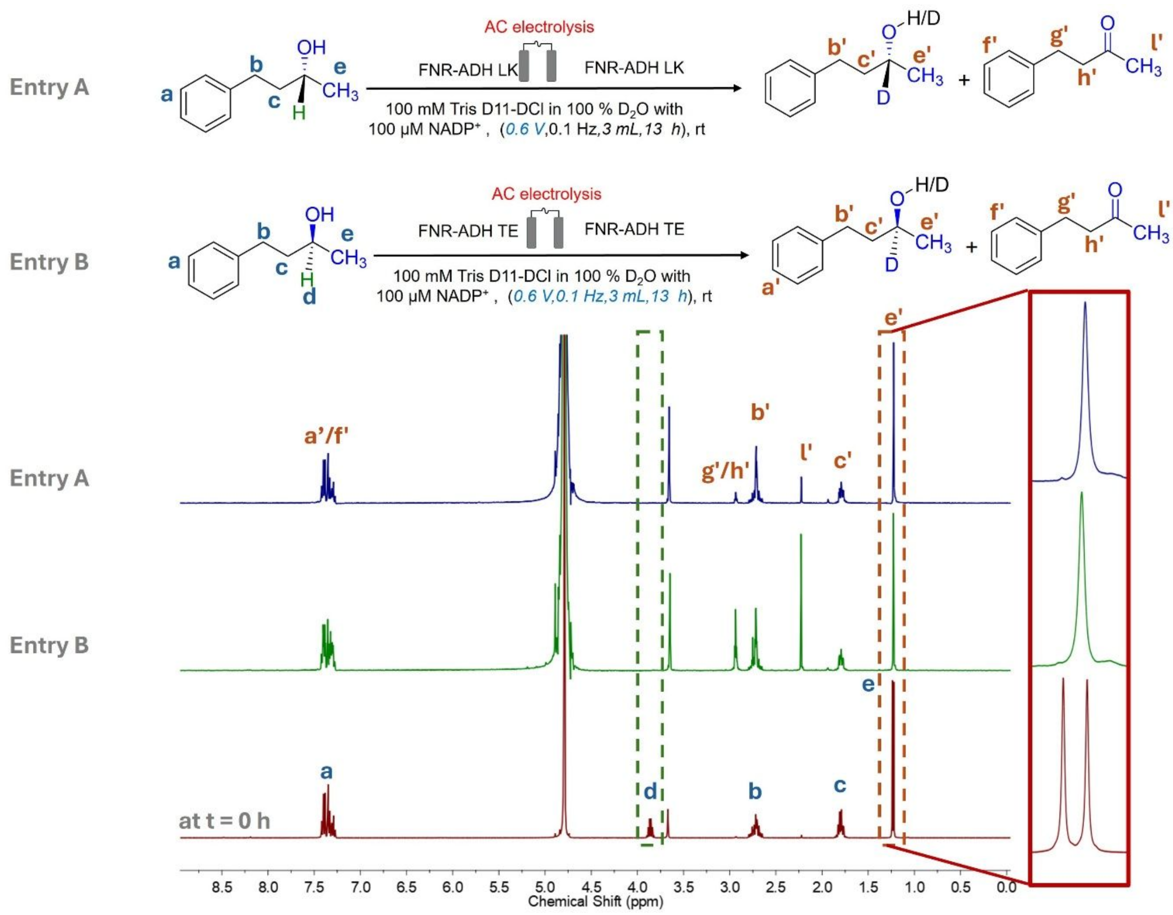

Figure A 18. **Entry A: Electroenzymatic** HIE at a stereogenic alcohol center under AC electrolysis and corresponding ^{1}H NMR analysis. HIE of 5mM **1′**catalyzed by **FNR** and **ADH LK** immobilized on both electrodes under AC electrolysis (0.6 V amplitude, 0.1 Hz).

Entry B: HIE of 5 mM **2′**alcohol catalyzed by **FNR** and **ADH TE** immobilized on both electrodes under identical AC electrolysis conditions. Reversible enzymatic oxidation–reduction cycles enable incorporation of deuterium at the stereogenic center while preserving the original stereochemistry.

Reactions were performed in 100 mM Tris-d11–DCl buffer (pD 9) containing 100 µM $NADP^+$ in 100% D_2O (3 mL, 13 h, room temperature). The spectrum at **t = 0 h** corresponds to the starting material prior to electrolysis. Spectra recorded after electrolysis for Entry A and Entry B show disappearance of the signal corresponding to the stereogenic proton and formation of the deuterated product. The highlighted region shows the methyl resonance, where collapse of the doublet to a singlet is consistent with incorporation of deuterium at the stereogenic center (>95 % D).

13.6 AC electrolysis scope

13.6.1 LC-MS characterization

13.6.1.1 LC–MS characterization of deuterated (R)-p-nitro-4-phenyl-2-butanol (A_2)

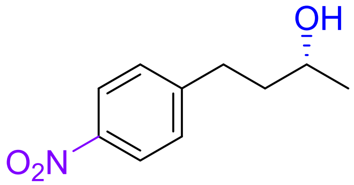

Table A_2. LC–MS analysis method for p-nitro-4-phenyl-2-butanol

Parameter	Setting
Mobile phase A	H_2O + 0.1% formic acid
Mobile phase B	ACN + 0.1% formic acid
Flow rate / mL·min^{-1}	0.40 mL·min^{-1}
Column temperature / °C	40
Injection volume / µL	5 µL
Gradient	0–12 min, 75:25 (A:B); 12–16 min, linear to 25:75; 16–18 min, 75:25; 18–20 min, re-equilibration to 75:25
DAD / nm	210
Ionization	ESI$^+$
Scan range / m/z	160–200

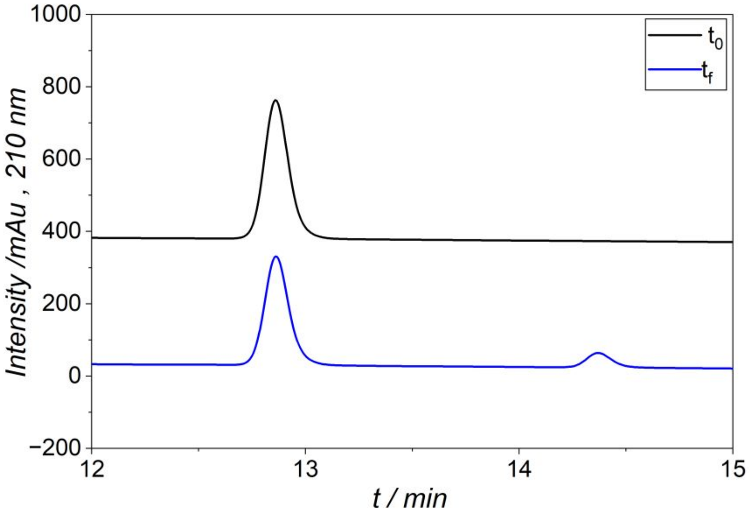

Figure A19. LC–DAD chromatograms (210 nm) of (R)-p-nitro-4-phenyl-2-butanol recorded at the start (t_0, black) and after electrolysis (t_f, blue). The parent alcohol elutes at t□ ≈ 12.8 min, while the ketone formed during HIE elutes at t□ ≈ 14.4 min.

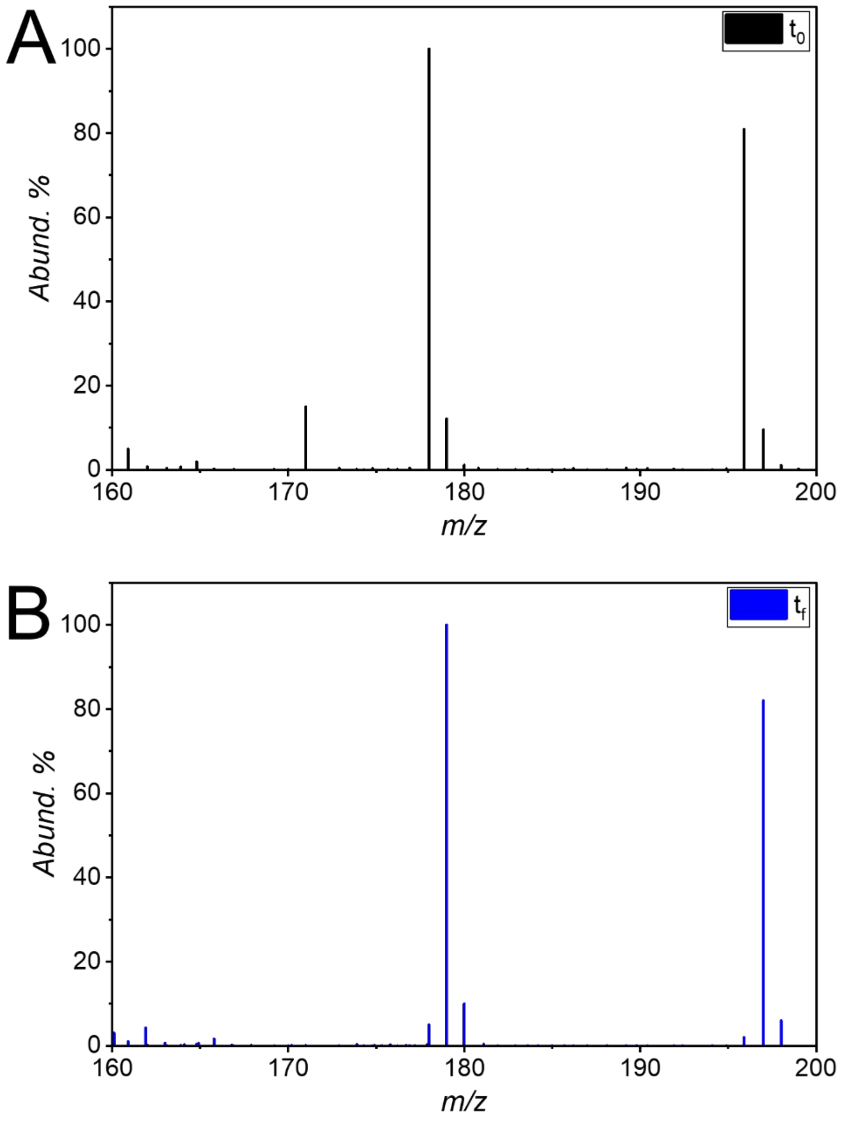

Figure A20. ESI(+) mass spectrum of (R)-p-nitro-4-phenyl-2-butanol (A) before and (B) after HIE, showing a +1 Da shift of the molecular ion consistent with mono-deuteration.

13.6.1.2 LC–MS characterization of deuterated (*R*)-phenylpropan-2-ol (A_3)

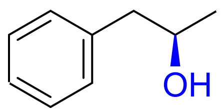

Table A1. LC–MS analysis method for (*R*)-phenylpropan-2-ol

Parameter	Setting
Mobile phase A	H_2O + 0.1% formic acid
Mobile phase B	ACN + 0.1% formic acid
Flow rate / mL·min^{-1}	0.40 mL·min^{-1}
Column temperature / °C	35
Injection volume / µL	5 µL
Gradient	0–8 min, 80:20 (A:B); 8–12 min, linear to 20:80; 12–14 min, 80:20; 14–16 min, re-equilibration to 80:20
DAD / nm	210
Ionization	ESI$^+$
Scan range / m/z	90-140

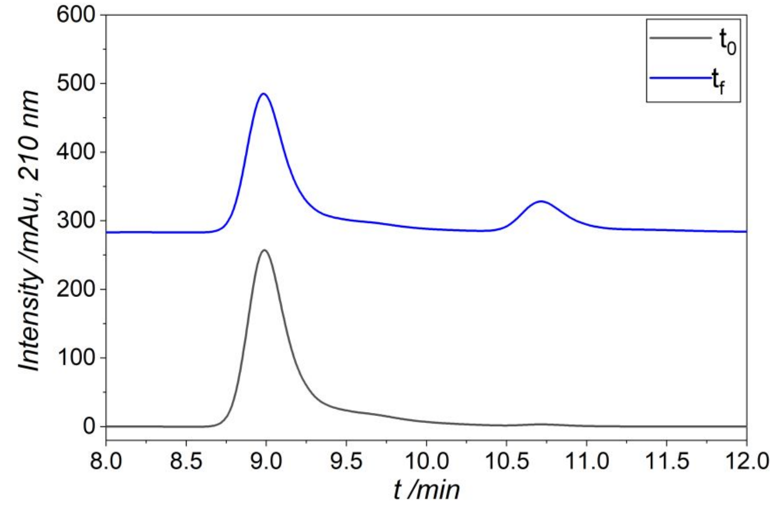

Figure A21. LC–DAD chromatograms (210 nm) of (R)-phenylpropan-2-ol recorded at the start (t_0, black) and after electrolysis (t_f, blue). The alcohol elutes at t□ ≈ 9 min, while the ketone formed during HIE elutes at t□ ≈ 10.5min.

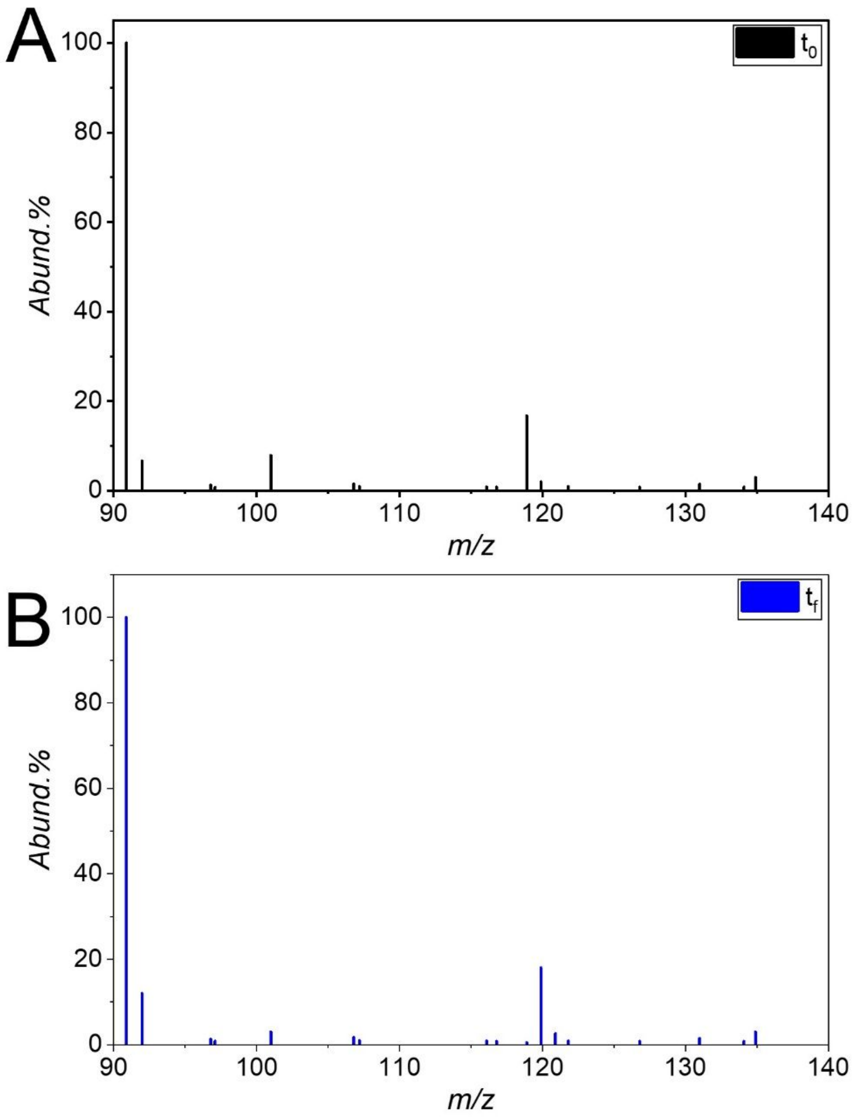

Figure A22. ESI(+) mass spectrum of (R)-phenylpropan-2-ol (A) before and (B) after HIE, showing a +1 Da shift of the molecular ion consistent with mono-deuteration.

13.6.1.3 LC–MS characterization of deuterated (*R*)-(+)-1-Phenylethanol (A_4)

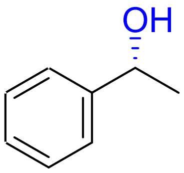

Table A2. LC–MS analysis method for (*R*)-(+)-1-Phenylethanol (A_4).

Parameter	Setting
Mobile phase A	H_2O + 0.1% formic acid
Mobile phase B	MeOH + 0.1% formic acid
Flow rate / mL·min^{-1}	0.30 mL·min^{-1}
Column temperature / °C	35
Injection volume / µL	5 µL
Gradient	0–16 min, 60:40 (A:B); 16–27 min, 40:60 (A:B); 27–28 min, 40:60 (A:B); 28–29 min, 60:40 (A:B); 29–30 min, 60:40 (A:B)
DAD / nm	210
Ionization	ESI$^+$
Scan range / m/z	70-125

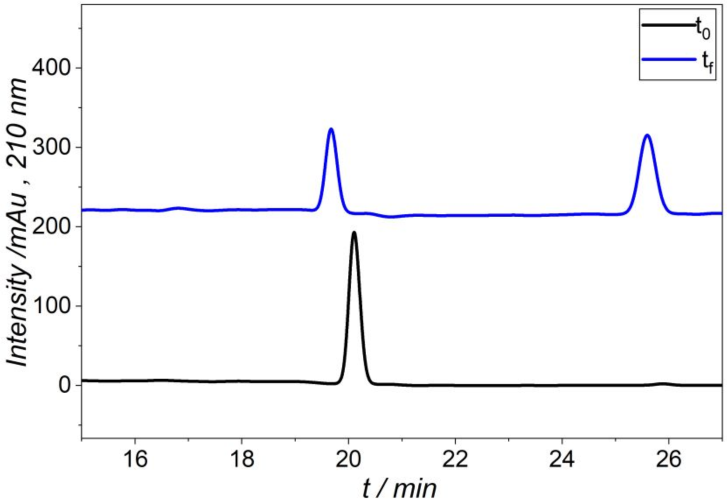

Figure A23. LC–DAD chromatograms (210 nm) of (*R*)-(+)-1-Phenylethanol recorded at the start (t_0, black) and after electrolysis (t_f, blue). The alcohol elutes at t□ ≈ 20 min, while the ketone formed during HIE elutes at t□ ≈ 25.5min.

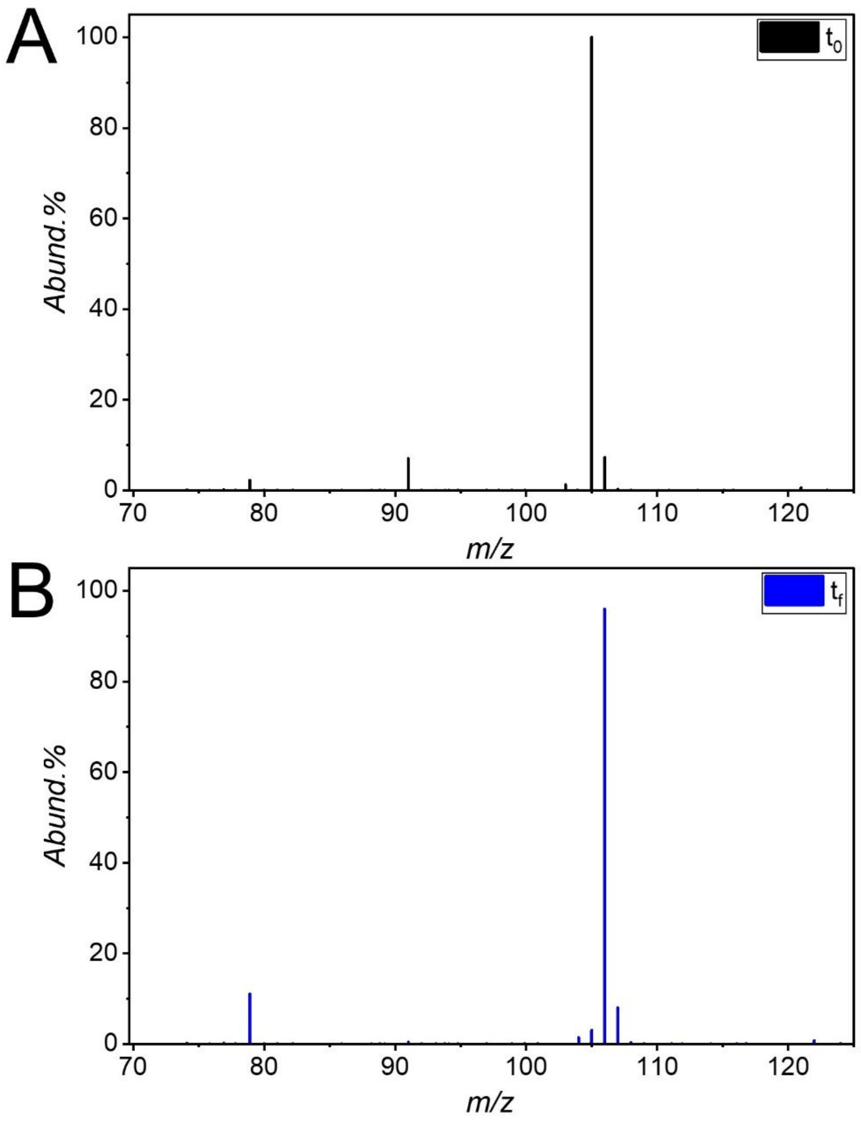

Figure A24. ESI(+) mass spectrum of (*R*)-(+)-1-Phenylethanol (A) before and (B) after HIE, showing a +1 Da shift of the molecular ion consistent with mono-deuteration.

13.6.1.4 LC–MS characterization of deuterated (*R*)-p-tolyl ethanol (A_5)

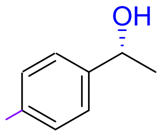

Table A3. LC–MS analysis method for (R)-p-tolyl ethanol.

Parameter	Setting
Mobile phase A	H_2O + 0.1% formic acid
Mobile phase B	MeOH + 0.1% formic acid
Flow rate / mL·min^{-1}	0.30 mL·min^{-1}
Column temperature / °C	35
Injection volume / µL	5 µL
Gradient	0–16 min, 60:40 (A:B); 16–37 min, 40:60 (A:B); 37–38 min, 40:60 (A:B); 38–39 min, 60:40 (A:B); 39–40 min, 60:40 (A:B)
DAD / nm	210
Ionization	ESI^{+}
Scan range / m/z	100–130

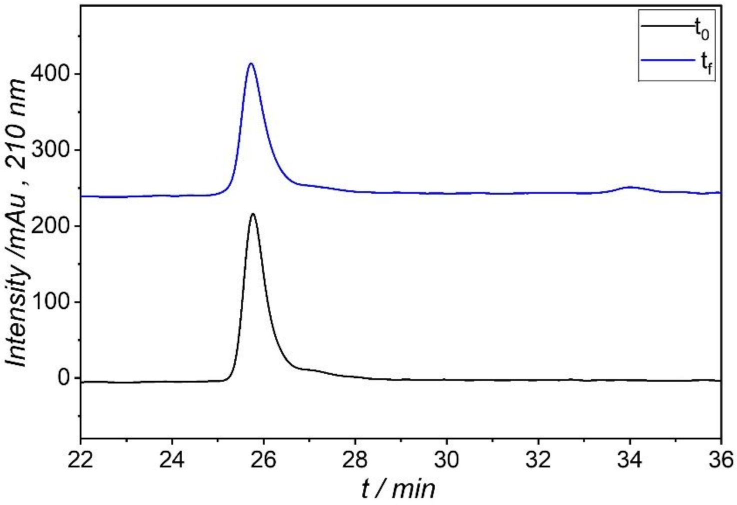

Figure A25. LC–DAD chromatograms (210 nm) of (*R*)-p-tolyl ethanol recorded at the start (t_0, black) and after electrolysis (t_f, blue). The alcohol elutes at t□ ≈ 26 min, while the ketone formed during HIE elutes at t□ ≈ 34 min.

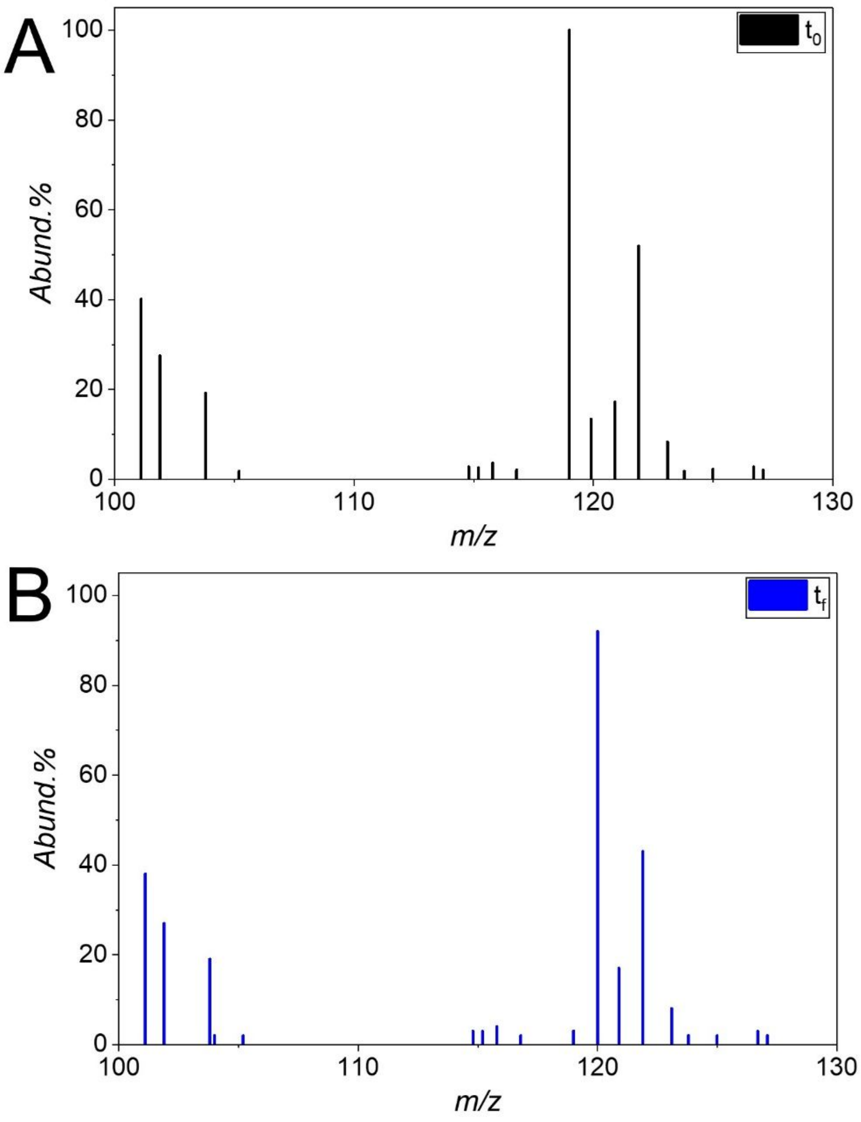

Figure A26. ESI(+) mass spectrum of (*R*)-p-tolyl ethanol (A) before and (B) after HIE, showing a +1 Da shift of the molecular ion consistent with mono-deuteration.

13.6.1.5 LC–MS characterization of deuterated chloro-substituted phenylethanols (A_6; A_7; A_8)

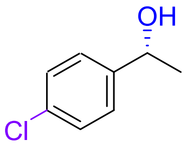

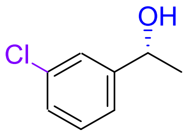

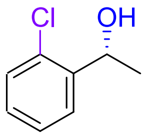

(A_6) **(A_7)** **(A_8)**

Table A4. LC–MS analysis method for chloro-substituted phenylethanols.

Parameter	Setting
Mobile phase A	H_2O + 0.1% formic acid
Mobile phase B	MeOH + 0.1% formic acid
Flow rate / mL·min⁻¹	0.30 mL·min⁻¹
Column temperature / °C	30
Injection volume / µL	4 µL
Gradient	0–16 min, 60:40 (A:B); 16–37 min, 40:60 (A:B); 37–38 min, 40:60 (A:B); 38–39 min, 60:40 (A:B); 39–40 min, 60:40 (A:B)
DAD / nm	210
Ionization	ESI^+
Scan range / m/z	100–140

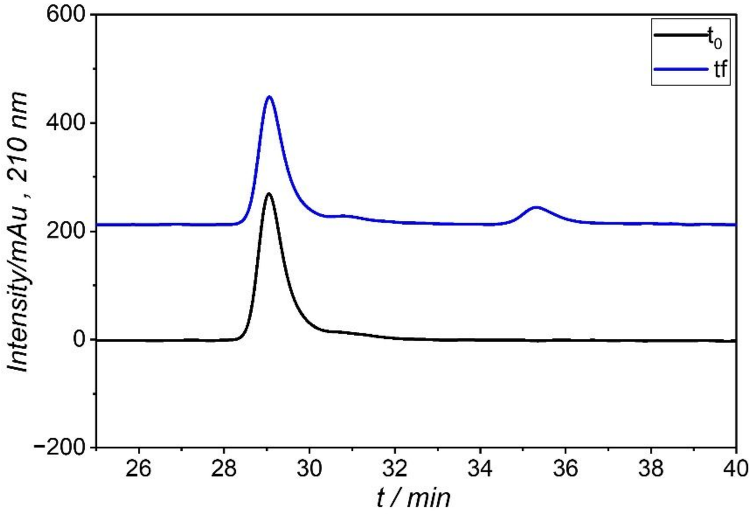

Figure A27. LC–DAD (210 nm) chromatograms of chloro-substituted phenylethanols before (t_0, black) and after electroenzymatic HIE (tf, blue). In all cases, the alcohol elutes at ~28–30 min, while the ketone late-eluting features (~35–37 min)

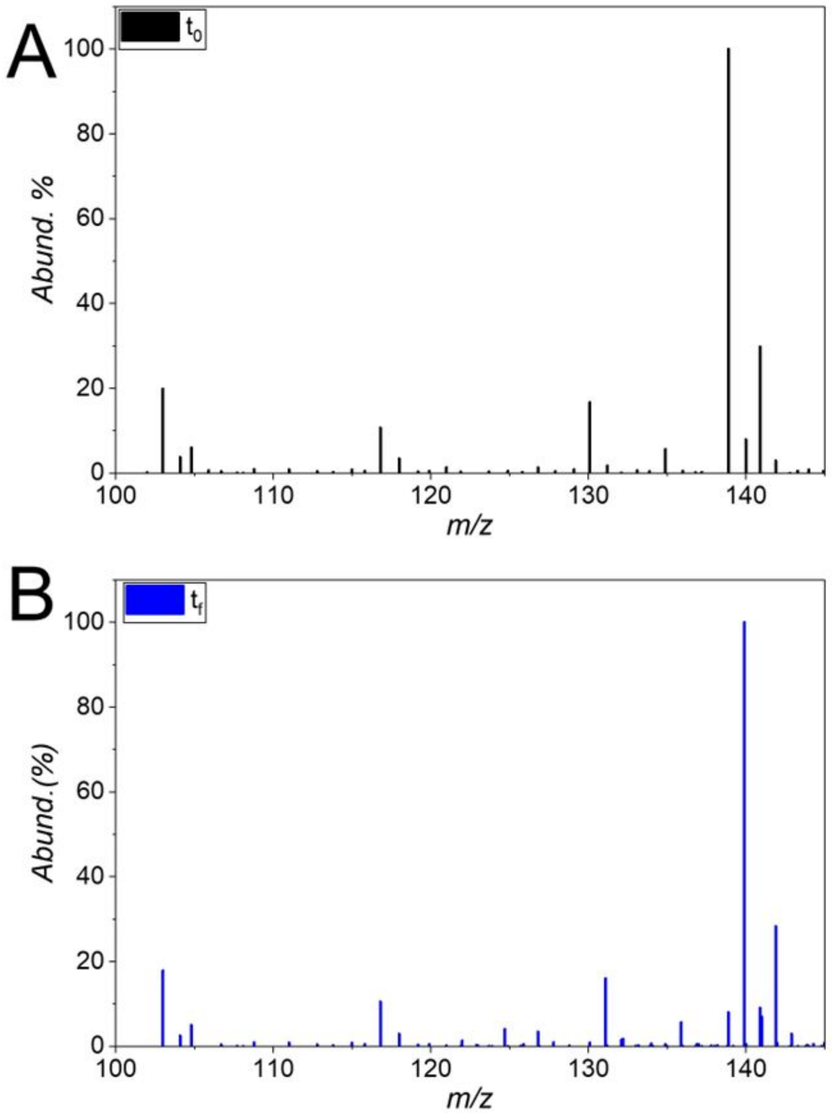

Figure A28. ESI(+) mass spectrum of (R)-chloro-substituted phenylethanols (A) before and (B) after HIE, showing a +1 Da shift of the molecular ion consistent with mono-deuteration.

13.6.1.6 LC–MS characterization of deuterated methoxyphenylethanol (A_9)

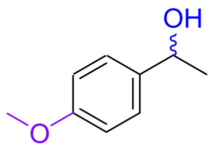

Table A5. LC–MS analysis method for methoxyphenylethanol.

Parameter	Setting
Mobile phase A	H_2O + 0.1% formic acid
Mobile phase B	MeOH + 0.1% formic acid
Flow rate / mL·min⁻¹	0.30 mL·min⁻¹
Column temperature / °C	25
Injection volume / µL	3 µL
Gradient	0–16 min, 60:40 (A:B); 16–37 min, 40:60 (A:B); 37–38 min, 40:60 (A:B); 38–39 min, 60:40 (A:B); 39–40 min, 60:40 (A:B)
DAD / nm	210
Ionization	ESI^+
Scan range / m/z	90–145

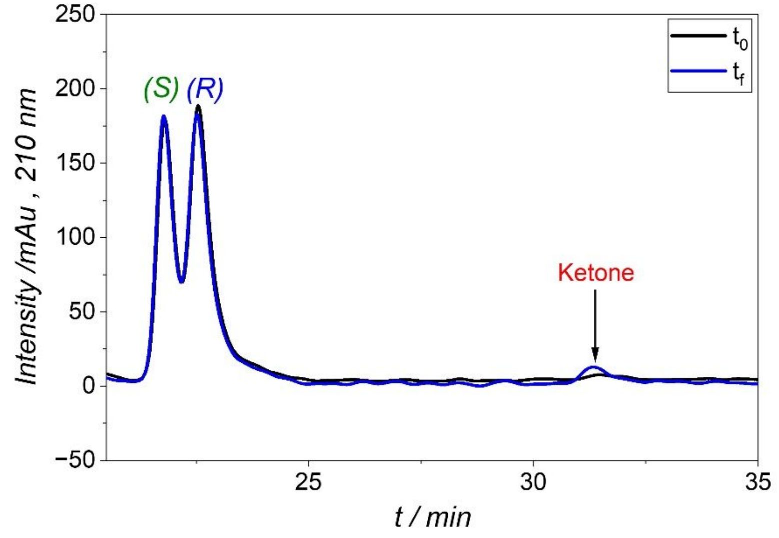

Figure A29. LC–DAD chromatograms (210 nm) of methoxyphenylethanol recorded at the start (t_0, black) and after electrolysis (t_f, blue). The alcohol elutes at t□ ≈ 22 min, while the ketone formed during HIE elutes at t□ ≈ 32.5 min.

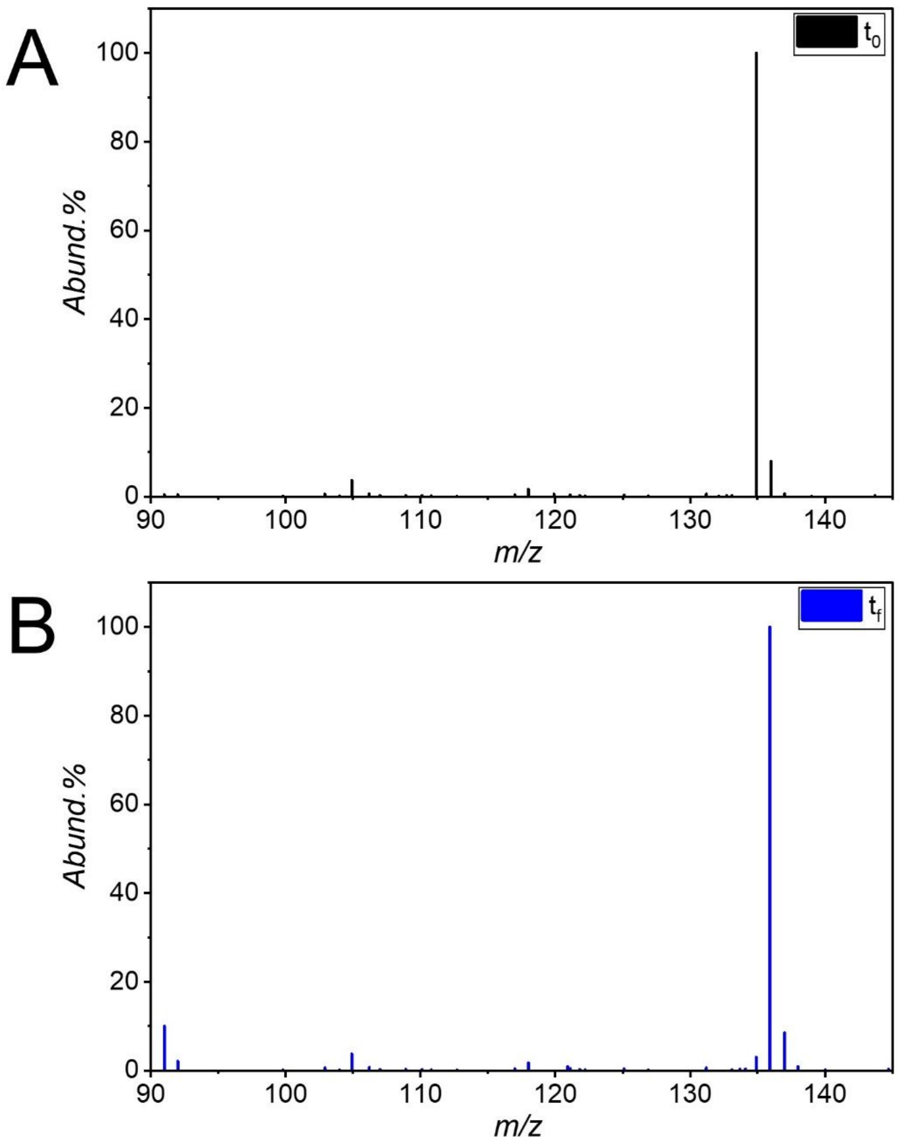

Figure A30. ESI(+) mass spectrum of (*R*)- methoxyphenylethanol (A) before and (B) after HIE, showing a +1 Da shift of the molecular ion consistent with mono-deuteration.

13.6.1.7 LC–MS characterization of deuterated nitro-substituted phenylethanols (A_{11}; A_{12})

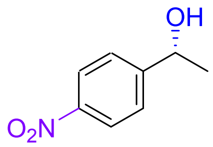

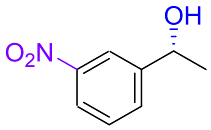

(A_{11}) **(A_{12})**

Table A6. LC–MS analysis method for nitro-substituted phenylethanols.

Parameter	Setting
Mobile phase A	H_2O + 0.1% formic acid
Mobile phase B	MeOH + 0.1% formic acid
Flow rate / $mL \cdot min^{-1}$	0.30 $mL \cdot min^{-1}$
Column temperature / °C	30
Injection volume / µL	4 µL
Gradient	0–14 min, 60:40 (A:B); 14–28 min, 40:60 (A:B); 28–29 min, 40:60 (A:B); 29–30 min, 60:40 (A:B); 30–31 min, 60:40 (A:B)
DAD / nm	210
Ionization	ESI^+
Scan range / m/z	90–175

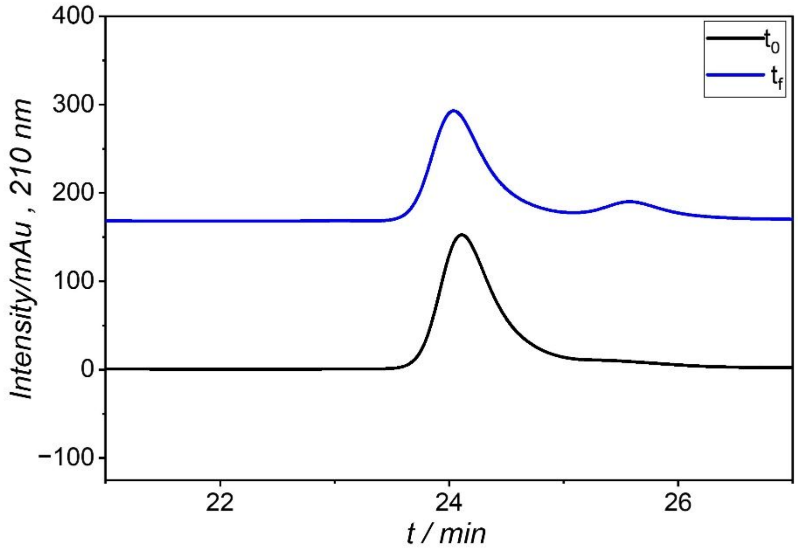

Figure A31. LC–DAD (210 nm) chromatograms of nitro-substituted phenylethanols before (t_0, black) and after electroenzymatic HIE (t_f, blue). In all cases, the alcohol elutes at ~24 min, while the ketone late-eluting features (~25.5min)

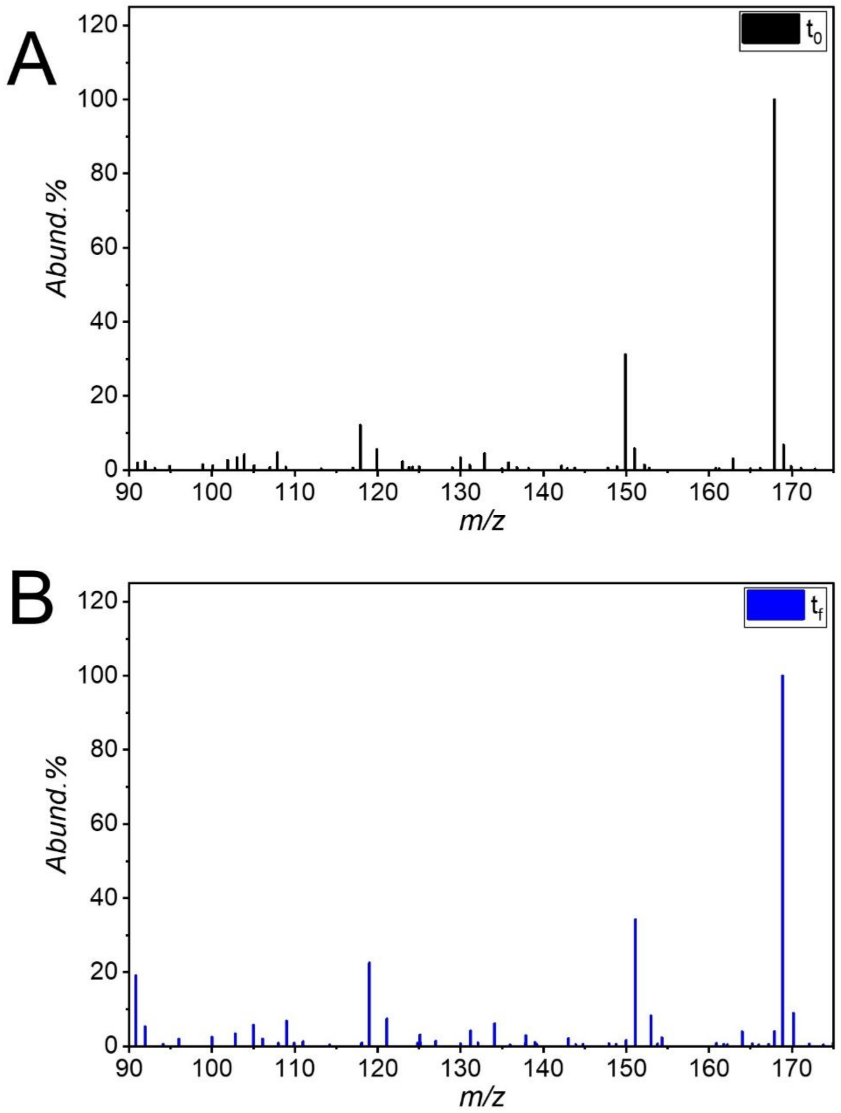

Figure A32. ESI(+) mass spectrum of (*R*)- nitro-substituted phenylethanols (A) before and (B) after HIE, showing a +1 Da shift of the molecular ion consistent with mono-deuteration.

13.6.1.8 LC–MS characterization of deuterated (*R*)-1-(2-Bromophenyl)ethanol (A_{13})

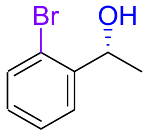

(A_{13})

Table A7. LC–MS analysis method for (*R*)-1-(2-Bromophenyl)ethanol.

Parameter	Setting
Mobile phase A	H_2O + 0.1% formic acid
Mobile phase B	MeOH + 0.1% formic acid
Flow rate / mL·min⁻¹	0.30 mL·min⁻¹
Column temperature / °C	30
Injection volume / µL	4 µL
Gradient	0–14 min, 60:40 (A:B); 14–47 min, 40:60 (A:B); 47–49 min, 40:60 (A:B); 49–50 min, 60:40 (A:B); 50–51 min, 60:40 (A:B)
DAD / nm	214
Ionization	ESI^+
Scan range / m/z	90–190

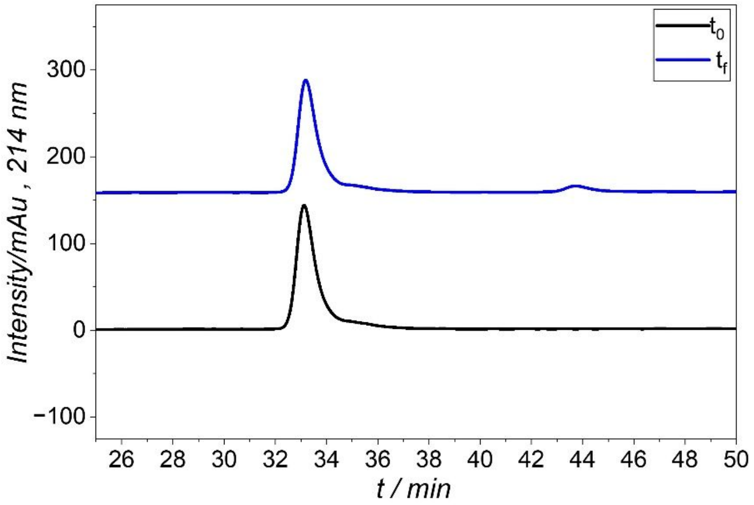

Figure A33. LC–DAD (210 nm) chromatograms of (*R*)-1-(2-Bromophenyl)ethanol before (t_0, black) and after electroenzymatic HIE (t_f, blue). The alcohol elutes at ~33 min, while the ketone late-eluting features (~44 min)

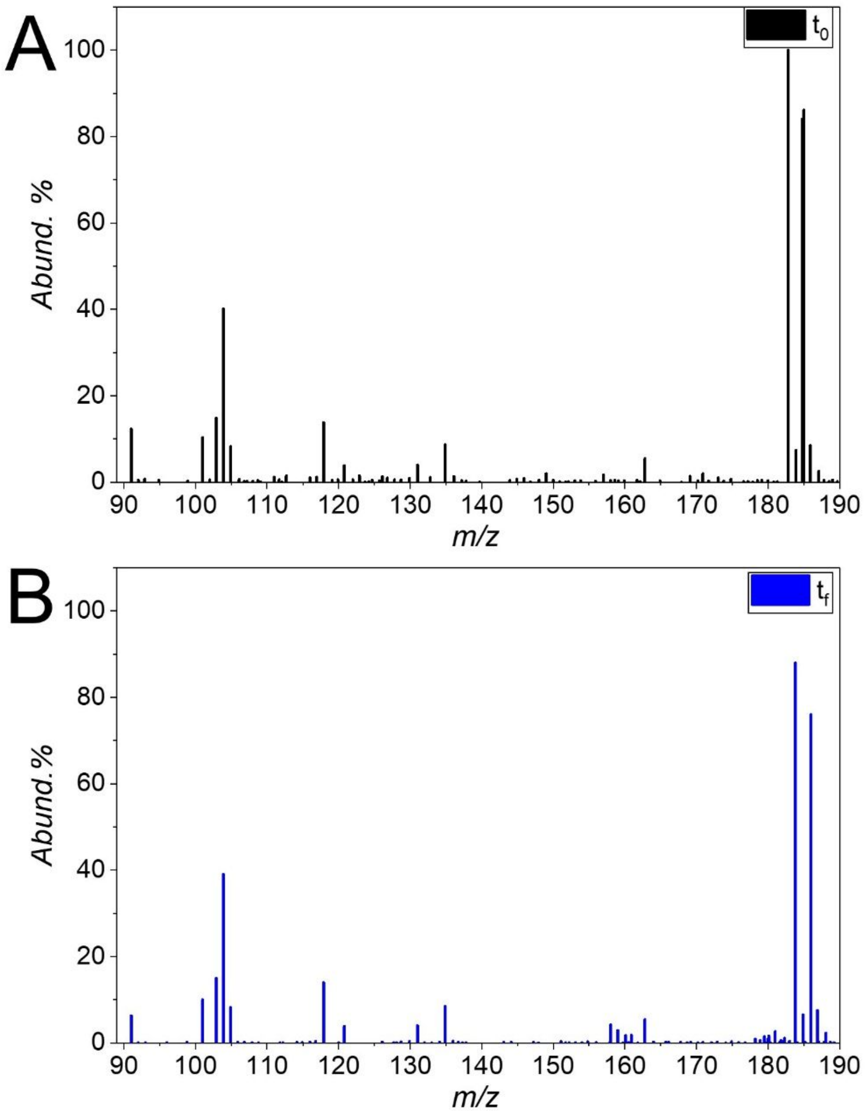

Figure A34. ESI(+) mass spectrum of (*R*)-1-(2-Bromophenyl) ethanol (A) before and (B) after HIE, showing a +1 Da shift of the molecular ion consistent with mono-deuteration.

13.6.1.9 LC–MS characterization of deuterated 1-phenylhexanol (B_1)

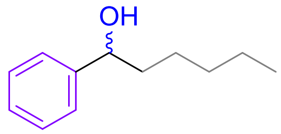

(B_1)

Table A8. LC–MS analysis method for 1-phenylhexanol.

Parameter	Setting
Mobile phase A	H_2O + 0.1% formic acid
Mobile phase B	ACN + 0.1% formic acid
Flow rate / mL·min⁻¹	0.40 mL·min⁻¹
Column temperature / °C	30
Injection volume / µL	4 µL
Gradient	0–12 min, 75:25(A:B); 12–25 min, 25:75; 25–26 min, 75:25; 26–27 min, 75:25
DAD / nm	210
Ionization	ESI^+
Scan range / m/z	110–170

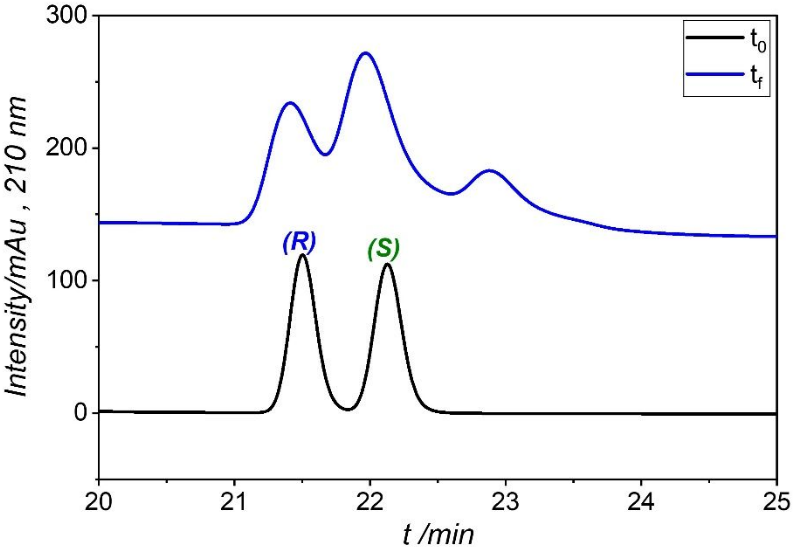

Figure A35. LC–DAD chromatograms (210 nm) of 1-phenylhexanol recorded at the start (t_0, black) and after electrolysis (t_f, blue). The parent alcohol elutes at t□ ≈ 21.5 min, while the ketone formed during HIE elutes at t□ ≈ 23 min.

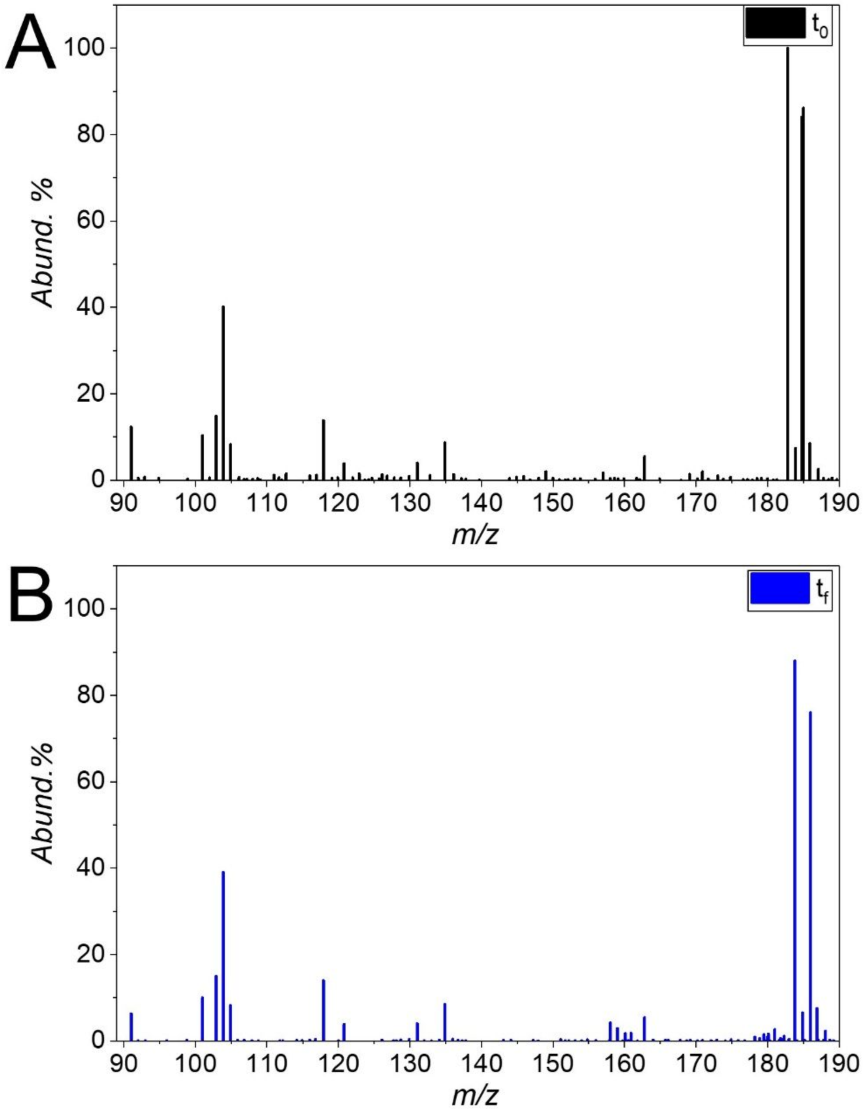

Figure A36. ESI(+) mass spectrum of (*R*)-1-phenylhexanol (A) before and (B) after HIE, showing a +1 Da shift of the molecular ion consistent with mono-deuteration.

13.6.1.10 LC–MS characterization of deuterated 1-(5-Chlorothiophen-3-yl)ethanol (C_1)

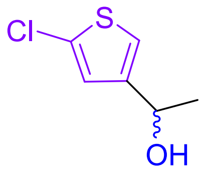

Table A9. LC–MS analysis method for 1-(5-Chlorothiophen-3-yl)ethanol.

Parameter	Setting
Mobile phase A	H_2O + 0.1% formic acid
Mobile phase B	MeOH + 0.1% formic acid
Flow rate / mL·min⁻¹	0.40 mL·min⁻¹
Column temperature / °C	25
Injection volume / µL	5 µL
Gradient	0–10 min, 85:15 (A:B); 10–20 min, linear to 70:30 (A:B); 20–28 min, linear to 60:40 (A:B); 28–30 min, 60:40 (A:B); 30–32 min, linear to 85:15 (A:B); 32–38 min, 85:15 (A:B).
DAD / nm	210
Ionization	ESI^+
Scan range / m/z	80–170

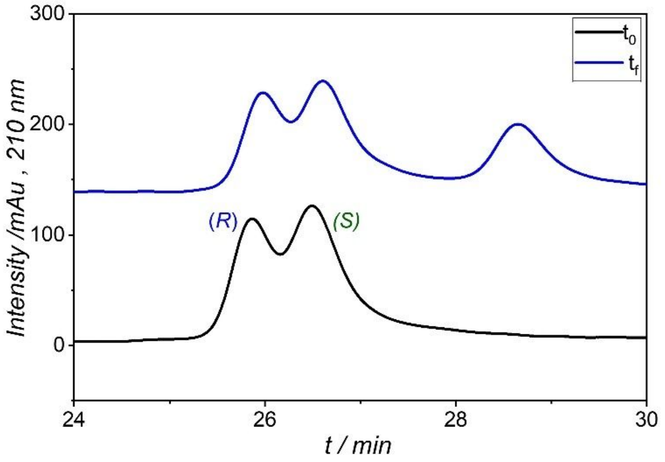

Figure A37. LC–DAD chromatograms (210 nm) of 1-(5-Chlorothiophen-3-yl) ethanol recorded at the start (t_0, black) and after electrolysis (t_f, blue). The alcohol elutes at t□ ≈ 26 min, while the ketone formed during HIE elutes at t□ ≈ 29 min.

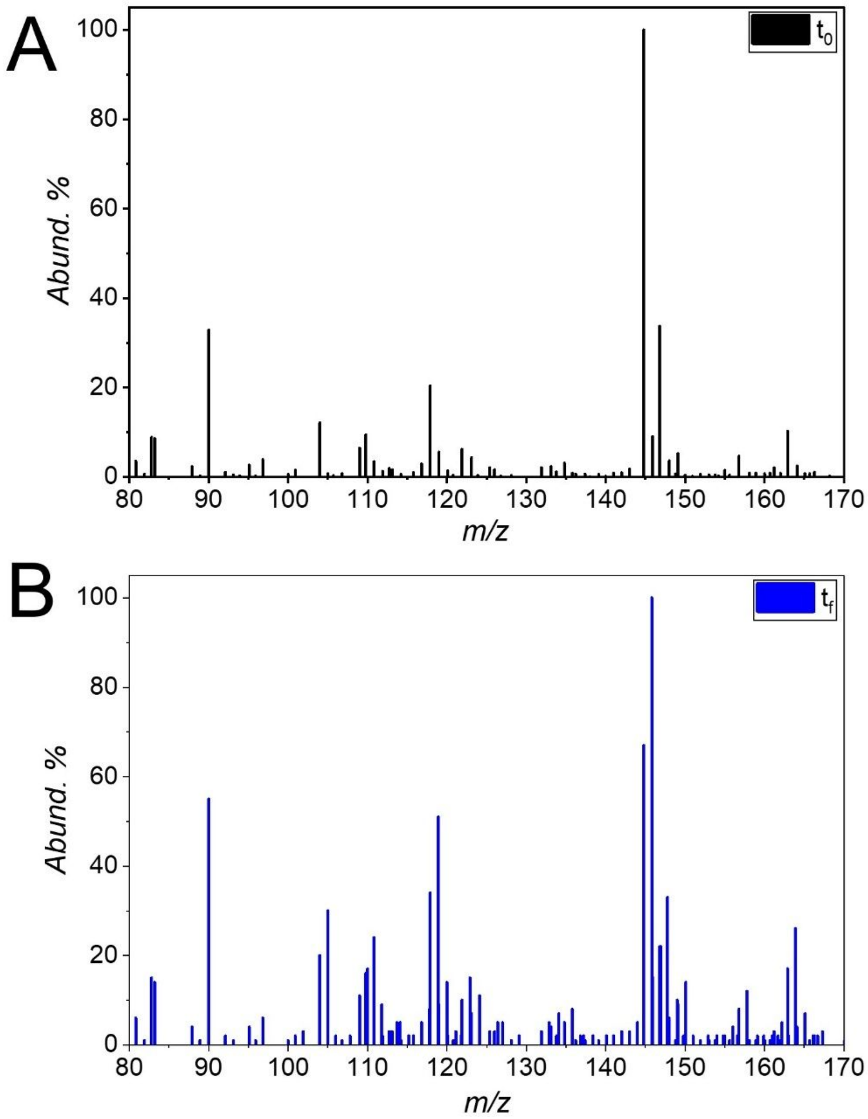

Figure A38. Mass spectrum of (*R*)-1-phenylhexanol. ESI(+) mass spectrum of (*R*)-1-(5-Chlorothiophen-3-yl)ethanol (A) before and (B) after HIE, showing a +1 Da shift of the molecular ion consistent with mono-deuteration.

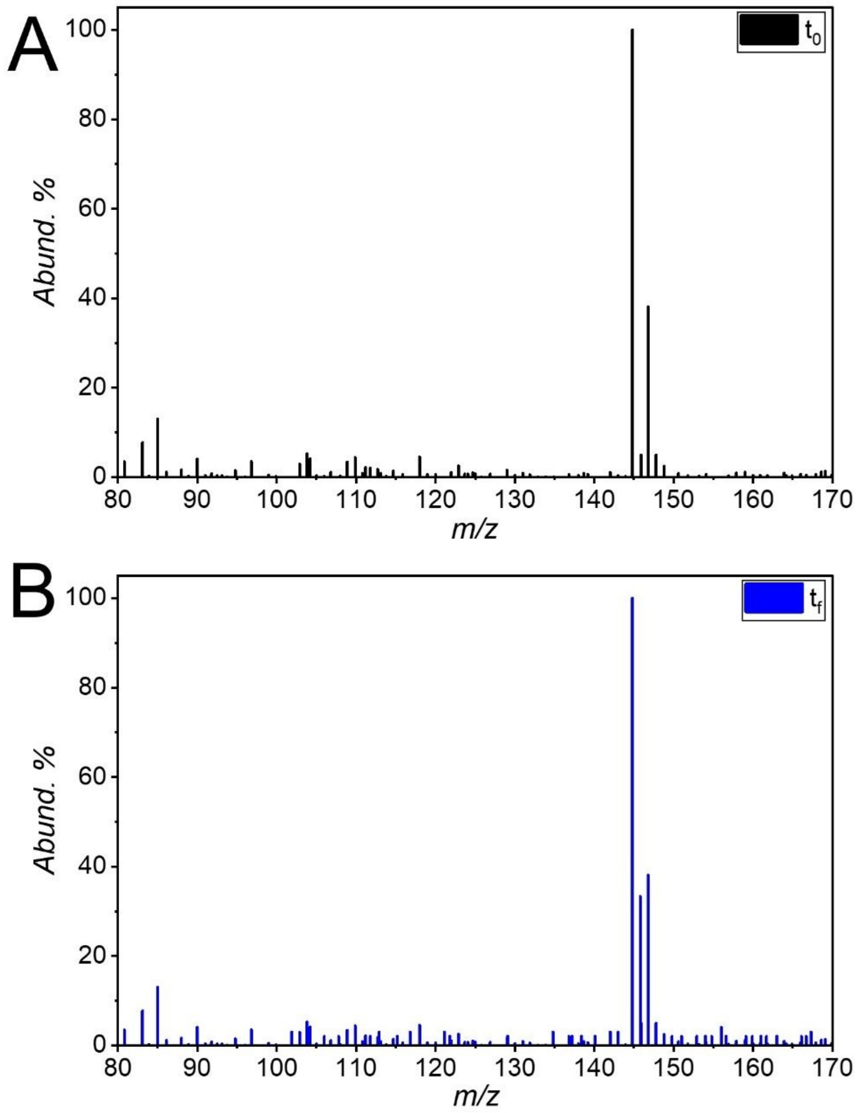

Figure A39. ESI(+) mass spectrum of (S)-1-(5-Chlorothiophen-3-yl)ethanol (A) before and (B) after HIE, showing a +1 Da shift of the molecular ion consistent with mono-deuteration.

13.6.1.11 LC–MS characterization of deuterated (*R*)-1-(2-Furyl)ethanol (C_2)

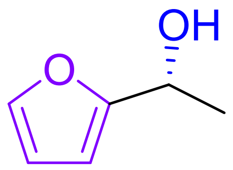

Table A10. LC–MS analysis method for (*R*)-1-(2-Furyl)ethanol.

Parameter	Setting
Mobile phase A	H_2O + 0.1% formic acid
Mobile phase B	ACN + 0.1% formic acid
Flow rate / mL·min^{-1}	0.40 mL·min^{-1}
Column temperature / °C	40
Injection volume / µL	5 µL
Gradient	0–2 min, 90:10 (A:B); 2–6 min, linear to 70:30 (A:B); 6–9 min, linear to 55:45 (A:B); 9–10 min, 55:45 (A:B); 10–11 min, linear to 90:10 (A:B); 11–12 min, 90:10 (A:B).
DAD / nm	214
Ionization	ESI$^+$
Scan range / m/z	80–125

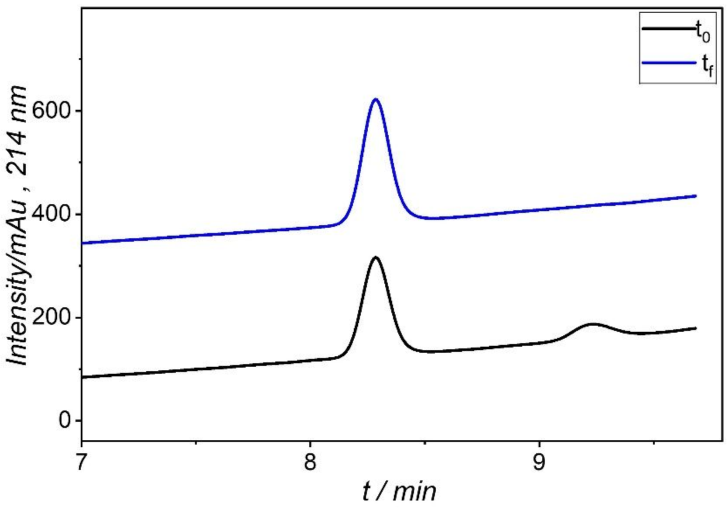

Figure A40. LC–DAD chromatograms (210 nm) of (R)-1-(2-Furyl)ethanol recorded at the start (t_0, black) and after electrolysis (t_f, blue). The alcohol elutes at t□ ≈ 8.2 min, while the ketone formed during HIE elutes at t□ ≈ 9.25 min.

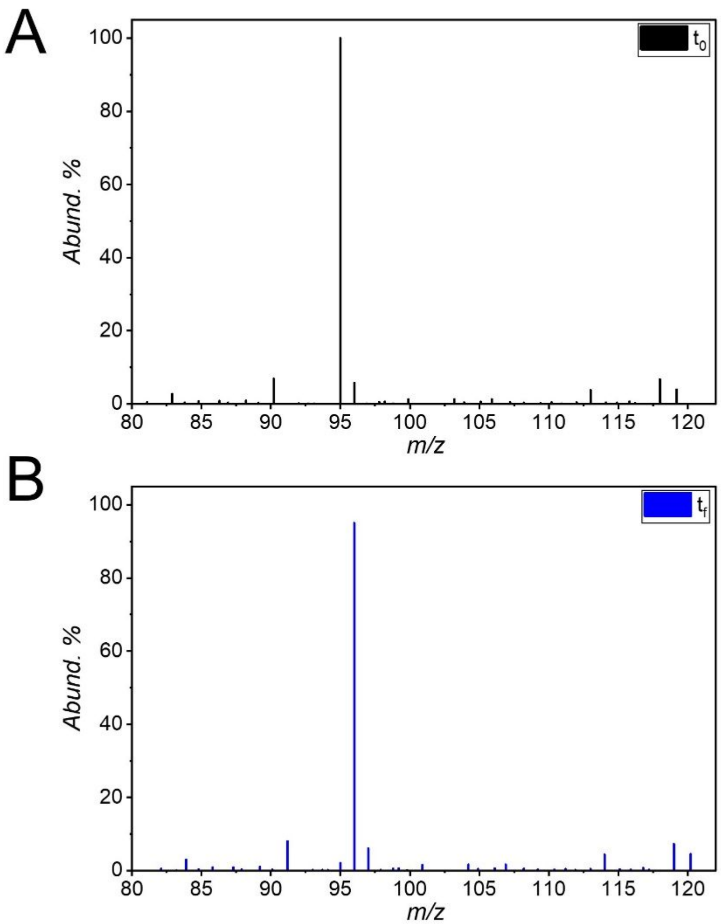

Figure A41. ESI(+) mass spectrum of (R)-1-(2-Furyl)ethanol (A) before and (B) after HIE, showing a +1 Da shift of the molecular ion consistent with mono-deuteration.

13.6.1.12 LC–MS characterization of deuterated (*R*)-1-(pyridinyl)ethanol (C_3)

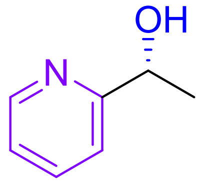

Table A11. LC–MS analysis method for (R)-1-(pyridinyl)ethanol.

Parameter	Setting
Mobile phase A	H_2O + 0.1% formic acid
Mobile phase B	ACN + 0.1% formic acid
Flow rate / mL·min^{-1}	0.40 mL·min^{-1}
Column temperature / °C	40
Injection volume / µL	5 µL
Gradient	0–2 min, 90:10 (A:B); 2–6 min, linear to 70:30 (A:B); 6–9 min, linear to 55:45 (A:B); 9–10 min, 55:45 (A:B); 10–11 min, linear to 90:10 (A:B); 11–12 min, 90:10 (A:B).
DAD / nm	210
Ionization	ESI$^+$
Scan range / m/z	80–125

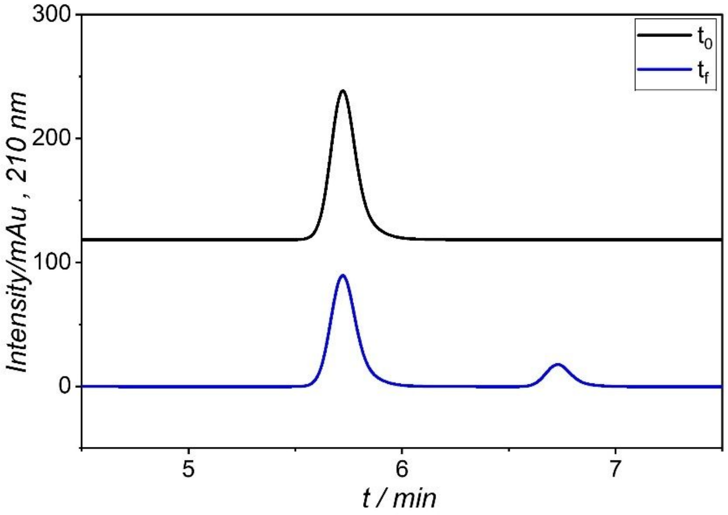

Figure A42. LC–DAD chromatograms (210 nm) of (*R*)-1-(pyridinyl)ethanol recorded at the start (t_0, black) and after electrolysis (t_f, blue). The alcohol elutes at t□ ≈ 5.7 min, while the ketone formed during HIE elutes at t□ ≈ 6.8 min.

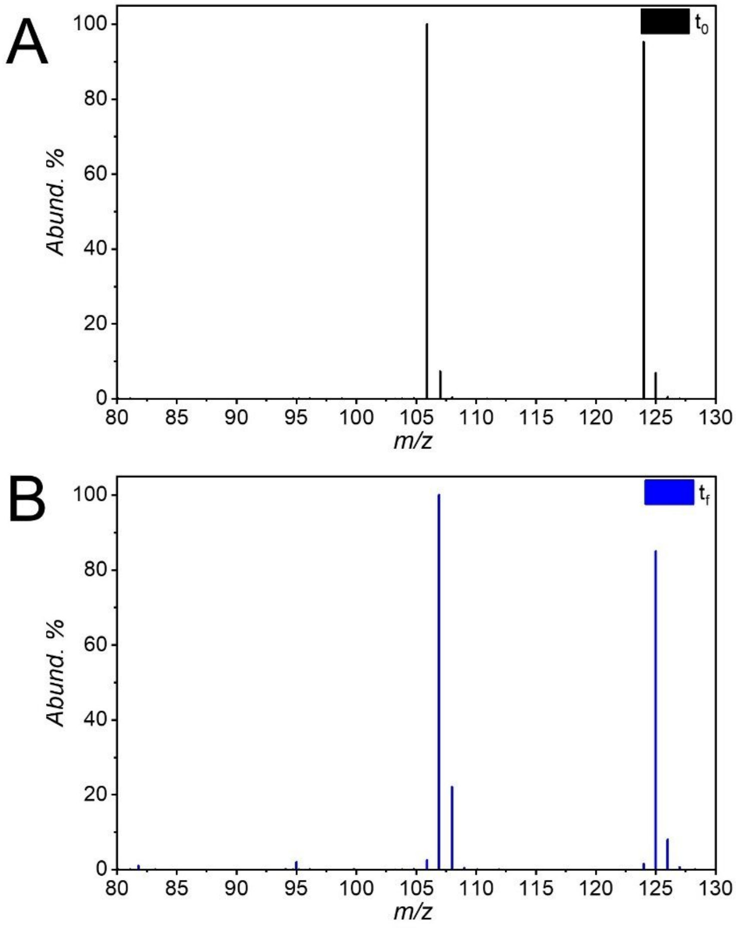

Figure A43. ESI(+) mass spectrum of (*R*)-1-(pyridinyl)ethanol (A) before and (B) after HIE, showing a +1 Da shift of the molecular ion consistent with mono-deuteration.

13.6.1.13 LC–MS characterization of deuterated (*R*)-propanolol (D_1)

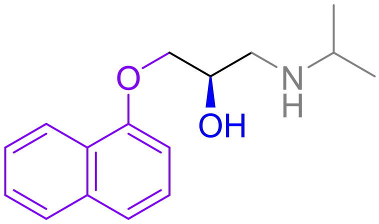

Table A12. LC–MS analysis method for (*R*)-propanolol.

Parameter	Setting
Mobile phase A	H_2O + 0.1% formic acid
Mobile phase B	ACN + 0.1% formic acid
Flow rate / mL·min⁻¹	0.40 mL·min⁻¹
Column temperature / °C	30
Injection volume / µL	3 µL
Gradient	0–2 min, 90:10 (A:B); 2–6 min, 70:30 (A:B); 6–8 min, 60:40 (A:B); 8–9 min, 55:45 (A:B); 9–10 min, 55:45 (A:B); 10–11 min, 90:10 (A:B); 11–12 min, 90:10 (A:B).
DAD / nm	230
Ionization	ESI^+
Scan range / m/z	200-270

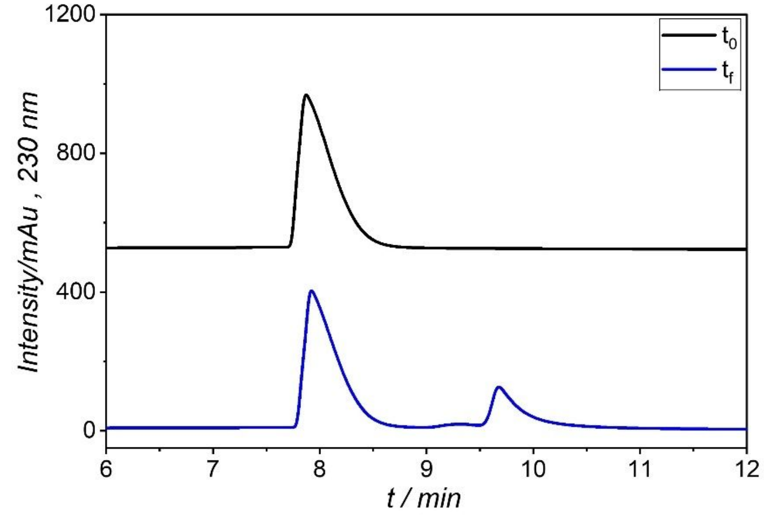

Figure A44. LC–DAD chromatograms (210 nm) of (*R*)-propranolol recorded at the start (t_0, black) and after electrolysis (t_f, blue). The alcohol elutes at t□ ≈ 8 min, while the ketone formed during HIE elutes at t□ ≈ 9.7 min.

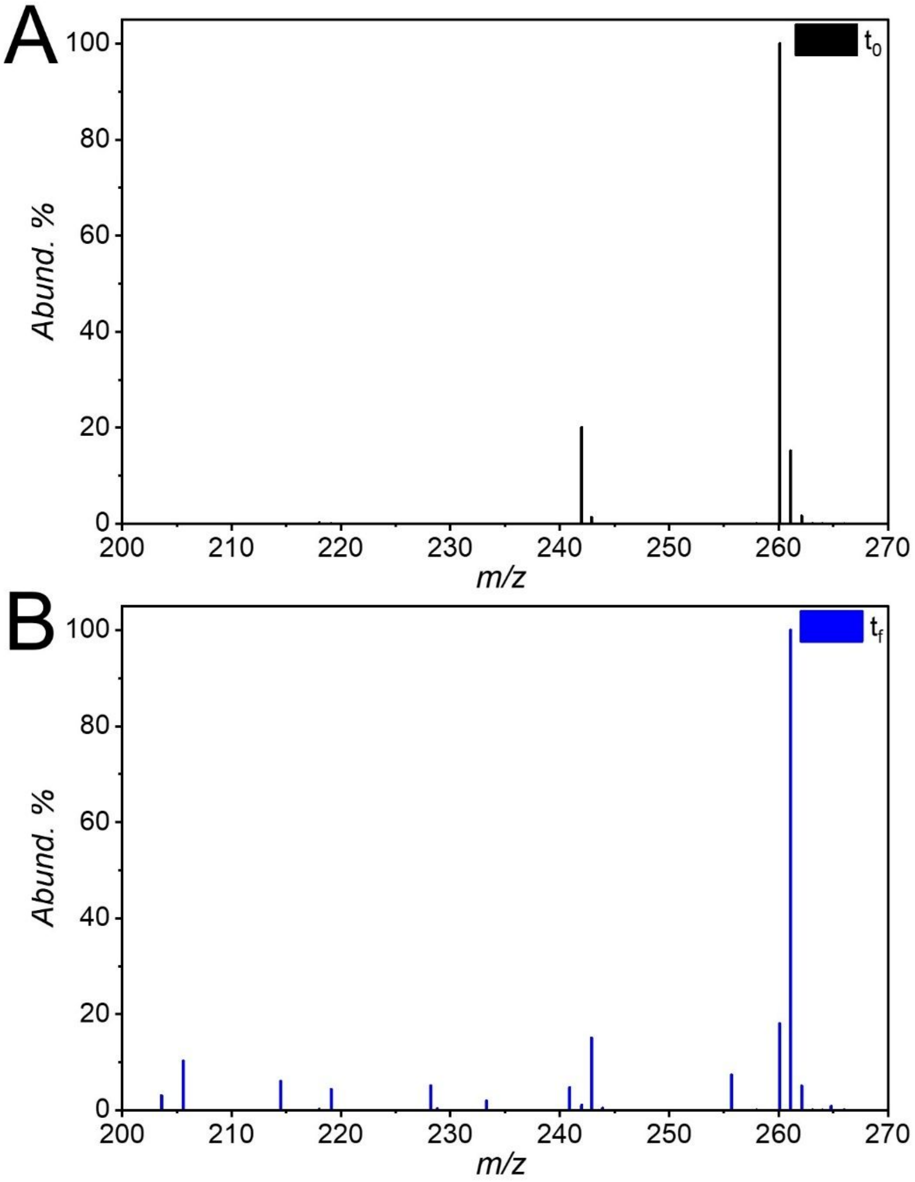

Figure A45.ESI(+) mass spectrum of (R)-propranolol (A) before and (B) after HIE, showing a +1 Da shift of the molecular ion consistent with mono-deuteration.

13.6.2 ^{1}H NMR

^{1}H NMR analysis was performed on a representative subset of four substrates to confirm potential HIE. The remaining substrates were characterized by LC–MS analysis, which provides consistent evidence of selective deuterium incorporation across the substrate scope.

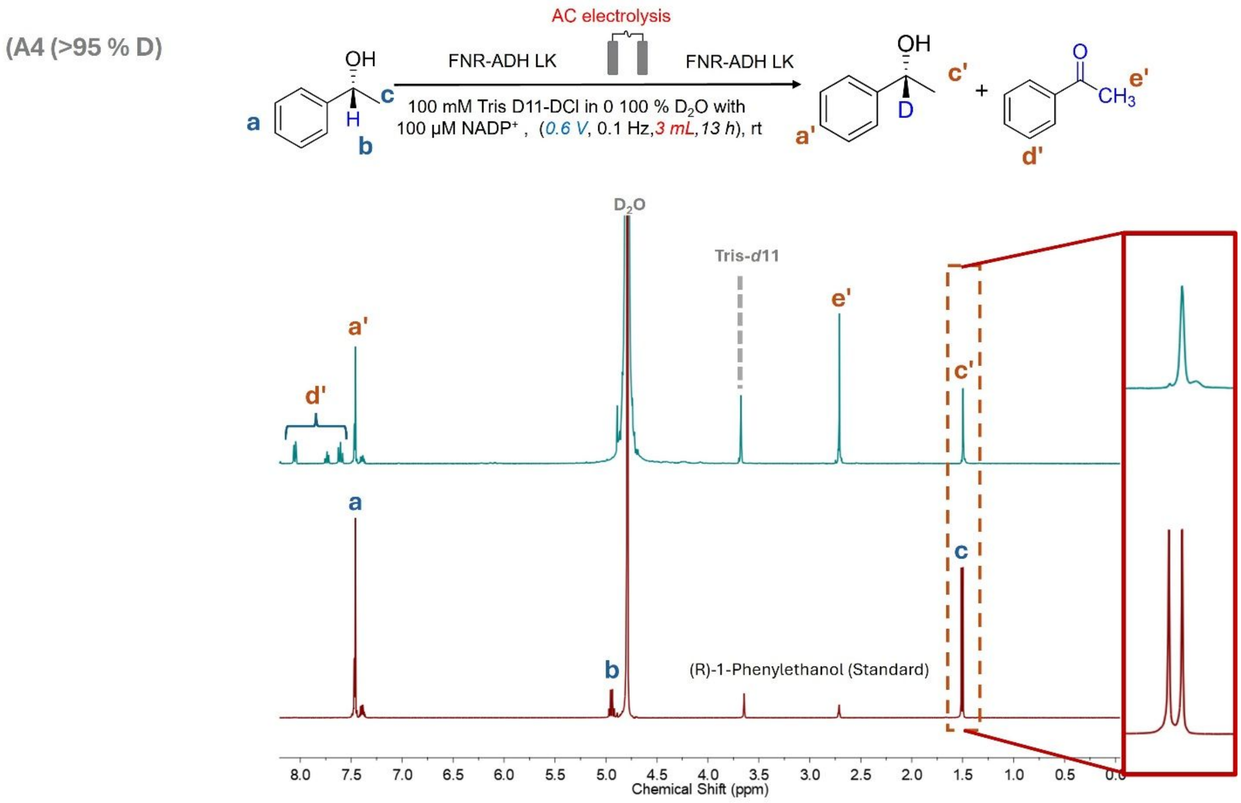

Figure A 46. ^{1}H NMR analysis of electroenzymatic HIE of A_4 under AC electrolysis conditions. The reaction was performed in 100 mM Tris-d$_{11}$–DCl buffer (pD 9) containing 100 µM NADP^{+} and 5 mM $\mathbf{A_4}$ in 100% D_2O using FNR and ADH LK (0.6 V, 0.1 Hz, 3 mL, 13 h, room temperature). The lower spectrum corresponds to the $\mathbf{A_4}$ standard, while the upper spectrum shows the reaction mixture after electrolysis. Aromatic protons (**a / a′**) appear as multiplets at δ ≈ 7.2–7.4 ppm. In the starting material, the methine proton at the stereogenic center (**b**) is observed as a quartet at δ ≈ 4.9–5.0 ppm, coupled to the methyl group (**c**), which appears as a doublet at δ ≈ 1.45 ppm. After electrolysis in D_2O, the methine signal (**b**) is significantly attenuated or absent due to replacement of the proton by deuterium, and the corresponding methyl resonance (**c′**) collapses from a doublet to a singlet. A signal assigned to the methyl group

of the ketone byproduct (**e′**, acetophenone) is observed at δ ≈ 2.55–2.60 ppm. These spectral changes confirm efficient hydrogen–deuterium exchange at the stereogenic center (>95% D incorporation).

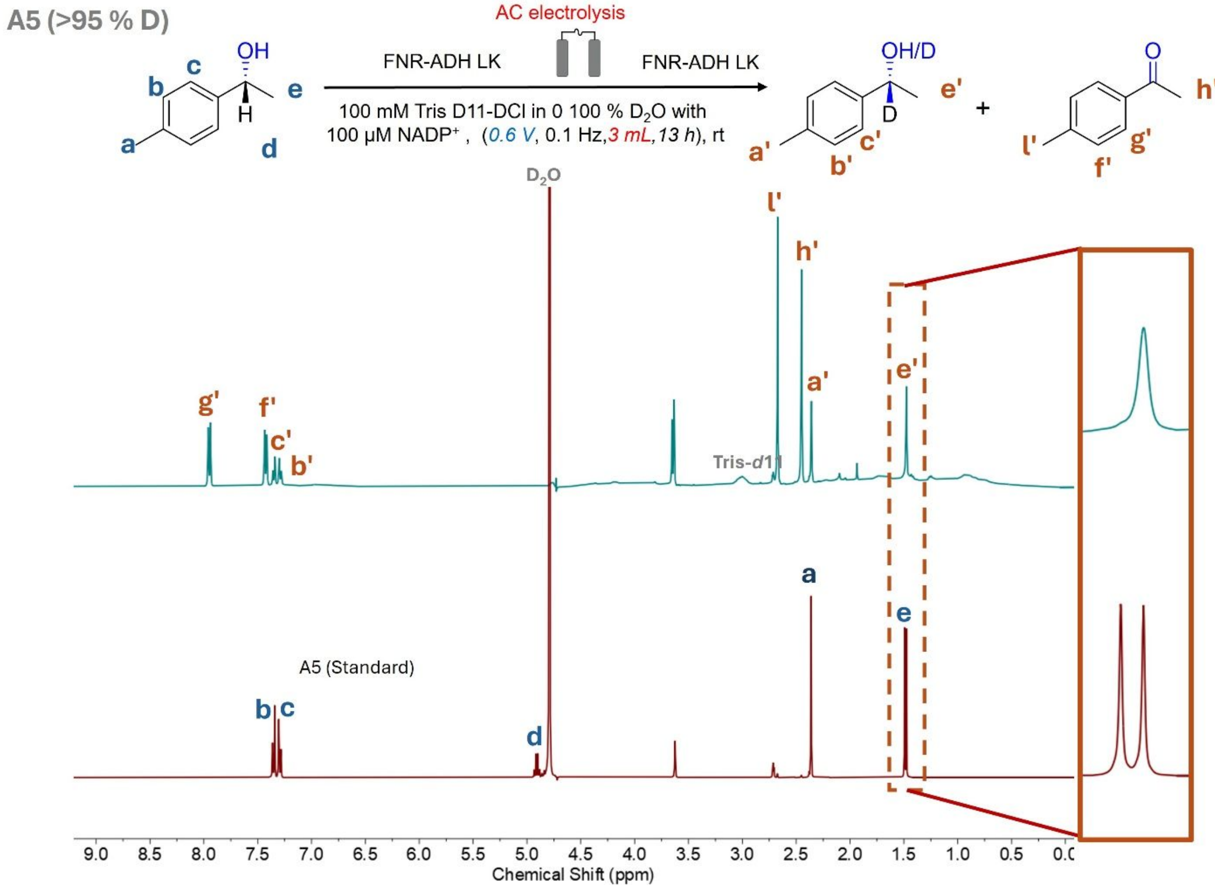

Figure A 47. ¹H NMR analysis of electroenzymatic HIE of A_5 underAC electrolysis conditions. The reaction was performed in 100 mM Tris-d_{11}–DCl buffer (pD 9) containing 100 100 μM $NADP^+$ and 5 mM $\mathbf{A_5}$ in 100% D_2O using FNR and ADH LK (0.6 V, 0.1 Hz, 3 mL, 14 h, room temperature). The lower spectrum corresponds to the $\mathbf{A_5}$ standard, while the upper spectrum shows the reaction mixture after electrolysis. Aromatic protons of the substituted phenyl ring (**b, c → b′, c′**) appear as multiplets at δ ≈ 7.0–7.6 ppm. The para-methyl substituent attached to the phenyl ring (**a → a′**) is observed as a singlet at δ ≈ 2.2–2.3 ppm. In the starting material, the methine proton at the stereogenic center (**d**) appears at δ ≈ 4.9–5.0 ppm and the adjacent methyl group (**e**) appears as a doublet at δ ≈ 1.45 ppm. After electrolysis in D_2O, the methine resonance (**d**) is strongly attenuated and the methyl signal (**e′**) collapses to a singlet. Signals corresponding to the ketone are observed at δ ≈ 2.5–2.6 ppm (**h′**) together with aromatic resonances (**f′, g′**).

These spectral changes confirm efficient hydrogen–deuterium exchange at the stereogenic center (>95% D incorporation).

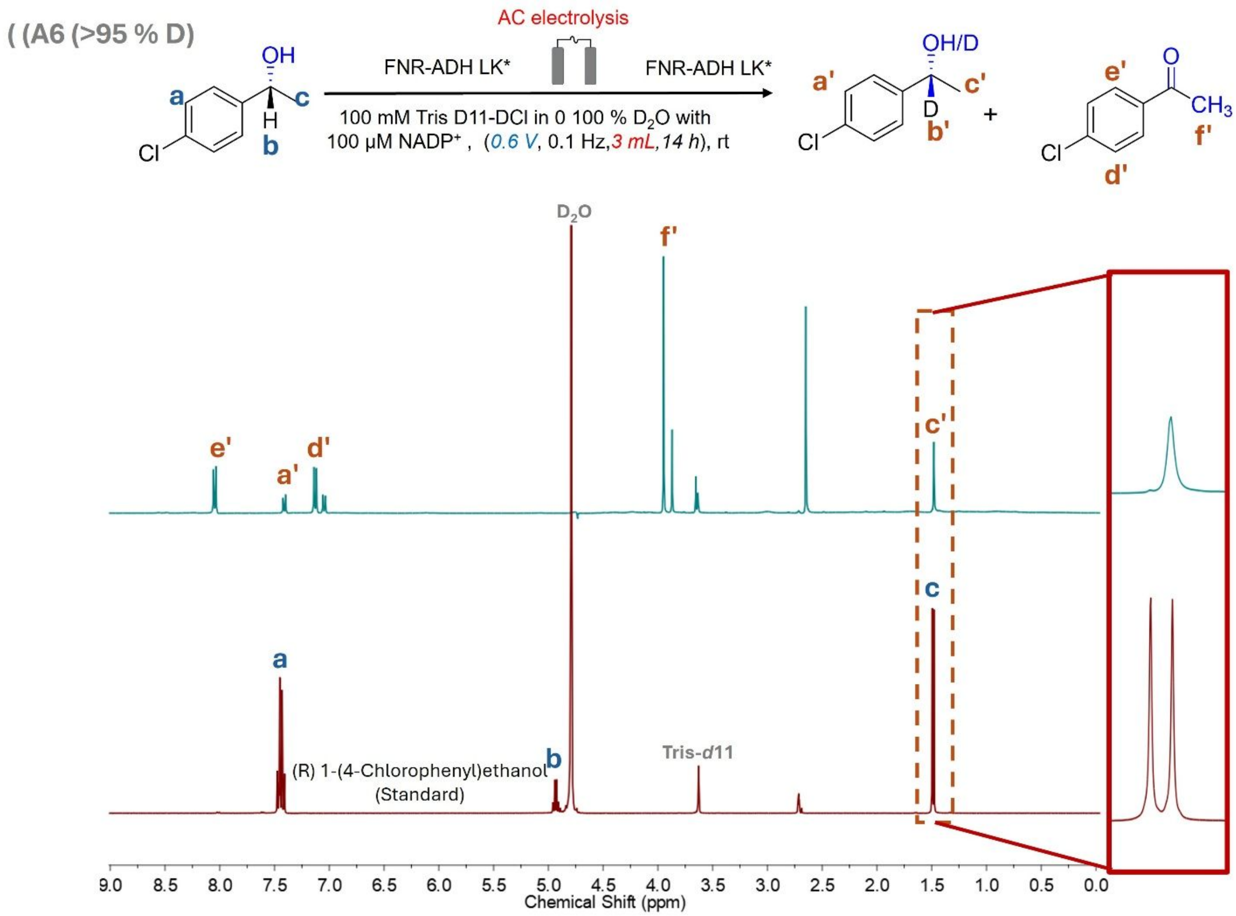

Figure A 48. ^{1}H NMR analysis of electroenzymatic HIE of A_6 under AC electrolysis conditions. The reaction was performed in 100 mM Tris-d_{11}–DCl buffer (pD 9) containing 100 100 µM $NADP^+$ and 5 mM $\mathbf{A_6}$ in 100% D_2O using FNR and ADH LK (0.6 V, 0.1 Hz, 3 mL, 14 h, room temperature). The lower spectrum corresponds to the authentic (**A6** standard, while the upper spectrum shows the reaction mixture after electrolysis. Aromatic protons of the chlorophenyl ring (**a** → **a′**) appear as multiplets at δ ≈ 7.1–7.5 ppm. The residual D_2O signal is observed at δ ≈ 4.8 ppm, and signals from the Tris-d_{11} buffer appear in the region δ ≈ 3.6–3.8 ppm.

In the starting material, the methine proton at the stereogenic center (**b**) is observed at δ ≈ 4.9–5.0 ppm and is coupled to the adjacent methyl group (**c**), which appears as a doublet at δ ≈ 1.45 ppm. After electrolysis in D_2O, the methine signal (**b**) is significantly attenuated or absent due to replacement of the proton by deuterium, and the corresponding methyl resonance (**c′**) collapses from a doublet to a singlet.

Additional signals corresponding to the ketone are observed in the reaction mixture: aromatic protons (**d′, e′**) appear in the aromatic region (δ ≈ 7.2–7.8 ppm), and the methyl group of the acetyl moiety (**f′**) is observed as a singlet at δ ≈ 2.5–2.6 ppm. These spectral changes confirm efficient hydrogen–deuterium exchange at the stereogenic center (>95% D incorporation).

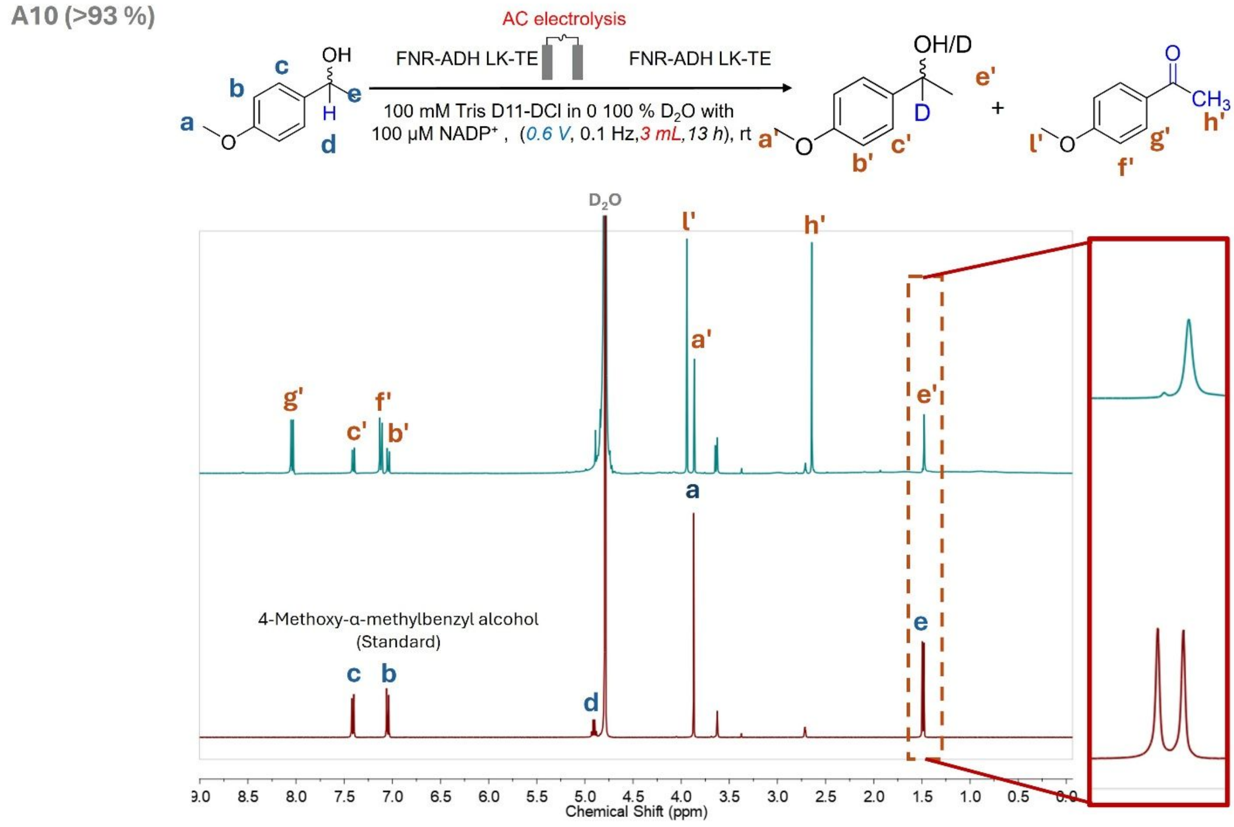

Figure A 49. **¹H NMR analysis of electroenzymatic HIE of A10 under AC electrolysis conditions using both ADH LK and ADH TE.** The reaction was performed in 100 mM Tris-d_{11}–DCl buffer (pD 9) containing 100 µM $NADP^+$ and 5 **mM A_{10}** in 100% D_2O using FNR in combination with the complementary alcohol dehydrogenases ADH LK and ADH TE (0.6 V, 0.1 Hz, 3 mL, 13 h, room temperature). The lower spectrum corresponds to **A10** alcohol standard, while the upper spectrum shows the reaction mixture after electrolysis. Aromatic protons of the methoxy-substituted phenyl ring (**b, c → b′, c′**) appear as multiplets at δ ≈ 6.8–7.4 ppm, and the methoxy substituent (**a → a′**) is observed as a singlet at δ ≈ 3.7–3.8 ppm. The residual D_2O signal is observed at δ ≈ 4.8 ppm, and signals from the Tris-d_{11} buffer appear in the region δ ≈ 3.6–3.8 ppm.

In the starting material, the methine proton at the stereogenic center (**d**) is observed at δ ≈ 4.9–5.0 ppm and is coupled to the adjacent methyl group (**e**), which appears as a doublet at δ ≈ 1.45 ppm. After electrolysis in D_2O, the methine signal (**d**) is significantly attenuated or absent due to replacement of the proton by deuterium, and the corresponding methyl resonance (**e′**) collapses from a doublet to a singlet.

Additional signals corresponding to the ketone are observed in the reaction mixture: aromatic protons (**f′, g′**) appear in the aromatic region, the methoxy substituent of the ketone (**l′**) is observed at δ ≈ 3.7–3.8 ppm, and the methyl group of the acetyl moiety (**h′**) is observed as a singlet at δ ≈ 2.5–2.6 ppm. The spectral changes confirm efficient hydrogen–deuterium exchange at the stereogenic center (>93% D incorporation). The use of both ADH LK and ADH TE enables stereocomplementary oxidation and reduction steps, demonstrating that deuterium incorporation occurs for both enantiomers of the racemic substrate in this methoxy-substituted system.

13.6.3 Charge passed during AC electrolysis

To estimate the electrical input associated specifically with substrate turnover during AC electrolysis, the total charge was destimated after correction for the background current measured in the presence of immobilized enzymes prior to cofactor and substrate addition. Under these conditions, the background current reflects non-catalytic contributions arising from capacitive charging and baseline enzyme–electrode processes occurring in the absence of substrate.

Therefore, the substrate-dependent current was defined as the difference between the current recorded during electrolysis in the presence of substrate and the background current measured under identical conditions with the immobilized enzymes but without substrate as shown in **Figure A16.**

$$\mathbf{I_{substrate(t)} = I_{with\ substrate(}t)(I_t) - I_{without\ substrate}\ (I_0)}$$

For discretely recorded data points, the charge was calculated using:

$$\mathbf{Q\_substrate \approx \Sigma\ |I\ t,i - I_0| \times \Delta t_i}$$

The electrolysis experiments performed in a 200 μL reaction volume were conducted by mixing the catalyst, substrate, and cofactor at the start of the experiment. Under these batch conditions, the catalytic current decreased progressively during electrolysis as the substrate was consumed. The reaction was considered complete when the current reached a stable steady-state value.

The initial current at the start of electrolysis was defined as I_0, and the stabilized current at the end of the reaction was defined as I_f, representing the background current of the electrode–enzyme system in the absence of substrate turnover (**Figure A50**). The charge associated with substrate

conversion was calculated by integrating the difference between the instantaneous current and the final steady-state current over the duration of the reaction:

$Q = \int_{t_0}^{t_f} |I(t) - I_f|\, dt$ where Q is the total charge passed (C), $I(t)$ is the measured current at time t, and I_f is the final stabilized current after completion of the reaction.

The resulting value (**Table A14**) represents the total electrical input delivered to the electroenzymatic system during the specified electrolysis period under the defined reaction conditions.

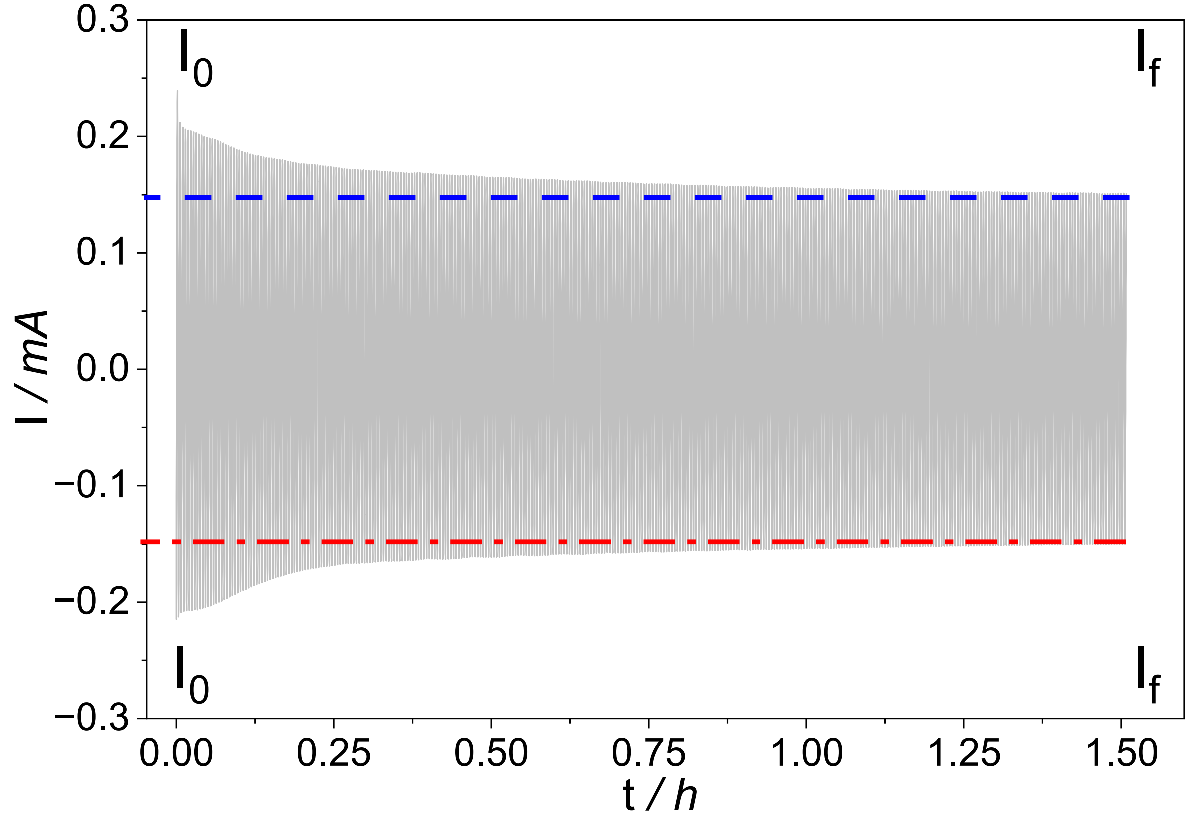

Figure A 50. Determination of reaction completion and charge calculation during AC electrolysis in a 200 µL batch system. Representative current–time trace recorded during alternating-current electrolysis with substrate, cofactor, and immobilized FNR and ADH present from the start of the reaction. The catalytic current decreases as the substrate is consumed and stabilizes at a constant value upon reaction completion. The blue dashed line indicates the initial current (I_0), and the red dashed line indicates the final stabilized current (I_f), representing the background current of the enzyme-modified electrode. The charge associated with substrate conversion was determined by integrating the absolute difference between the instantaneous current and I_f over the duration of electrolysis.

Table A 13. Estimated Charge Passed during ACeElectrolysis for substrate scope experiments in a 200 µL FNR/ADH-LK system (Figure 6)

**Substrate**	**Q (C)**	**Substrate**	**Q (C)**	**Substrate**	**Q (C)**	**Substrate**	**Q (C)**
**A1**	0.31	**A6**	0.45	**A11**	0.45	**C1**	0.56
**A2**	0.44	**A7**	0.47	**A12**	0.53	**C2**	0.38

**A3**	0.35	**A8**	0.58	**A13**	0.6	**C3**	0.4
**A4**	0.39	**A9**	0.43	**B1**	0.73	**D1**	0.63
**A5**	0.36	**A10**	0.36	**B2**	0.69		

13.7 DC electrolysis for stereochemical inversion and deracemization

13.7.1 R→S enantiomeric inversion via direct-current electrolysis

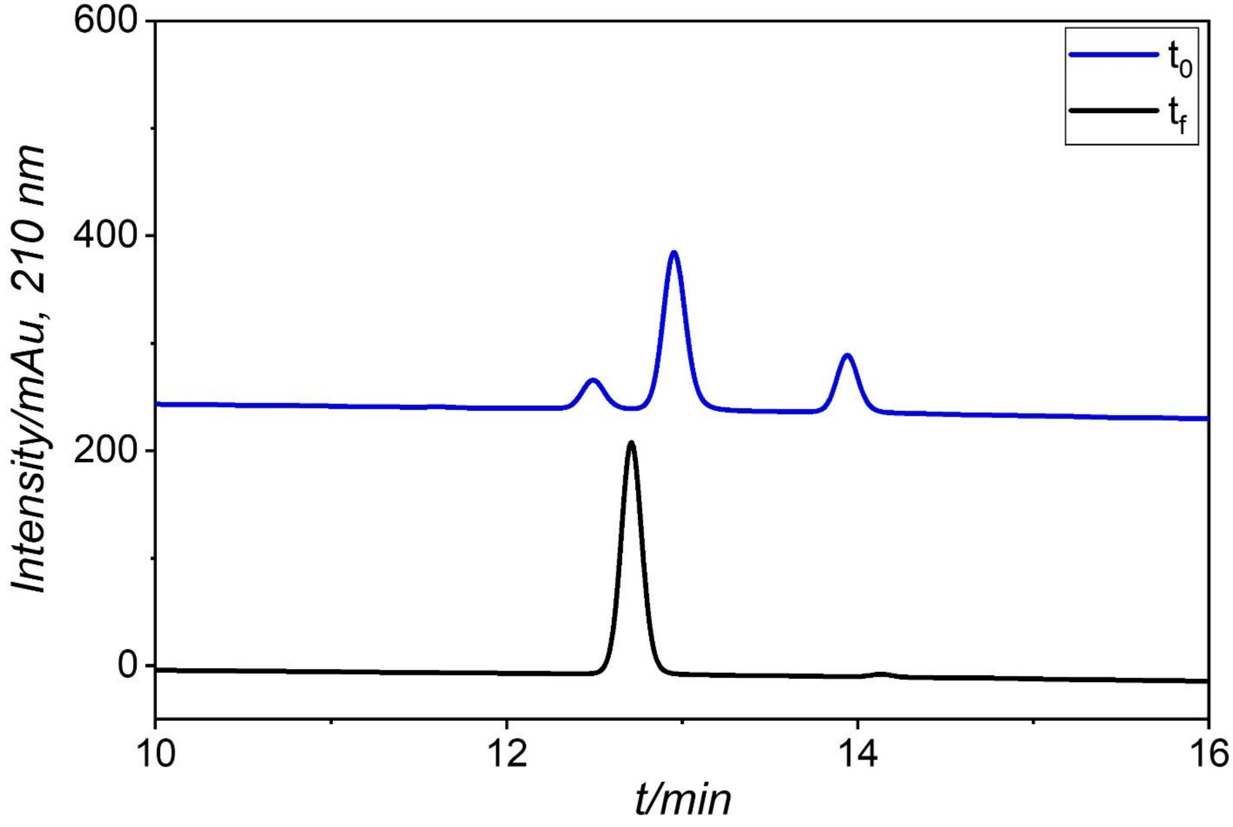

Figure A51. LC–UV chromatograms recorded at 210 nm for the R→S enantiomeric inversion experiment under direct-current electrolysis. The chromatogram acquired at the beginning of the reaction (t_0, blue) displays the initial enantiomeric distribution (**1′**), whereas the trace obtained after electrolysis (t_f) shows a dominant peak corresponding to the S-enantiomer **2′**, evidencing enantioselective inversion under DC operation.

13.7.2 S→R enantiomeric inversion via direct-current electrolysis

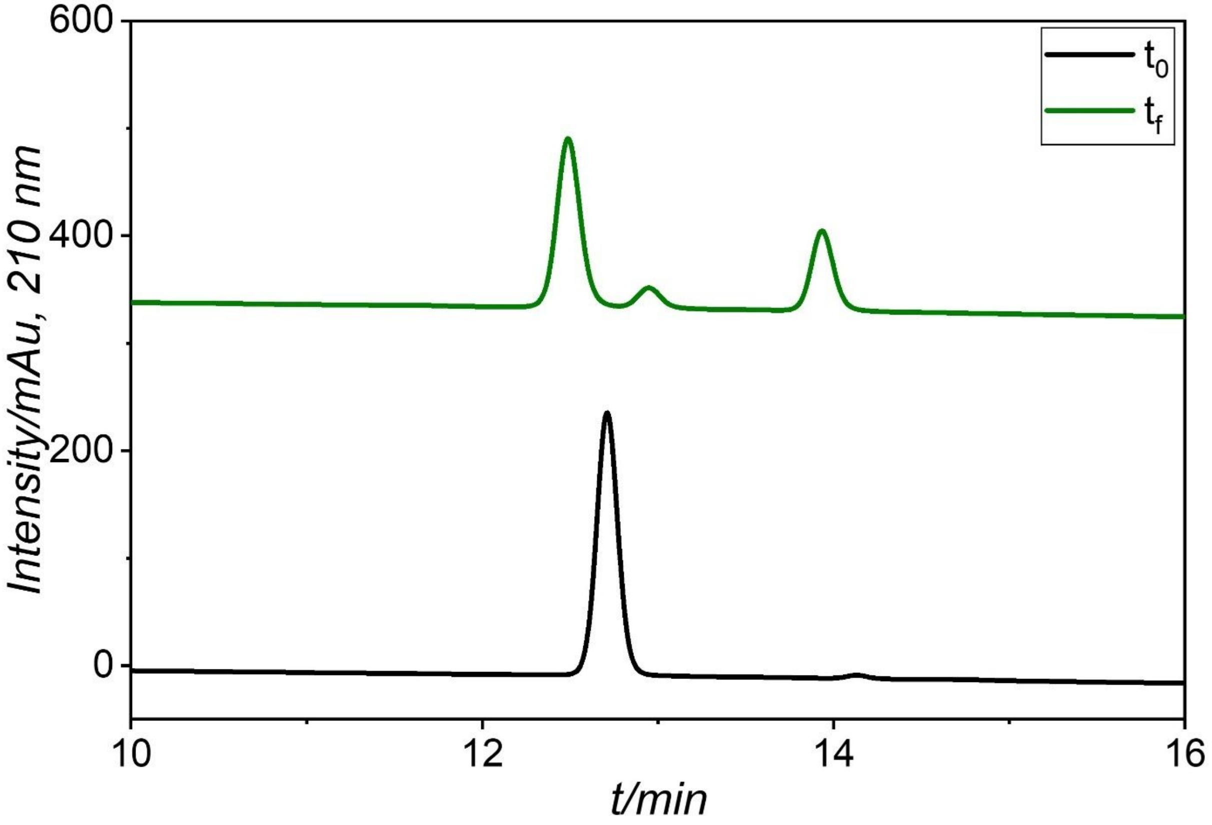

Figure A52. LC–UV chromatograms recorded at 210 nm for the S→R enantiomeric inversion experiment under direct-current electrolysis. The chromatogram acquired at the beginning of the reaction (t_0) displays the initial enantiomeric distribution (**2′**), whereas the trace obtained after electrolysis (t_f) shows a dominant peak corresponding to the R-enantiomer (**1′**), evidencing enantioselective inversion under DC operation.

13.7.3 Racemate → R deracemization via direct-current electrolysis

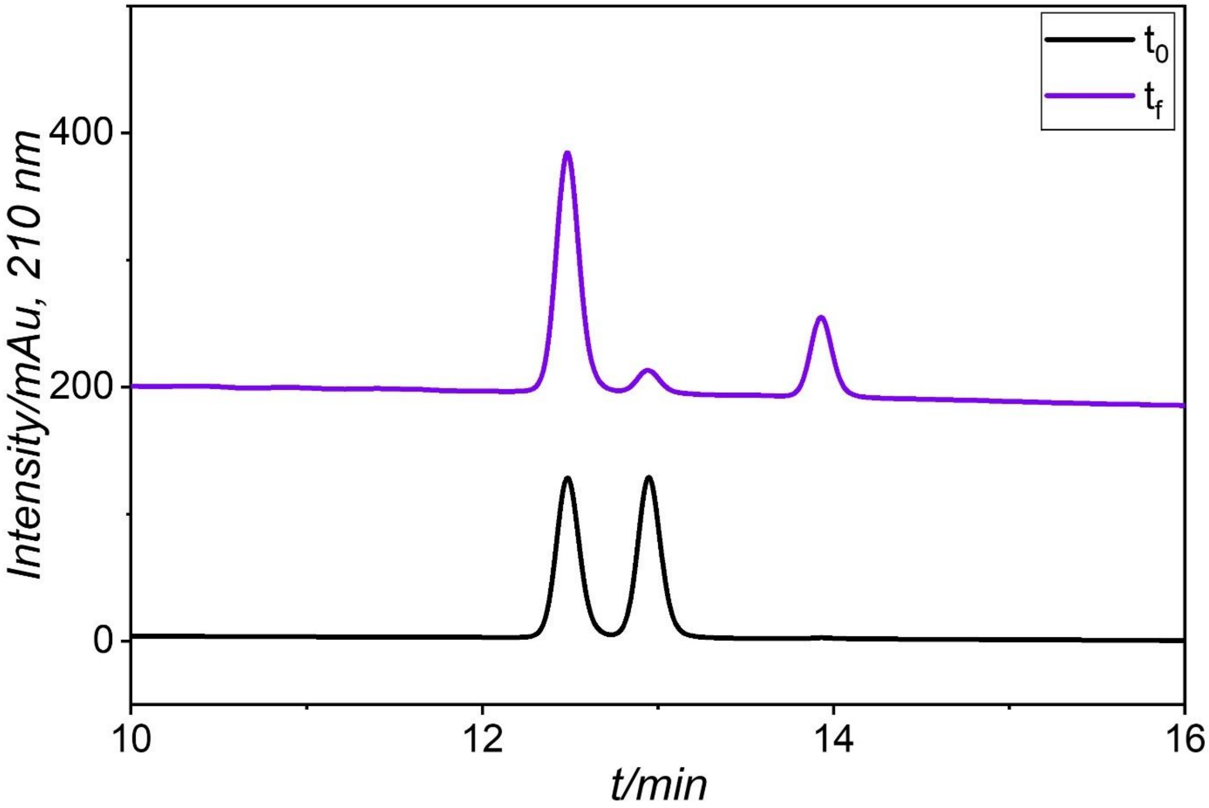

Figure A53. LC–UV chromatograms recorded at 210 nm for the rac→R enantiomeric deracemization experiment under direct-current electrolysis. The chromatogram acquired at the beginning of the reaction (t_0) displays the initial enantiomeric distribution (**3′**), whereas the trace obtained after electrolysis (t_f) shows a dominant peak corresponding to the R-enantiomer (**1′**).

13.7.4 Racemate → S deracemization via direct-current electrolysis

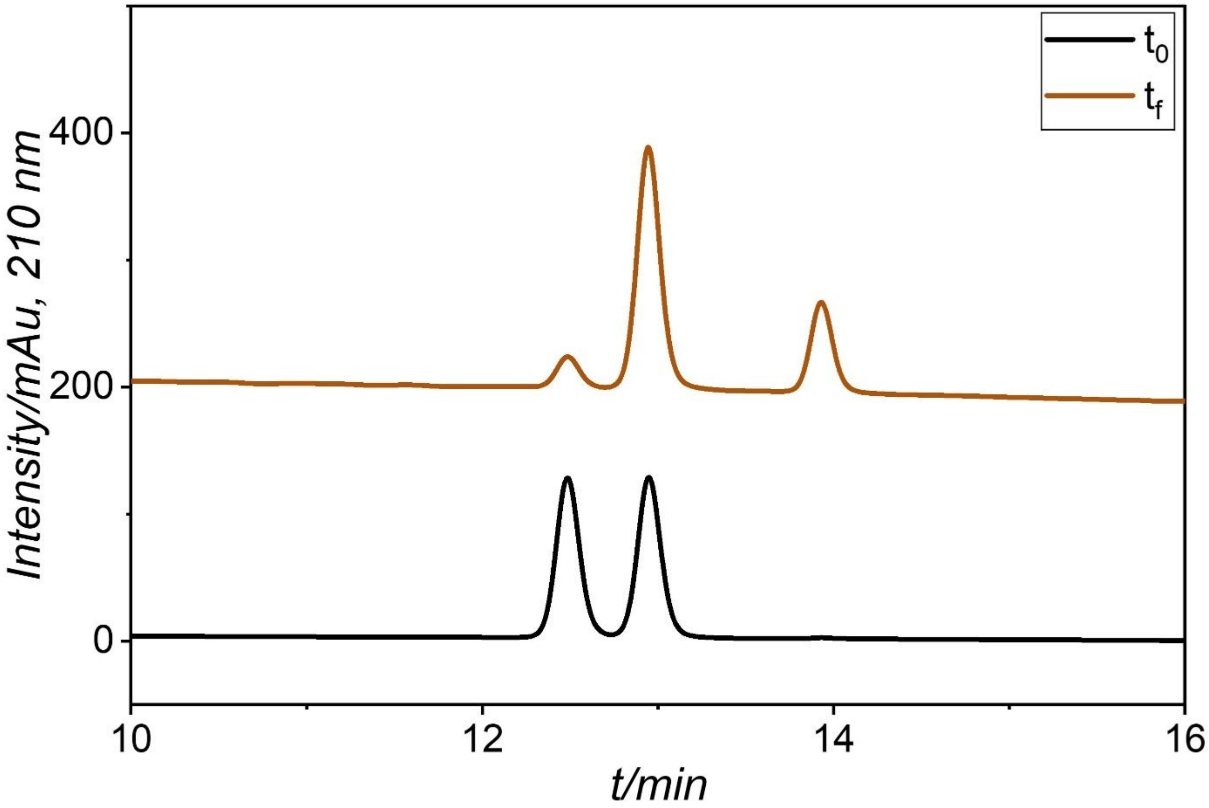

Figure A54. LC–UV chromatograms recorded at 210 nm for the rac→S enantiomeric deracemization experiment under direct-current electrolysis. The chromatogram acquired at the beginning of the reaction (t_0) displays the initial enantiomeric distribution (**3′**), whereas the trace obtained after electrolysis (t_f) shows a dominant peak corresponding to the S-enantiomer (**2′**).

13.7.5 ^{1}H NMR

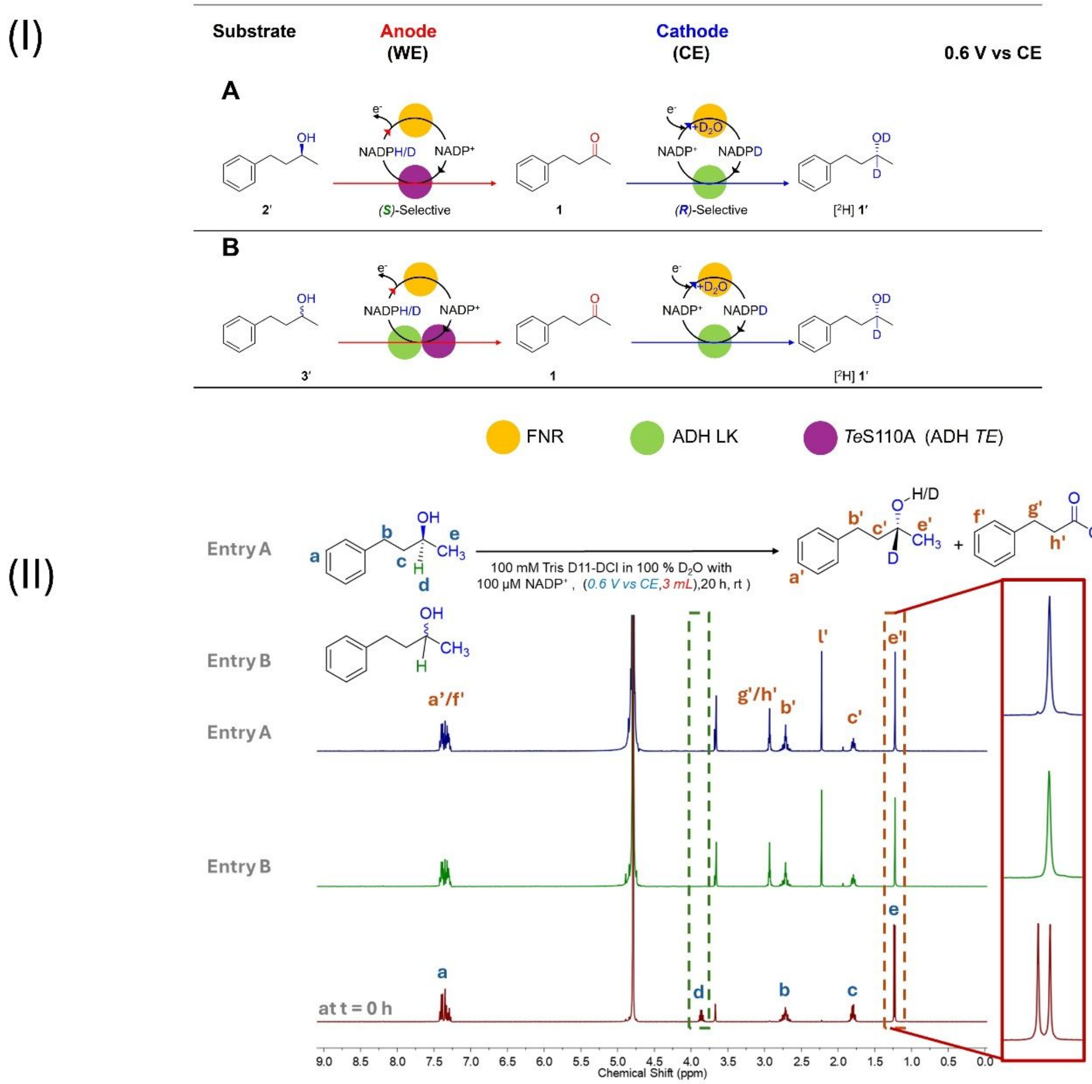

Figure A 55. Electroenzymatic stereochemical inversion and deracemization of secondary alcohols coupled to HIE and corresponding ^{1}H NMR characterization. **I)** Schematic representation of electroenzymatic stereochemical editing under paired electrolysis conditions (0.6 V vs CE). **Entry A:** Stereochemical inversion of t5 mM **2′** to the deuterated (R)-alcohol ([²H]1′). Selective oxidation of the (S)-alcohol to the ketone intermediate (1) occurs at the anode (working electrode, WE) in the presence of FNR and the (S)-selective alcohol dehydrogenase TeS110A (ADH TE). At the cathode (counter electrode, CE), FNR catalyzes regeneration of the reduced nicotinamide cofactor in D_2O, and the (R)-selective alcohol dehydrogenase ADH LK reduces the ketone to the deuterated (R)-alcohol, resulting in

stereochemical inversion accompanied by HIE at the stereogenic center.**Entry B**: Deracemization of racemic alcohol 5 mM **3′** to the deuterated (R)-alcohol ([^{2}H]1′). At the anode (WE), FNR, ADH LK, and ADH TE catalyze oxidation of both enantiomers of the racemic alcohol to the common ketone intermediate (1). At the cathode (CE), FNR and ADH LK selectively reduce the ketone to the (R)-configured deuterated alcohol. II) Representative ^{1}H NMR spectra of reaction mixtures obtained after electrolysis (>95 % D) in 100 mM Tris-d11–DCl buffer (pD 9) containing 100 µM $NADP^+$ in 100% D_2O (0.6 V vs CE, 3 mL, 20 h, room temperature).

13.8 AC electrolysis with FNR-ADH TE@CP-ITO$_m$

13.8.1 Electroenzymatic deuteration of 2′ driven by FNR–ADH TE

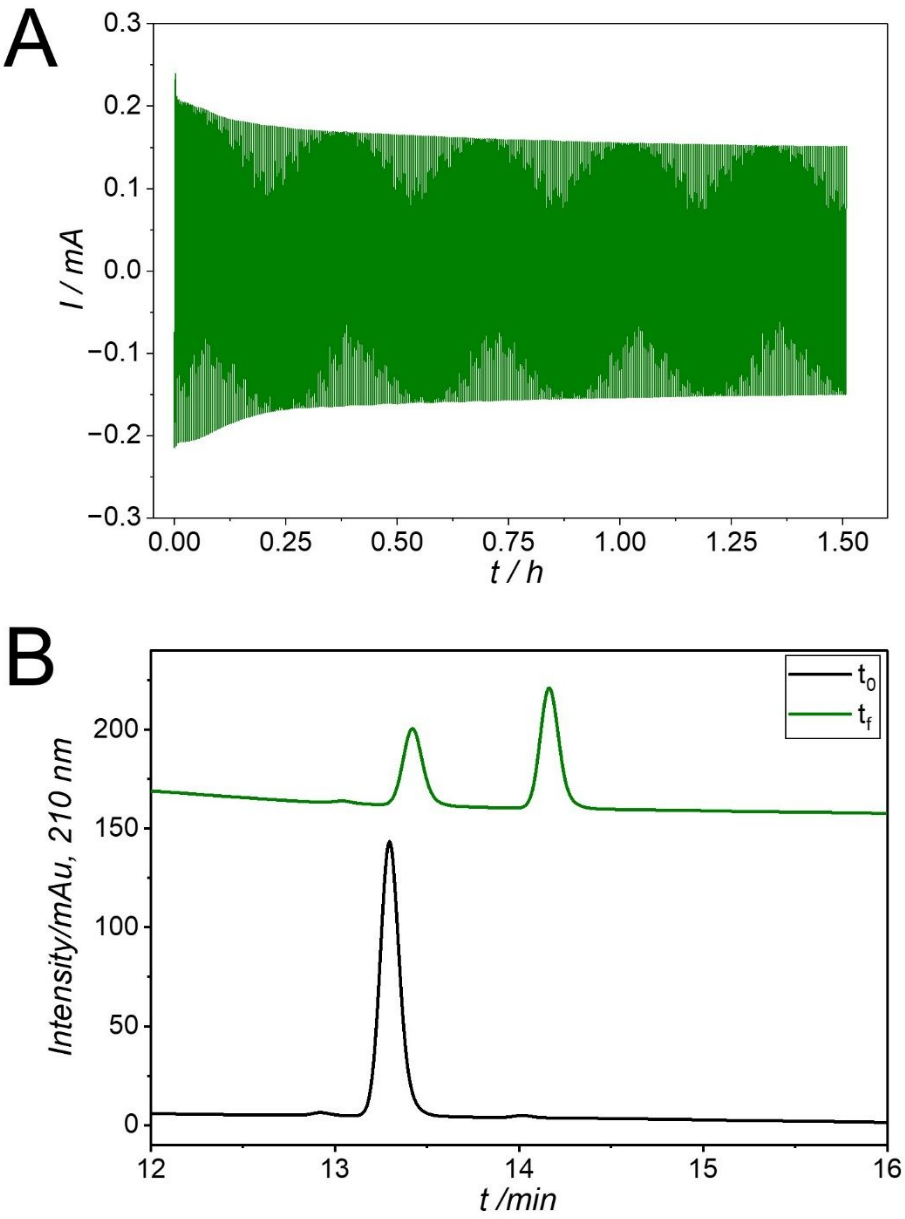

Figure A56. (A) Chronoamperometric response recorded under AC electrolysis during electroenzymatic HIE of **2′** catalyzed by FNR–ADH TE@CP–ITO (B) LC–UV traces (210 nm) of the reaction mixture at the initial time (t_0, black) and after electrolysis (t_f, green).

13.8.2 Electroenzymatic deuteration of 3′ driven by FNR–ADH TE

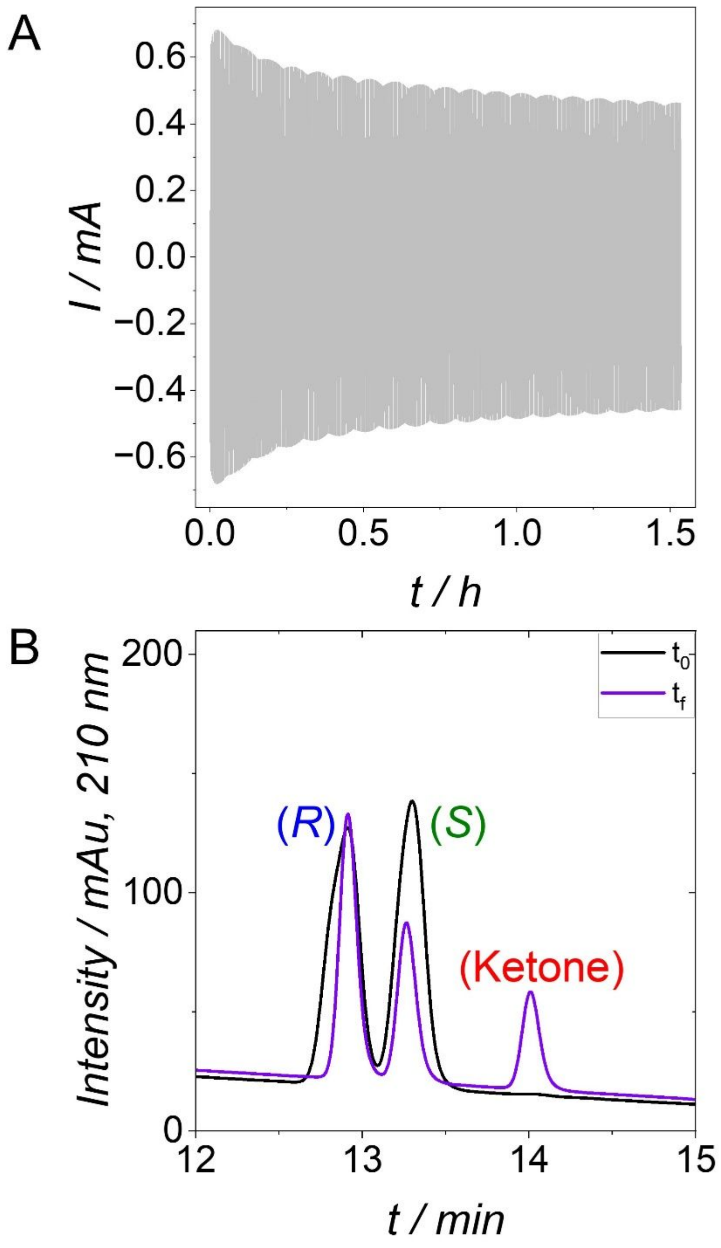

Figure A57. (A) Chronoamperometric response recorded under AC electrolysis during electroenzymatic HIE of **3′** catalyzed by FNR–ADH TE@CP–ITO (B) LC–UV traces (210 nm) of the reaction mixture at the initial time (t_0, black) and after electrolysis (t_f, green).

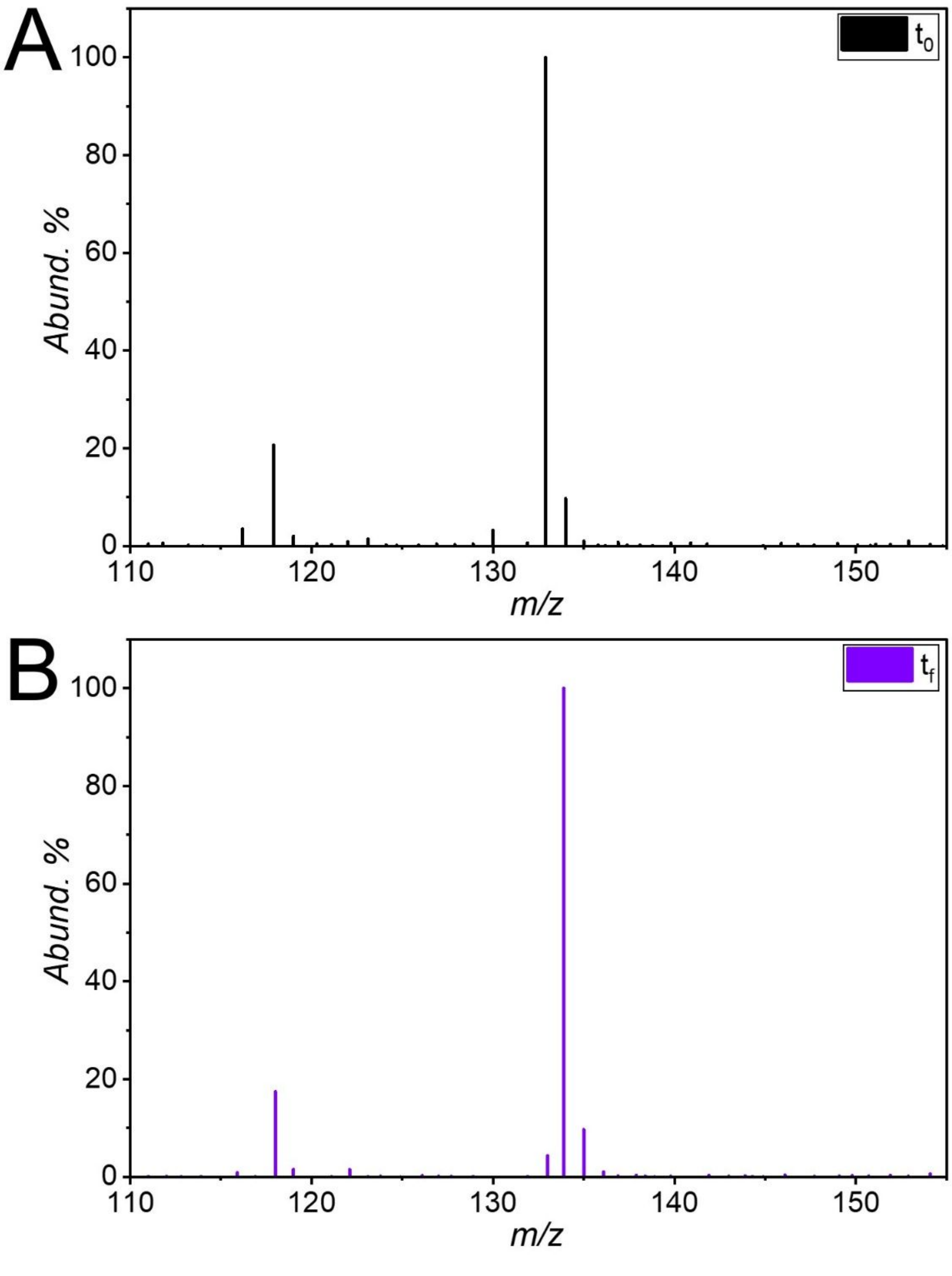

Figure A58. ESI(+) mass spectra of **2′** recorded before (t_0, A) and after electroenzymatic hydrogen–deuterium exchange (t_f, B) catalyzed by FNR–ADH TE@CP–ITO. The post-reaction spectrum exhibits a clear +1 Da shift of the molecular-ion isotopologue relative to the undeuterated reference, consistent with mono-deuteration of the alcohol, while the overall fragmentation pattern remains unchanged.

14 References

(1) El Housseini, W.; Lapicque, F.; Walcarius, A.; Lojou, E.; Rouhier, N.; Etienne, M. Bioelectrochemistry Ferredoxin NADP + Reductase for NADPH and NADH Regeneration in a Flow Bioelectrochemical Reactor ☆. **2025**, *164* (August 2024), 0–8.

(2) Rudzka, A.; Reiter, T.; Kroutil, W.; Borowiecki, P. Bienzymatic Dynamic Kinetic Resolution of Secondary Alcohols by Esterification / Racemization in Water. **2025**. https://doi.org/10.1002/anie.202420133.

(3) Musa, M. M.; Patel, J. M.; Nealon, C. M.; Sup, C.; Phillips, R. S.; Karume, I. Journal of Molecular Catalysis B : Enzymatic Thermoanaerobacter Ethanolicus Secondary Alcohol Dehydrogenase Mutants with Improved Racemization Activity. *J. Mol. Catal. B Enzym.* **2015**, *115*, 155–159. https://doi.org/10.1016/j.molcatb.2015.02.012.

(4) Gerulskis, R.; Minteer, S. D. Terminator: A Software Package for Fast and Local Optimization of His-Tag Placement for Protein Affinity Purification. *ACS Bio & Med Chem Au* **2024**, *5* (1), 55–65. https://doi.org/10.1021/acsbiomedchemau.4c00055.

(5) Gerulskis, R.; Minteer, S. D. Reproducibly Defining Electrode Area of Carbon Paper Electrodes via Machine Cutting and High-Throughput Waxing. *ECS Advances* **2023**, *2* (3), 35501.

(6) Bradshaw, C. W.; Hummel, W.; Wong, C. H. Lactobacillus Kefir Alcohol Dehydrogenase: A Useful Catalyst for Synthesis. *J. Org. Chem.* **1992**, *57* (5), 1532–1536.

(7) Weckbecker, A.; Hummel, W. Cloning, Expression, and Characterization of an (R)-Specific Alcohol Dehydrogenase from Lactobacillus Kefir. *Biocatal. Biotransformation* **2006**, *24* (5), 380–389.
